# Stereoselective electrochemical intramolecular imino-pinacol reaction: a straightforward entry to enantiopure piperazines

**DOI:** 10.3762/bjoc.21.147

**Published:** 2025-09-12

**Authors:** Margherita Gazzotti, Fabrizio Medici, Valerio Chiroli, Laura Raimondi, Sergio Rossi, Maurizio Benaglia

**Affiliations:** 1 Dipartimento di Chimica, Università degli Studi di Milano, via Golgi 19, IT-20133 Milano, Italy

**Keywords:** chiral piperazines, electrosynthesis, flow chemistry, green chemistry, imino-pinacol coupling

## Abstract

The stereoselective electroreductive intramolecular coupling of chiral diimines of aromatic aldehydes with *trans-*1,2-diaminocyclohexane for the synthesis of enantiopure tetrasubstituted piperazines has been investigated by an electrochemical approach. The methodology was successfully developed under both batch and continuous flow conditions, and afforded enantiomerically pure products with complete stereoselectivity. Substrates bearing electron-donating or electron-withdrawing groups on the aromatic rings provided good to excellent yields, indicating that both types of substituents are well tolerated under the reaction conditions. Although modest yields were obtained under flow conditions, the continuous process afforded higher productivities and space-time yields than the batch reactions due to a short residence time. This work provides a mild, efficient, and scalable alternative to traditional methods for the synthesis of tetrasubstituted enantiopure piperazines, with potential applications in the preparation of chiral ligands.

## Introduction

**Scheme 1:**
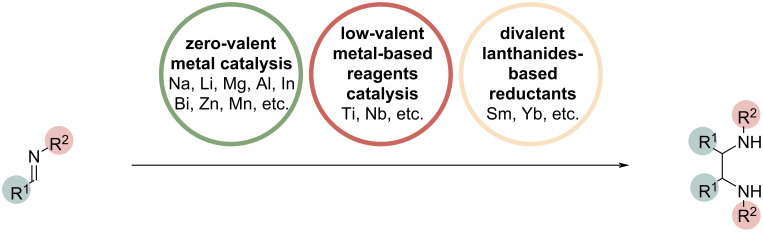
Synthesis of vicinal diamines via imino-pinacol coupling in the presence of metal-based reductants.

Vicinal diamines represent a highly valuable class of compounds that, over the past decades, have found widespread application in natural products, agrochemicals, and pharmacologically active compounds. Enantiomerically pure 1,2-diamines and their derivatives are also increasingly used in stereoselective synthesis, particularly as chiral auxiliaries or as ligands for metal complexes in asymmetric catalysis [1]. Metal-based reductants represent the most established approach to imino-pinacol coupling (Scheme 1), with zero-valent metals traditionally employed as reductants and various strategies extensively explored [2]. The use of alkali metals [3–5], as lithium and sodium, and alkaline earth metals [6], as magnesium, are characteristic of the earliest versions of this transformation. Several metals from the p- and the d-blocks of the periodic table, such as aluminum [7], indium [8], bismuth [7], zinc [9–17], and manganese [18–19], were later studied. The use of zinc, in particular, has attracted significant attention in this context due to its flexibility, efficiency, and practical applicability. Since the late 1980s, many research groups have investigated the use of in situ-generated low-valent titanium [20–25] and niobium [26] reagents to promote the pinacol-type coupling of imines. Among the metal-based reducing agents explored for this transformation, divalent lanthanide-based reductants derived from samarium and ytterbium have received significant attention since the early 90’s [27–34].

**Scheme 2:**
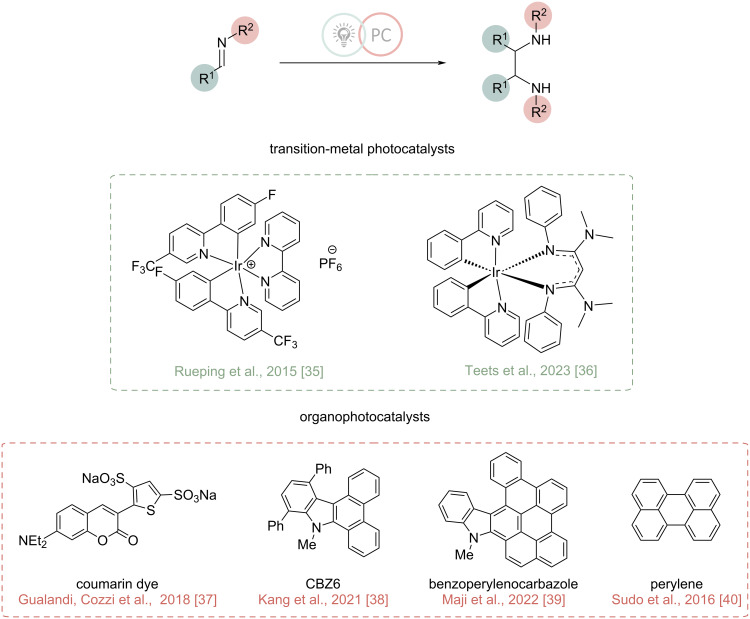
Light-promoted imino-pinacol coupling for the synthesis of vicinal diamines.

Although traditional well-established procedures for the imino-pinacol coupling reaction are efficient, they often require more than stoichiometric amounts of metal reductants and produce significant quantities of metal waste, which in some cases can present considerable environmental and safety concerns. To mitigate this issue, more sustainable approaches, such as photochemical and electrochemical methods, have been explored. Over the past two decades a variety of light-promoted imino-pinacol coupling reactions have been developed, involving the use of catalytic transition-metal complexes [35–36], organic dyes [37–39], and polyaromatic compounds [40–41] as photocatalysts (Scheme 2). The combination of photoredox catalysis with imine activation enabled the reductive coupling of imines under mild reaction conditions, providing direct access to benzyl and aryl vicinal diamines with good to excellent yields.

**Scheme 3:**
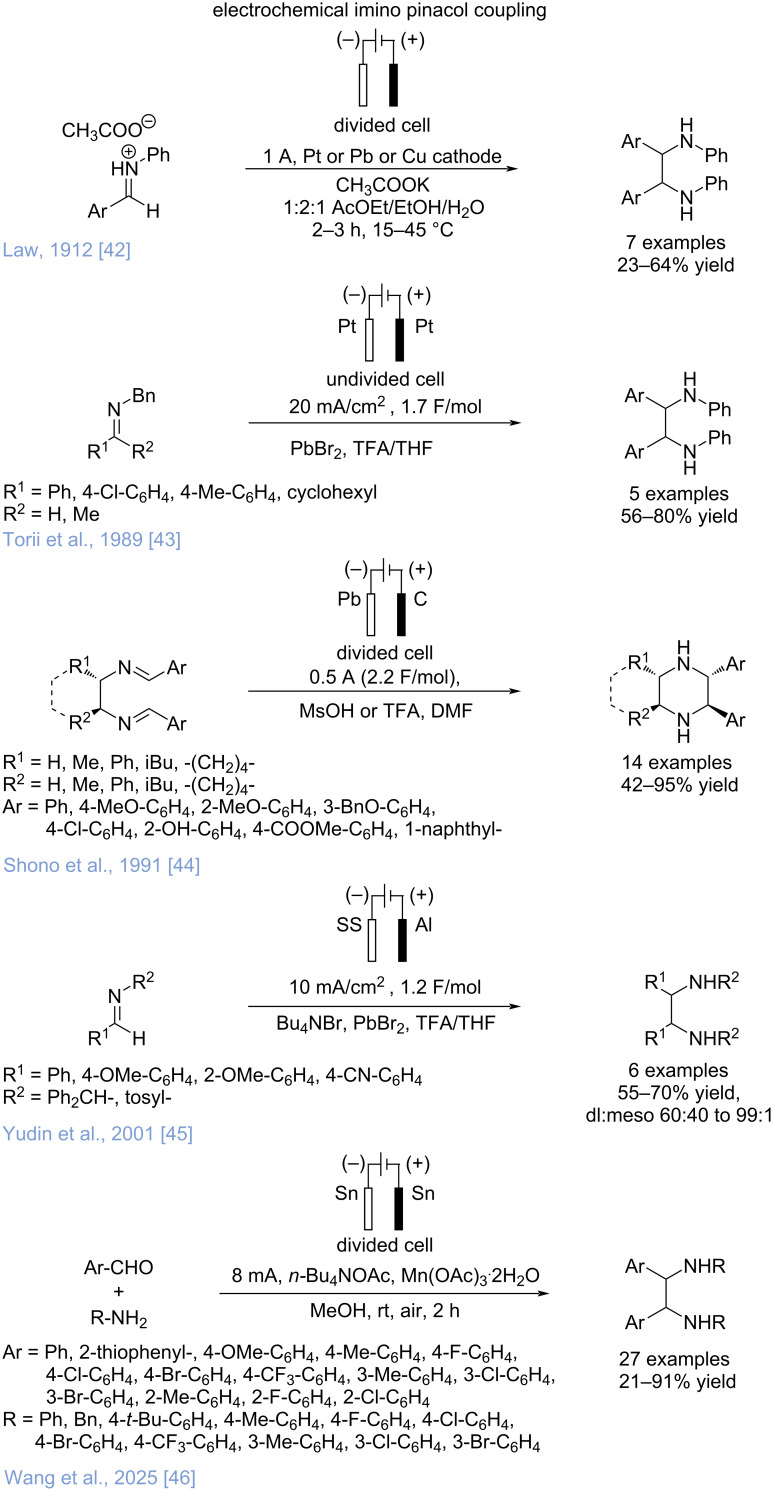
Historical perspective on electrochemical imino-coupling protocols.

Organic electrochemistry represents an attractive and sustainable alternative; however, unfortunately, the electrochemical application is limited to only a few examples (Scheme 3). The electroreductive coupling of imines was first reported in the early 20th century by Law [42]. However, this initial method led to the formation of vicinal diamines with low to moderate yields and required geometrically complex divided cells. In 1989, a more efficient procedure was introduced by Torii et al. [43], who simplified the process by using PbBr_2_, TFA and THF, in a beaker-type undivided cell equipped with two platinum electrodes. This approach afforded vicinal diamines for different *N*-benzyl benzaldimines in moderate to good yields. In 1991, Shono’s research group described the electroreductive intramolecular coupling of aromatic diimines, carried out in DMF in the presence of methanesulfonic acid in a divided cell equipped with a lead cathode, a carbon rod anode, and a ceramic diaphragm [44]. This method was found to be effective for synthesizing *trans*-2,3-diarylpiperazines through the intramolecular reductive coupling of 1,2-diimines and seven- and eight-membered heterocycles which were obtained in moderate to good yields via the cyclization of 1,3- and 1,4-diimines.

Later, in 2001, Yudin and co-workers described the parallel reductive coupling of aldimines using a spatially addressable electrosynthesis platform, which employed a stainless steel cathode, a sacrificial aluminum anode and a procedure adapted from Torii's earlier work including the use of PbBr_2_, TFA, and Bu_4_NBr [45]. Under these conditions, parallel electrosynthesis allowed the synthesis of up to 16 vicinal diamines in good yields in 30 minutes. To the best of our knowledge, the most recent example of electroreductive coupling of imines was reported in 2025 by Wang’s research group [46], who described a new electrochemical procedure to provide vicinal diamines involving the use of Sn electrodes as both anode and cathode in a divided cell, Mn(OAc)_3_·2H_2_O as additive and *n*-Bu_4_NOAc as electrolyte. Under these conditions, using aldehydes and amines as starting materials to form the imines in situ resulted in a wide range of diamines, obtained in moderate to good yields.

**Scheme 4:**
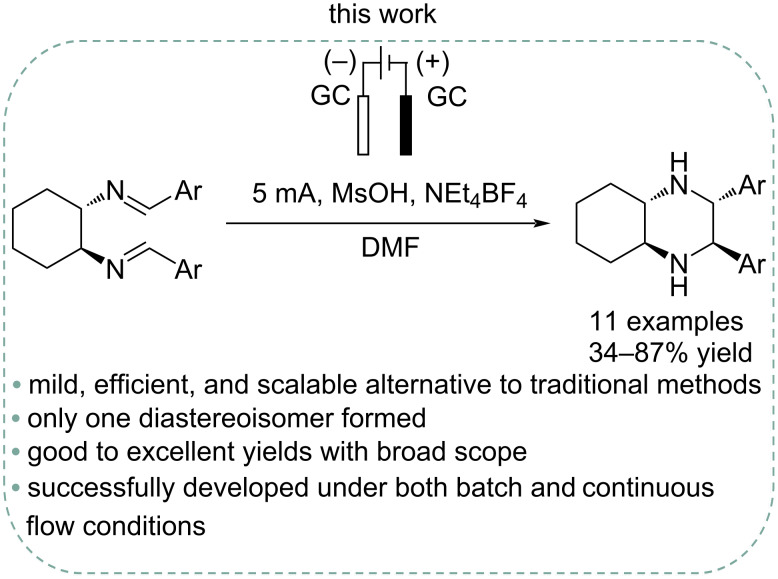
Stereoselective electroreductive intramolecular imino-pinacol reaction.

Inspired by these works, we aimed to investigate and optimize the stereoselective electrochemical intramolecular imino-pinacol coupling reaction under both batch and continuous flow conditions as a direct approach to enantiopure scaffolds that are commonly used as chiral ligands. In this context we report the development of a more sustainable method – which avoids the use of lead bromide or lead electrodes – employing an undivided cell with two glassy carbon electrodes for the electroreductive intramolecular coupling of aromatic diimines (Scheme 4). This methodology has been successfully applied to the synthesis of enantiopure tetrasubstituted piperazines, featuring both electron-withdrawing and electron-donating groups on the aromatic rings.

## Results and Discussion

The stereoselective electroreductive intramolecular coupling of chiral diimines of aromatic aldehydes with *trans-*1,2-diaminocyclohexane was initially investigated under traditional batch conditions through a series of screening experiments designed to find the optimal reaction conditions.

Diimine **1a** was selected as model substrate for the intramolecular coupling reaction and a screening of reaction parameters such as solvents, electrodes materials, electrolytes, total charges, and concentrations of the reaction mixture was performed. All optimization studies were carried out using 0.5 mmol of **1a** in the presence of methanesulfonic acid in an undivided cell (5 mL volume) equipped with two electrodes under galvanostatic conditions (constant current 5 mA, 2.5 mA/cm^2^) and the results are summarized in Table 1.

**Table 1:** Screening of the imino-pinacol reaction conditions.

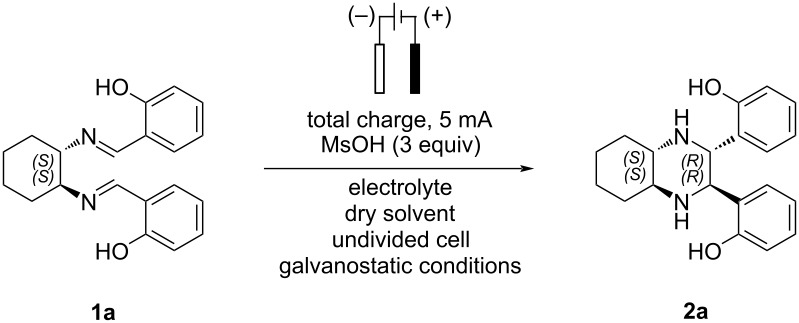								

Entry	Working (cathode)	Counter (anode)	Electrolyte	Electrolyte equivalents	Total charge (F/mol)	Solvent	Substrate concentration (M)	Yield **2a** (%)^a^

1	Pt	Pt	NEt_4_BF_4_	2.6	2.2	MeCN	0.125	51
2	Pt	Pt	NEt_4_BF_4_	2.6	2.2	DMF	0.125	68
3^b^	Pt	Pt	NEt_4_BF_4_	2.6	2.2	DMF	0.125	–
4	Pt	Gr	NEt_4_BF_4_	2.6	2.2	DMF	0.125	64
5	Pt	Zn	NEt_4_BF_4_	2.6	2.2	DMF	0.125	64
6	Pt	Zn	NEt_4_BF_4_	2.6	2.5	DMF	0.125	61
7	Pt	Zn	NEt_4_BF_4_	2.6	2.5	DMF	0.250	64
8	SS	SS	NEt_4_BF_4_	2.6	2.2	DMF	0.125	47
**9**	**GC**	**GC**	**NEt** **_4_** **BF** **_4_**	**2.6**	**2.2**	**DMF**	**0.125**	**85**
10	GC	GC	NEt_4_BF_4_	1.3	2.2	DMF	0.125	50
11	GC	GC	NBu_4_BF_4_	2.6	2.2	DMF	0.125	72

^a^Isolated yields. Reactions were performed on a 0.5 mmol scale. ^b^Reaction performed without MsOH.

Using platinum electrodes and NEt₄BF₄ as electrolyte, the tetrasubstituted piperazine **2a** was obtained as a single stereoisomer in 51% yield in CH_3_CN, which increased to 68% when DMF was used (Table 1, entry 2). The replacement of the platinum anode with graphite or zinc (Table 1, entries 4 and 5) had no significant effect on the yield. Similarly, increasing the total charge from 2.2 to 2.5 F/mol (Table 1, entries 6 and 7) resulted in only marginal changes. Doubling the substrate concentration (Table 1, entry 7) also failed to provide a significant advantage in terms of yield, leading to the formation of the desired product in 64% yield.

The replacement of both working cathode and counter anode to stainless steel electrodes caused a decrement of the yield to 47% (Table 1, entry 8), while with two glassy carbon electrodes desired product **2a** was isolated in 85% yield (Table 1, entry 9).

Reducing the electrolyte loading from 2.6 to 1.3 equivalents significantly affected the reaction outcome, leading to a noticeable decrease in the yield suggesting that sufficient ionic conductivity is crucial to achieve efficient conversion (Table 1, entry 10).

Lastly, replacing tetraethylammonium tetrafluoroborate with tetrabutylammonium tetrafluoroborate resulted in a negligible reduction of the chemical efficiency (Table 1, entry 9 vs 11).

As demonstrated by Shono and co-workers [44], the presence of a strong protic acid, such as methanesulfonic acid, MsOH, is essential to promote the intramolecular coupling. A control experiment revealed that when the electroreduction of **1a** was performed in the absence of MsOH (Table 1, entry 3), no formation of the desired product was observed and the starting diimine **1a** was quantitatively recovered.

**Scheme 5:**
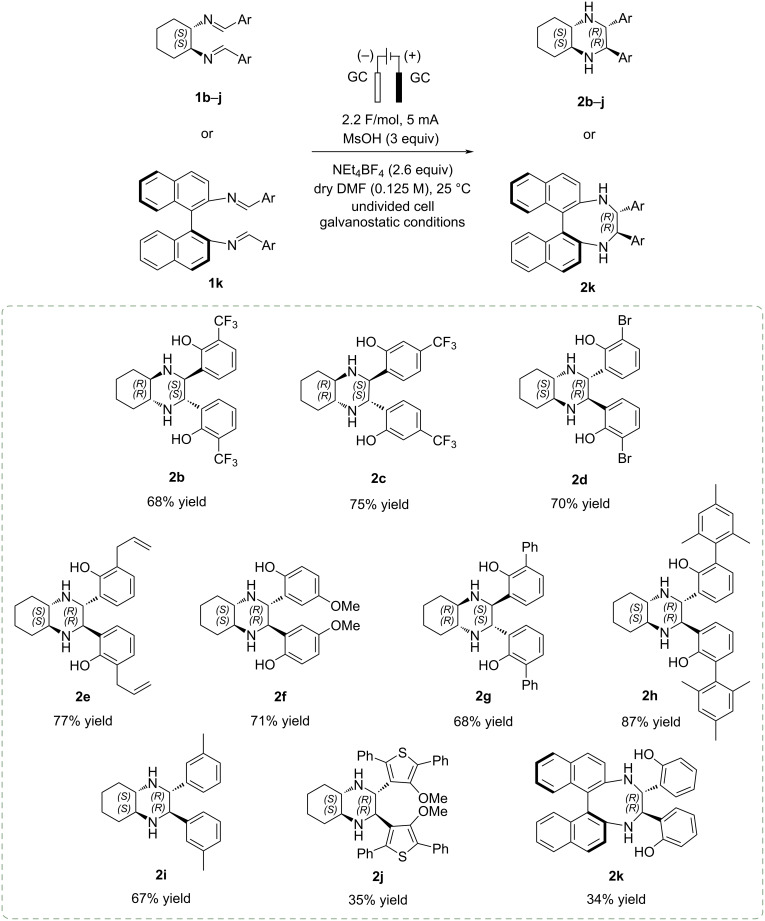
Scope of the imino-pinacol coupling reaction. Reaction conditions: GC electrodes, NEt_4_BF_4_ (2.6 equiv), MsOH (3 equiv), constant current 5 mA (2.5 mA/cm^2^), total charge 2.2 F/mol, dry DMF (0.125 M). Yields reported are isolated yields. Reactions were performed on a 0.5 mmol scale.

The optimized reaction conditions, consisting in the use of GC electrodes, NEt_4_BF_4_ (2.6 equiv) as electrolyte, MsOH (3 equiv) as additive, constant current of 5 mA (2.5 mA/cm^2^), total charge of 2.2 F/mol, dry DMF as solvent (0.125 M), were then applied to evaluate the scope of the reaction. Under these conditions a small library of chiral enantiopure tetrasubstituted piperazines **2b**–**j** bearing both electron-withdrawing and electron-donating substituents, was synthetized. The results are summarized in Scheme 5.

The chiral tetrasubstituted piperazines **2b**–**j** were obtained as a single, enantiopure diastereoisomer from diimines **1b**–**j**, prepared from the enantiopure *trans*-1,2-diaminocyclohexane and aromatic aldehydes, in good to excellent yields. Substrates bearing electron-withdrawing groups such as trifluoromethyl (**2b**,**c**) and bromine (**2d**), as well as electron-donating groups like methoxy (**2f**), aryl/alkyl (**2h**,**i**), and alkenyl substituents (**2e**), were well tolerated. Notably, the sterically hindered derivative **2h** was isolated with the highest yield of 87% indicating that bulky *ortho*-substituents on the aromatic rings do not influence the electrochemical cyclization process.

In contrast, more complex and extended heterocyclic electronrich π-systems such as compound **2j** was obtained in 35% yield only, presumably as a result of electronic factors affecting the efficiency of the initial radical formation [47].

To further extend the scope of this methodology, the *trans*-1,2-diaminocyclohexane was replaced with (*S*)-1,1′-binaphthyl-2,2′-diamine. Under the optimized conditions, the corresponding cyclic product **2k** was obtained in 34% yield, consistent with previous observations reporting reduced efficiency as the ring size increases [44].

**Figure 1:**
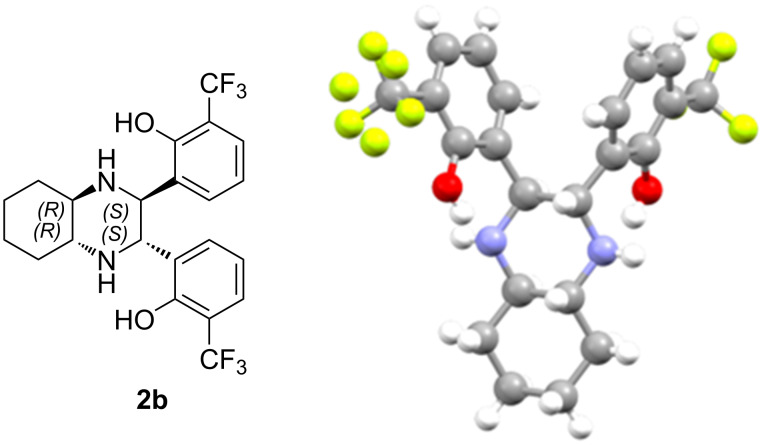
X-ray determined structure of chiral piperazine **2b**.

To confirm the absolute configuration of the piperazine formed, a pure sample of compound **2b** derived from (1*R*,2*R*)-*trans*-diaminocyclohexane was crystallized from a chloroform solution resulting in the formation of white crystals suitable for single crystal X-ray diffraction studies. The absolute configuration of the product was unambiguously determined, by XRD analysis, to be (2*S*,3*S*,4a*R*,8a*R*)-**2b** as shown in Figure 1.

Following the promising results obtained from the batch approach, the electroreductive coupling was investigated under continuous flow conditions [48–50], with the aim to improve process efficiency and in view of a possible scale up of the reaction [51–52]. Flow experiments were performed using the commercially available pre-configured Syrris-Asia electrochemical flow module, a user-friendly solution for continuous flow electrochemical synthesis which is part of the Asia platform [53].

**Scheme 6:**
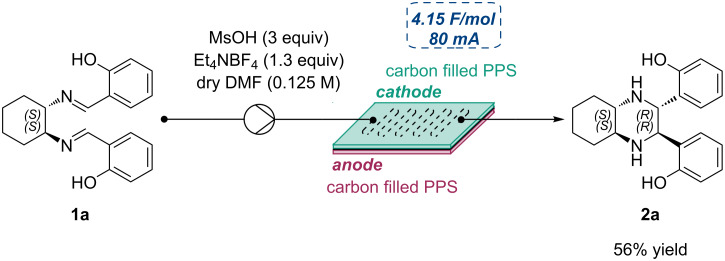
Continuous flow synthesis of piperazine **2a**. The yield was determined by ^1^H NMR spectroscopy using 1,3,5-trimethoxybenzene as internal standard. Reactions were performed on a 0.1 mmol scale.

A comprehensive optimization of the flow reaction conditions was carried out using the model substrate **1a** systematically varying key parameters such as the working electrodes, the current intensity, the total charge, the flow rate, and the equivalents of the employed electrolyte. Following this in-depth screening, optimal conditions were identified: Carbon filled PPS electrodes, a constant current of 80 mA, a total charge of 4.15 F/mol, a flow rate of 96 μL/min, and 1.3 equivalents of NEt_4_BF_4_ as electrolyte. Under these optimized conditions, the desired tetrasubstituted piperazine **2a** was obtained as single stereoisomer in 56% yield with a residence time of 2.34 minutes (Scheme 6).

Although the yield appears lower compared to the batch process, it is important to highlight that the reaction time has been reduced by nearly a factor of 150, resulting in a substantial increase in terms of productivity, which is 6.2 times higher than the batch protocol. Moreover, the significantly shorter residence time in the flow setup led to an improved space-time yield (STY), a key metric for comparing reactors of different volumes. Under these conditions, the STY of the flow process was 112.2 times higher than that achieved in the batch reactor (Table 2).

**Table 2:** Comparison of productivity and space-time yield of batch and flow processes for the synthesis of piperazine **2a**.

Entry	Method	Productivity^a^ (mmol/h)	Productivity rel. factor	STY^b^ (mmol/mL*h)	STY rel. factor

1	batch	0.065	1	0.016	1
2	flow	0.403	6.2	1.795	112.2

^a^Productivity*:* moles of product divided by the collection time required to collect the product obtained by the reaction of 0.5 mmol of diimine **1a**. ^b^Space-time yield: moles of product in the reactor, divided by residence time and reactor volume (for details on calculations see Supporting Information File 1).

The scalability of the reaction was further demonstrated by performing the electroreduction of diimine **1d** on a 2.5 mmol scale under flow conditions. Compound **2d** was obtained in 52% isolated yield achieving productivity and STY values that were 10.4 and 184.7 times higher, respectively, compared to those obtained in the batch reaction (Table 3).

**Table 3:** Comparison of productivity and space-time yield of batch and flow processes for the synthesis of piperazine **2d**.

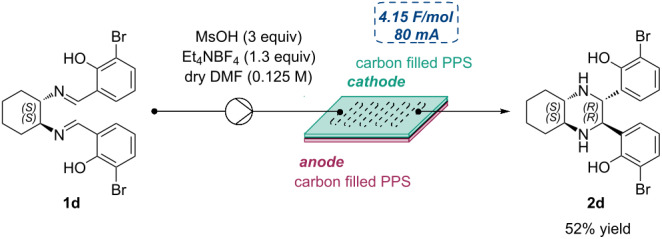					

Entry	Method	Productivity^a^ (mmol/h)	Productivity rel. factor	STY ^b^ (mmol/mL*h)	STY rel. factor

1	batch	0.036	1	0.009	1
2	flow	0.374	10.4	1.662	184.7

^a^Productivity: moles of product divided by the collection time required to collect the product obtained by the reaction of 0.5 mmol of diimine **1d**. ^b^Space-time yield: moles of product in the reactor, divided by residence time and reactor volume (for details on calculations please see Supporting Information File 1).

**Scheme 7:**
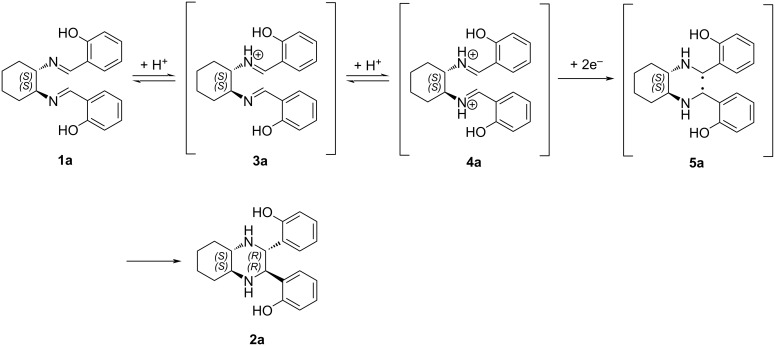
Proposed reaction mechanism.

To gain a deeper understanding of the mechanism behind the observed reaction, a plausible reaction pathway is proposed, as illustrated in Scheme 7. The process presumably involves the activation of the diimine substrate **1a** by methanesulfonic acid, which acts as a strong Brønsted acid to selectively protonate the imine nitrogen atoms. This protonation step increases the electrophilicity of the adjacent imine carbons by inductive effect, leading to the formation of a highly reactive diiminium intermediate **4a**. When formed, compound **4a** is electrochemically reduced to give the carbon-centered diradical intermediate **5a** and the spatial proximity of these two radical centers allows a rapid intramolecular radical–radical coupling resulting in the formation of the desired piperazine **2a**. The feasibility of this mechanism is supported by literature precedents involving electroreduction of activated imines and diiminium species [19]. Furthermore, control experiments conducted in the absence of methanesulfonic acid (Table 1, entry 3) resulted in no observable product formation, highlighting the acid activation in driving this transformation.

**Scheme 8:**
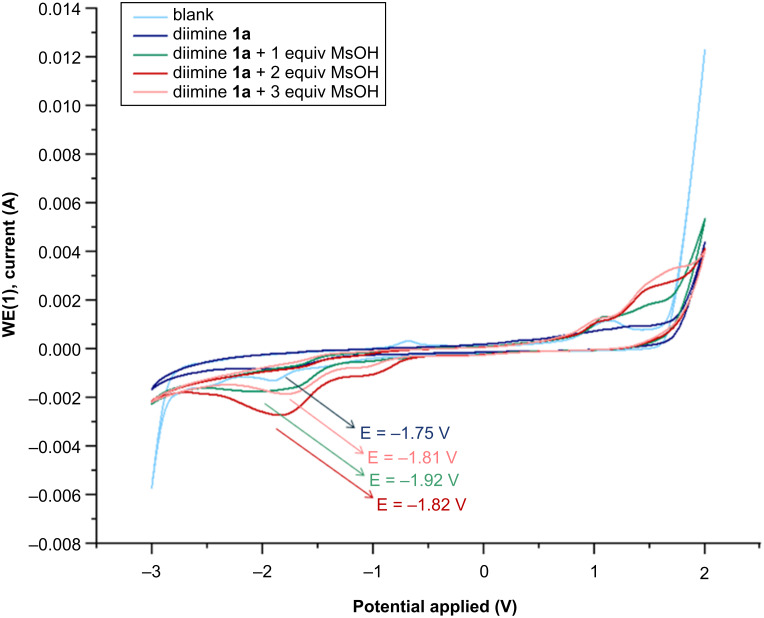
Cyclic voltammetry investigation. Cyclic voltammetry of a 0.325 M solution of Et_4_NBF_4_ in DMF (light-blue line). Cyclic voltammetry of diimine **1a** (10 mM) recorded in a 0.325 M solution of Et_4_NBF_4_ in DMF (dark-blue line). Cyclic voltammetry of diimine **1a** (10 mM) in presence of 1 equiv of methanesulfonic acid (10 mM) recorded in a 0.325 M solution of Et_4_NBF_4_ in DMF (green line). Cyclic voltammetry of diimine **1a** (10 mM) in presence of 2 equiv of methanesulfonic acid (20 mM) recorded in a 0.325 M solution of Et_4_NBF_4_ in DMF (dark-red line). Cyclic voltammetry of diimine **1a** (10 mM) in presence of 3 equiv of methanesulfonic acid (30 mM) recorded in a 0.325 M solution of Et_4_NBF_4_ in DMF (light-red line). Glassy carbon as working, glassy carbon as counter, and Ag/AgCl as reference electrodes with 0.1 V/s as scan rate.

Cyclic voltammetry measurements were also carried out in order to evaluate the electrochemical redox properties of the species involved in the process and to provide evidence for the behavior of the monoprotonated and the bisprotonated diimines, **3a** and **4a** (Scheme 8). The cyclic voltammogram of the starting diimine **1a** shows one distinct reductive peak at −1.75 V. Upon addition of one equivalent of MsOH, monoprotonated species **3a** is formed, and a shift of the reduction peak to −1.92 V was observed. Subsequent addition of a second equivalent of acid leads to the formation of the bisprotonated species **4a**, and as consequence, a shift of the reduction peak to −1.82 V was observed. However, the addition of three equivalents of methanesulfonic acid (corresponding to the typical reaction conditions) results in a decrease in the intensity of the reduction peak at −1.81 V. This observation is presumably associated with the initiation of the SET reduction process, which leads to the consumption of the diiminium salt **4a** and to the formation of the diradical intermediate **5a**.

## Conclusion

In conclusion, we have successfully developed a simple and mild electroreductive, stereoselective intramolecular coupling of aromatic diimines, performed under batch and flow conditions. The reaction provided good to excellent yields across a wide range of substrates with both electron-withdrawing and electron-donating groups being well tolerated. As a result, a series of chiral enantiopure tetrasubstituted piperazines was efficiently synthetized. Significantly higher productivities and space-time yields were achieved under continuous conditions compared to the batch process. Furthermore, the scalability of the reaction was successfully demonstrated, highlighting its potential for larger-scale applications.

## Experimental

### General procedure in-batch electrochemical imino-pinacol coupling

In a flame-dried, undivided cell equipped with two GC electrodes and a stirring bar, diimine (0.5 mmol, 1 equiv) and NEt_4_BF_4_ (1.3 mmol, 2.6 equiv) were added, followed by two vacuum–nitrogen cycles. Under nitrogen atmosphere, dry DMF (0.125 M) and methanesulfonic acid (1.5 mmol, 3 equiv) were then added to the electrochemical cell. The reaction mixture was degassed by bubbling with argon for 20 minutes under vigorous stirring. The undivided cell was then connected to the Autolab power supply and stirred at 25 °C under galvanostatic conditions at 5 mA (2.5 mA/cm^2^) until a total charge of 2.2 F/mol was delivered. The reaction mixture was poured into a beaker with 10 mL of distilled water, and a saturated solution of NaHCO_3_ was added to adjust the pH to ≈7. 10 mL of ethyl acetate were then added, and the two phases were separated. The aqueous phase was then extracted with CH_2_Cl_2_ (3 × 10 mL). The combined organic layers were dried over Na_2_SO_4_, filtered and concentrated under vacuum. The reaction crude was purified by flash column chromatography on silica gel (*n*-hexane/ethyl acetate 8:2) to give the desired pure product.

### General procedure for in-flow electrochemical imino-pinacol coupling

Diimine (1 equiv) and NEt_4_BF_4_ (1.3 equiv) were added in a flame-dried Erlenmeyer flask with a stirring bar, followed by two vacuum–nitrogen cycles. Under nitrogen atmosphere, dry DMF (0.125 M) and methanesulfonic acid (3 equiv) were then added to the reaction mixture, which was degassed by bubbling with argon for 20 minutes under vigorous stirring. In-flow experiments were performed using the Asia (Syrris) modular system with two carbon filled PPS electrodes, a constant current of 80 mA, a flow rate of 96 μL/min, a residence time of 2.34 minutes, and 1.3 equivalents of NEt_4_BF_4_. The experiments were conducted until a total charge of 4.15 F/mol was delivered. The reaction mixture was then poured into a beaker with distilled water, and a saturated solution of NaHCO_3_ was added to adjust the pH to ≈7. Ethyl acetate was added, and the two phases were separated. The aqueous phase was then extracted three times with CH_2_Cl_2_. The combined organic layers were dried over Na_2_SO_4_, filtered and concentrated under vacuum. Yields were calculated on the reaction crude by ^1^H NMR spectroscopy using 1,3,5-trimethoxybenzene as internal standard. In the case of the large-scale reaction the crude was purified through flash column chromatography on silica gel to give the isolated pure product.

## Supporting Information

File 1 Synthetic procedures and physical data for the new compounds, copies of ^1^H and ^13^C NMR spectra of the prepared compounds.

## Data Availability

Data generated and analyzed during this study is available from the corresponding author upon reasonable request.
