# Supplementary material for: Stereoselective electrochemical intramolecular imino-pinacol reaction: a straightforward entry to enantiopure piperazines
Source: Beilstein J Org Chem. 2025 Sep 12;21:1897–908. doi: 10.3762/bjoc.21.147 (PMC12434927; doi:10.3762/bjoc.21.147)
Supplement: File 1 — Synthetic procedures and physical data for the new compounds, copies of 1H and 13C NMR spectra of the prepared compounds. [file Beilstein_J_Org_Chem-21-1897-s001.pdf]

BEILSTEIN JOURNAL OF ORGANIC CHEMISTRY

Supporting Information

for

Stereoselective electrochemical intramolecular imino-pinacol reaction: a straightforward entry to enantiopure piperazines

Margherita Gazzotti, Fabrizio Medici, Valerio Chiroli, Laura Raimondi, Sergio Rossi and Maurizio Benaglia

Beilstein J. Org. Chem. **2025,** *21,* 1897–1908. doi:10.3762/bjoc.21.147

Synthetic procedures and physical data for the new compounds, copies of ^{1}H and ^{13}C NMR spectra of the prepared compounds

License and Terms: This is a supporting information file under the terms of the Creative Commons Attribution License (https://creativecommons.org/licenses/by/4.0). Please note that the reuse, redistribution and reproduction in particular requires that the author(s) and source are credited and that individual graphics may be subject to special legal provisions.
The license is subject to the *Beilstein Journal of Organic Chemistry* terms and conditions: (https://www.beilstein-journals.org/bjoc/terms)

Table of contents

1. General methods S2
2. Substrate synthesis S4
   2.1. Aldehydes synthesis S4
      2-Hydroxy-3-(trifluoromethyl)benzaldehyde (**5b**) S4
      2-Hydroxy-4-(trifluoromethyl)benzaldehyde (**5c**) S5
      3-Allyl-2-hydroxybenzaldehyde (**5e**) S6
      2-Hydroxy-[1,1'-biphenyl]-3-carbaldehyde (**5g**) S7
      2-Hydroxy-2',4',6'-trimethyl-[1,1'-biphenyl]-3-carbaldehyde (**5h**) S8
      4-Methoxy-2,5-diphenylthiophene-3-carbaldehyde (**5j**) S9
   2.2. Diimines synthesis S11
      General procedure A [7] S11
      General procedure B [8] S11
3. Electrochemical imino-pinacol coupling in batch S12
   3.1. Reaction conditions optimization S12
   General procedure S12
   3.2. Reaction scope S14
   General procedure S14
4. In-flow electrochemical imino-pinacol coupling S18
   4.1. Reaction conditions optimization S18
   General procedure S18
   4.2. Large-scale reaction S20
6. NMR spectra and HRMS S25
7. Crystallographic data S46
8. References S50

1. General methods

Dry solvents: Dry solvents were purchased and stored under nitrogen over molecular sieves (bottles with crown caps).

Chemicals: compounds **5a**, **5d**, **5f** and **5i** were purchased from standard commercial suppliers and, when necessary, purified prior to use.

Chromatographic purification: Purification of products was performed by column chromatography with flash technique (according to the Still method) using as stationary phase silica gel 230–400 mesh purchase from MERCK.

Thin-layer chromatography (TLC): Reactions and chromatographic purifications were monitored by analytical thin-layer chromatography (TLC) using silica gel 60 F254 pre-coated glass plates (0.25 mm thickness) and visualized using UV light (365 nm).

NMR spectra: ^{1}H NMR spectra were recorded on spectrometers operating at 300 MHz (Bruker Avance 300), at 400 MHz (Bruker NEO400 Avance) or at 600 MHz (Bruker NEO600 Avance). The chemical shifts are reported in ppm (δ), with the solvent reference relative to tetramethyl silane (TMS) employed as the internal standard ($CDCl_3$ δ = 7.26 ppm, MeOD δ = 3.31 ppm, DMSO δ = 2.50 ppm). ^{13}C NMR spectra were recorded on a 300 MHz, a 400 MHz or a 600 MHz spectrometers (Bruker Avance 300, Bruker NEO400 Avance or Bruker NEO600 Avance) operating at 75, 101 or 151 MHz, with complete proton decoupling. Carbon chemical shifts are reported in ppm (δ) relative to TMS with the respective solvent resonance as the internal standard ($CDCl_3$ δ = 77.16 ppm, MeOD δ = 49.00 ppm, DMSO δ = 39.52 ppm). ^{19}F NMR spectra were recorded on 300 MHz spectrometers (Bruker Avance 300) operating at 282.1 MHz; fluorine chemical shifts are reported in ppm (δ) relative to $CFCl_3$ with the respective solvent resonance as the internal standard ($CFCl_3$: δ = 77.0 ppm). ^{1}H NMR, ^{13}C NMR, ^{31}P NMR and ^{19}F NMR spectra of all the compounds synthesized were recorded in $CDCl_3$, MeOD or DMSO.

Mass spectra: High-resolution mass spectra (HRMS) were acquired using a Q-TOF Synapt G2-Si/HDMS 8K instrument available at the MS facility of the Unitech COSPECT at the University of Milan.

Optical rotations: Optical rotations were obtained on a JASCO P-1030 polarimeter (Series: A014060839) at 589 nm using a 1 mL cell, with a length of 1 dm.

Metrohm Autolab potentiostat: Electrochemical imino-pinacol coupling reactions in batch were performed using a Metrohm Autolab M204 potentiostat and a two-electrode configuration. Platinum, graphite, zinc, stainless steel and glassy carbon electrodes from IKA were used. All reactions were performed in a 10 mL electrochemical cell (IKA) in Ar atmosphere and at 25 °C.

Cyclic voltammetry: Cyclic voltammograms were recorded using a Metrohm Autolab M204 potentiostat. The measurements were performed under an argon atmosphere using two glassy carbon electrodes as working and counter electrodes and Ag/AgCl as reference electrode. All CV analyses were recorded in a 0.325 M solution of tetra-*n*-butylammonium tetrafluoroborate in dry DMF with 0.1 V/s scan rate.

ASIA Syrris: Electrochemical imino-pinacol coupling reactions in flow were performed using an ASIA modular system from Syrris. The system was composed by a syringe pump module connected by PFA (0.5 mm ID) tubes to a FLUX module equipped with an electrochemical cell (volume 225 µL). The experiments were performed with platinum or carbon filled PPS electrodes, purchased from Syrris, at 25 °C.

2. Substrate synthesis

2.1. Aldehydes synthesis

2-Hydroxy-3-(trifluoromethyl)benzaldehyde (5b)

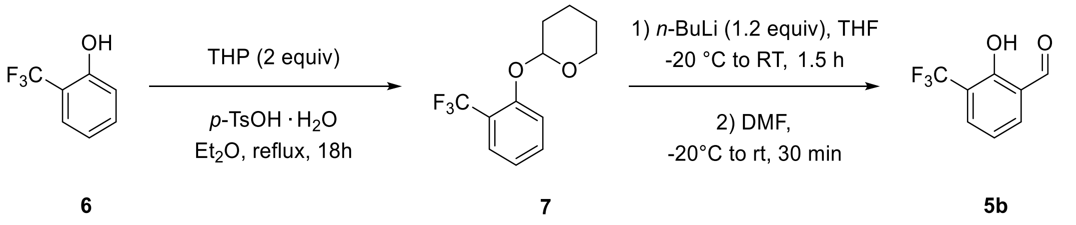

p-Toluenesulfonic acid monohydrate (38 mg, 0.2 mmol, 0.02 equiv) was added to a flask containing a solution of 2-(trifluoromethyl)phenol (**6**, 1.62 g, 10.0 mmol, 1 equiv) and 3,4-dihydro-2*H*-pyran (THP, 3.65 mL, 40.0 mmol, 2 equiv) in Et_2O (0.5 M). The mixture was then refluxed for 18 hours. After cooling to room temperature, 50 mL of ethyl acetate were added. The solution was then washed with sat. aq. $NaHCO_3$ (3 × 10 mL), the organic phase was dried over $MgSO_4$, filtered and concentrated under reduced pressure (evaporation at low temperature due to the volatility of the product). The crude material was purified by silica gel chromatography (pentane/ethyl acetate 97:3) to obtain 2-(2-(trifluoromethyl)phenoxy)tetrahydro-2*H*-pyran (**7**) as a colourless liquid (85% yield).

^{1}H NMR ($CDCl_3$, 300 MHz) δ 7.57 (d, *J* = 7.57 Hz, 1H), 7.46 (t, *J* = 7.92 Hz, 1H), 7.26 (d, *J* = 7.91 Hz, 1H), 7.02 (t, *J* = 7.58 Hz, 1H), 5.57 (bs, 1H), 3.86 (td, *J* = 11.10, 11.04, 2.88 Hz, 1H), 3.64-3.60 (m, 1H), 2.07-1.59 (m, 7H). Data in accordance with the literature [1].

To a flame-dried flask under nitrogen atmosphere 2-(2-(trifluoromethyl)phenoxy)tetrahydro-2*H*-pyran (**7**, 1.0 g, 4.06 mmol, 1 equiv) and distilled THF (0.26 M) were added. The mixture was cooled to −20 °C and *n*-BuLi (1.6 M in THF, 4.2 mL, 1.2 equiv) was added dropwise. The reaction was stirred at −20 °C for 30 minutes and then at room temperature for 1 hour. The mixture was cooled back to −20 °C before the addition of DMF (646.0 μL, 7.71 mmol, 1.9 equiv), which was added dropwise. The solution was warmed to room temperature for 30 minutes and then it was quenched with aqueous HCl (2.0 M, 7 mL). The mixture was diluted with Et_2O (60 mL), washed with water (3 × 20 mL) and the organic phase was dried over $MgSO_4$ and concentrated under reduced pressure (evaporation at low temperature due to the volatility of the product). The crude was purified by silica gel column chromatography (pentane/ethyl acetate 90:10), to obtain 2-hydroxy-3-(trifluoromethyl)benzaldehyde (**5b**) as a yellow solid (75% yield).

^{1}H NMR ($CDCl_3$, 300 MHz) δ 11.73 (s,1H), 9.96 (s, 1H), 7.80 (dd, *J* = 17.58, 7.79 Hz, 2H), 7.12 (t, *J* = 7.73 Hz, 1H). The data are in agreement with those reported in the literature [1].

2-Hydroxy-4-(trifluoromethyl)benzaldehyde (5c)

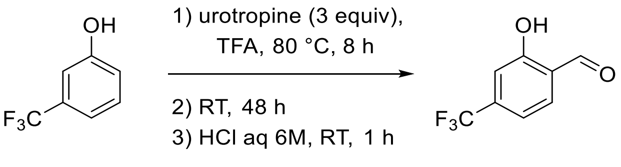

To a solution of 3-trifluoromethylphenol (**8**, 3 mL, 25 mmol, 1 equiv) in trifluoroacetic acid (25 mL), urotropine (10.5 g, 75 mmol, 3 equiv) was added portion-wise. The mixture was stirred at 80 °C for 8 hours. After that, it was allowed to cool to room temperature and stirred for additional 48 hours. HCl 6 M was then added (25 mL) and the mixture stirred for 1 hour. The pH was then adjusted to 5 by adding 10% NaOH aq. solution and the mixture was subsequently extracted with Et_2O (3 × 25 mL). The collected organic phases were dried over $MgSO_4$ and concentrated under reduced pressure (evaporation at low temperature due to the volatility of the product). The crude was purified by silica gel column chromatography (pentane/Et_2O 90:10), obtaining 2-hydroxy-4-(trifluoro-methyl)benzaldehyde (**5c**) as a pale-yellow liquid (80% yield).

^1H NMR (300 MHz, $CDCl_3$) δ 11.08 (s, 1H), 9.99 (s, 1H), 7.72 (dd, *J* = 8.4, 1.0 Hz, 1H), 7.28 (dd, *J* = 4.5, 1.5 Hz, 2H). The data are in agreement with those reported in the literature [1].

3-Allyl-2-hydroxybenzaldehyde (5e)

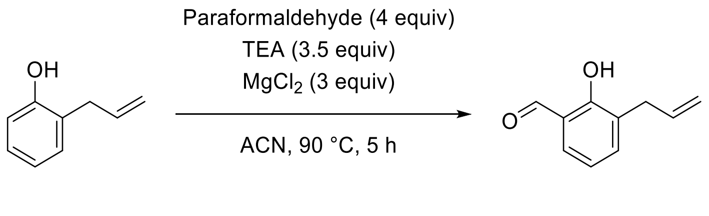

In a flame-dried sealed vial containing a suspension of anhydrous $MgCl_2$ (285.6 mg, 3.0 mmol, 3 equiv) in dry acetonitrile (0.2 M), paraformaldehyde (120.1 mg, 4.0 mmol, 4 equiv), 2-allylphenol (**9**, 130.5 μL, 1 mmol, 1 equiv) and triethylamine (487.8 μL, 3.5 mmol, 3.5 equiv) were added in this order. The reaction mixture was refluxed under nitrogen atmosphere for 5 hours. After the complete consumption of the phenol (monitored by TLC, eluent *n*-hexane/ethyl acetate 8:2), the mixture was cooled to room temperature, quenched with aqueous HCl 6 M until pH 2, diluted with water (25 mL) and then extracted with Et_2O (4 × 20 mL). The collected organic phases were dried over $MgSO_4$, filtered and dried under reduced pressure. The residue was purified by column chromatography on silica gel (*n*-hexane/ethyl acetate 75:25). The desired 3-allyl-2-hydroxybenzaldehyde (**5e**) was obtained as pale-yellow liquid in 53% yield.

^1H NMR (300 MHz, $CDCl_3$) δ 11.33 (d, *J* = 1.2 Hz, 1H), 9.92 (d, *J* = 1.3 Hz, 1H), 7.52 – 7.38 (m, 2H), 7.06 – 6.94 (m, 1H), 6.09 – 5.95 (m, 1H), 5.20 – 5.04 (m, 2H), 3.47 (dd, *J* = 6.6, 1.5 Hz, 2H). The data are in agreement with those reported in the literature [2].

2-Hydroxy-[1,1'-biphenyl]-3-carbaldehyde (5g)

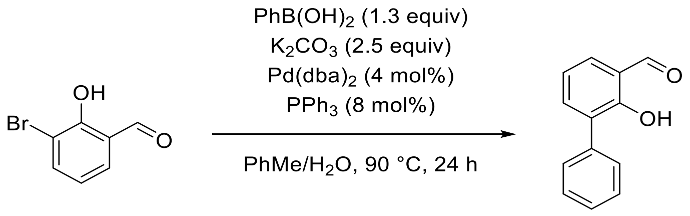

5d **5g**

3-Bromo-2-hydroxybenzaldehyde (**5d**, 500 mg, 2.48 mmol, 1 equiv), phenylboronic acid (392.6 mg, 3.22 mmol, 1.3 equiv), K_2CO_3 (856 mg, 6.20 mmol, 2.5 equiv), PPh_3 (52.4mg, 0.20 mmol, 0.08 equiv) and $Pd(dba)_2$ (57.04 mg, 0.04 mmol, 0.04 equiv) were suspended into a mixture 5:1 of toluene and water (20 mL), previously degassed with nitrogen for 30 minutes. The resulting mixture was stirred at 90 °C under nitrogen for 24 hours. The reaction mixture was then allowed to cool to room temperature, filtered on a small pad of celite and extracted with ethyl acetate (3 × 50 mL). The combined organic layers were then dried over anhydrous Na_2SO_4, filtered and concentrated under reduced pressure. The residue was purified by column chromatography on silica gel (*n*-hexane/ethyl acetate 95:5) followed by recrystallisation from EtOH. The desired product **5g** was obtained as a white solid (85% yield).

^1H NMR (300 MHz, $CDCl_3$) δ 11.54 (s, 1H), 9.96 (s, 1H), 7.67 – 7.53 (m, 4H), 7.51 – 7.34 (m, 3H), 7.11 (t, *J* = 7.6 Hz, 1H). The data are in agreement with those reported in the literature [3].

2-Hydroxy-2',4',6'-trimethyl-[1,1'-biphenyl]-3-carbaldehyde (5h)

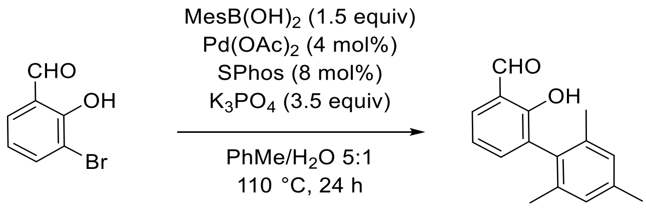

5d **5h**

3-Bromo-2-hydroxybenzaldehyde (**5d**, 1 g, 4.98 mmol, 1 equiv), mesitylboronic acid (1.2239 g, 7.47 mmol, 1.5 equiv), $Pd(OAc)_2$ (44.7 mg, 0.20 mmol, 0.04 equiv), SPhos (163.4 mg, 0.40 mmol, 0.08 equiv) and powdered, anhydrous K_3PO_4 (3.6962 g, 17.4 mmol, 3.5 equiv) were added, in this order, into a two-necked flask equipped with a reflux condenser. A mixture 5:1 of toluene and water (30 mL, to give a 0.17 M solution), previously degassed with nitrogen for 30 minutes, was added and the resulting mixture was refluxed under nitrogen for 24 hours. The resulting mixture was then allowed to cool to room temperature and extracted with ethyl acetate (3 × 20 mL). The combined organic layers were then dried over anhydrous sodium sulfate, filtered and concentrated under reduced pressure. The residue was purified by flash column chromatography on silica gel (eluent: from hexane to hexane/ethyl acetate 95:5). The desired product **5c** was obtained as a white solid (1.1355 g, 4.73 mmol, 95% yield).

^{1}H NMR (300 MHz, $CDCl_3$) δ 11.49 (s, 1H), 10.18 (s, 1H), 7.91 – 7.77 (m, 1H), 7.62 (d, *J* = 7.4 Hz, 1H), 7.37 (t, *J* = 7.5 Hz, 1H), 2.62 (s, 3H), 2.32 (s, 6H). The data are in agreement with those reported in the literature [4].

4-Methoxy-2,5-diphenylthiophene-3-carbaldehyde (5j)

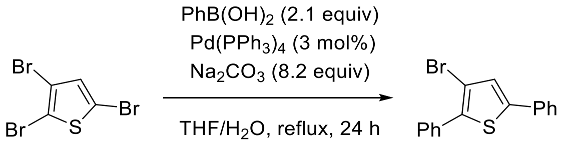

10 **11**

2,3,5-Tribromothiophene (**10**, 1 g, 3.11 mmol, 1 equiv) was added under nitrogen atmosphere to a biphasic mixture of anhydrous THF (0.23 M) and 20% w/v sodium carbonate aqueous solution (13.5 mL, 8.2 equiv), previously degassed with nitrogen for 20 minutes. Phenylboronic acid (758.4 mg, 6.22 mmol, 2.1 equiv) and $Pd(PPh_3)_4$ (250.3 mg, 0.093 mmol, 0.03 equiv) were subsequently added, and the mixture was refluxed for 24 hours. Then, THF was removed under reduced pressure, and the residue was extracted with CH_2Cl_2 (3 × 15 mL). The organic layers were collected, washed with brine (15 ml), dried over $MgSO_4$, filtered, and concentrated under vacuo. The residue was purified by column chromatography (*n*-hexane), obtaining 3-bromo-2,5-diphenylthiophene (**11**) as a light-yellow liquid (51% yield).

^{1}H NMR (300 MHz, $CDCl_3$) δ 7.75 – 7.66 (m, 2H), 7.65 – 7.52 (m, 2H), 7.50 – 7.27 (m, 7H). The data are in agreement with those reported in the literature [5].

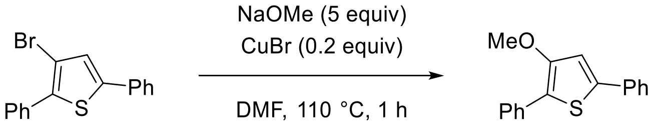

11 **12**

Sodium methoxide was prepared by dissolving sodium (181.6 mg, 7.93 mmol, 5 equiv) in dry methanol (6 mL) under nitrogen atmosphere at room temperature. When all the sodium was consumed, the sodium methoxide solution was added to 13.5 mL of dry DMF. Then, 2,5-diphenyl-3-bromothiophene (500 mg, 1.58 mmol, 1 equiv) and copper(I) bromide (45.3 mg, 0.316 mmol, 0.2 equiv) were added, and the reaction mixture was stirred at 110 °C for 30 minutes. The mixture was then filtered on a small pad of celite and washed with *n*-hexane (12 mL). The liquor was then washed with water (12 mL), and the aqueous phase was extracted twice with *n*-hexane (2 × 15 mL). The collected organic layers were dried over $MgSO_4$ and concentrated under reduced pressure. The residue was purified by column chromatography on silica gel (*n*-hexane/CH_2Cl_2 8:2) obtaining 3-methoxy-2,5-diphenylthiophene as a yellow solid (62% yield).

^{1}H NMR (300 MHz, $CDCl_3$) δ 7.77 (d, *J* = 8.2 Hz, 2H), 7.62 (d, *J* = 7.8 Hz, 2H), 7.50 – 7.10 (m, 7H), 3.97 (s, 3H). The data are in agreement with those reported in the literature [6].

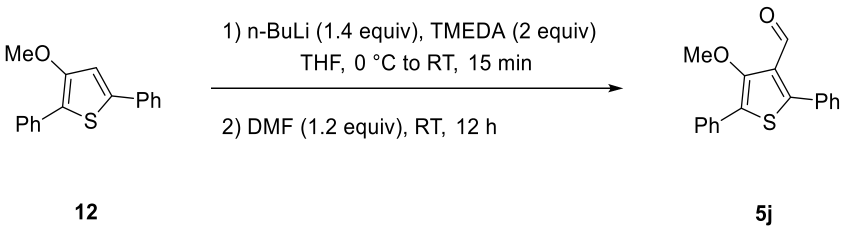

In a flame-dried Schlenk tube, under nitrogen atmosphere, at room temperature 3-methoxy-2,5-diphenylthiophene (250 mg, 0.94 mmol, 1 equiv) was dissolved in anhydrous THF (0.064 M) followed by the addition of distilled TMEDA (280.2 μL, 1.87 mmol, 2 equiv). The solution was cooled to 0 °C, and then *n*-BuLi solution (1.6 M in THF, 800 μL, 1.305 mmol, 1.4 equiv) was added dropwise. The mixture was left stirring for 15 minutes at 0 °C and then it was warmed up to room temperature. Dry DMF (86.9 μL, 1.12 mmol, 1.2 equiv) was then added dropwise, and the reaction mixture was left stirring for 12 hours at room temperature. A saturated solution of NH_4Cl (15 mL) was then added to quench the reaction and THF was removed under vacuum. After the aqueous solution was extracted with CH_2Cl_2 (3 × 15 mL), the collected organic phases were dried over $MgSO_4$, filtered and concentrated under reduced pressure. The residue was purified by flash column chromatography (*n*-hexane/CH_2Cl_2 6:4) to give 4-methoxy-2,5-diphenylthiophene-3-carbaldehyde as a yellow solid (57% yield).

^{1}H NMR (300 MHz, $CDCl_3$) δ 9.91 (s, 1H), 7.81 – 7.71 (m, 2H), 7.60 – 7.41 (m, 7H), 7.37 – 7.30 (m, 1H), 3.85 (s, 3H).

^{13}C NMR (75 MHz, $CDCl_3$) δ 185.51, 152.83, 152.59, 131.96, 131.61, 130.08, 129.87, 129.60, 129.03, 128.89, 128.82, 127.93, 127.68, 127.56, 61.88.

2.2. Diimines synthesis

General procedure A [7]

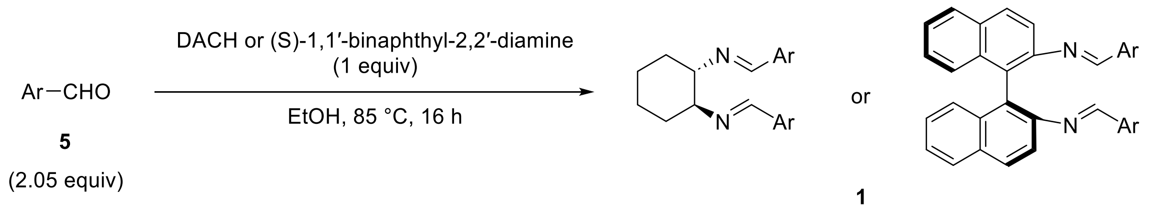

In a flame-dried Schlenk tube under nitrogen atmosphere, the enantiopure *trans*-1,2-diaminocyclohexane (DACH, 1 equiv) was dissolved in absolute EtOH (0.28 M). The corresponding aromatic aldehyde (2.05 equiv) was then added to the solution. The resulting yellow mixture was stirred at 85 °C for 16 hours. It was then cooled to room temperature and the solvent was removed under reduced pressure. The formation of the bisimine intermediate was verified through ^{1}H NMR analysis of the resulting crude. The crude imine was used directly in the next step without any further purification step.

General procedure B [8]

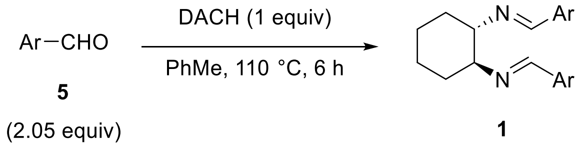

Enantiopure *trans*-1,2-diaminocyclohexane (DACH, 1 equiv) was added to a solution of the corresponding aromatic aldehyde (2.05 equiv) in toluene (0.8 M) and the obtained mixture was refluxed with a Dean–Stark apparatus for 6 hours. The mixture was cooled to room temperature and the solvent was removed by evaporation under reduced pressure. The formation of the bisimine intermediate was verified through ^{1}H NMR analysis of the resulting crude. The crude imine was used directly in the next step without any further purification step.

Diimines **1b**, **1c**, **1e**, **1f**, **1k** were synthetized according to **General procedure A**.

Diimines **1a**, **1d**, **1g**, **1h**, **1i**, **1j** were synthetized according to **General procedure B**.

3. Electrochemical imino-pinacol coupling in batch

3.1. Reaction conditions optimization

General procedure

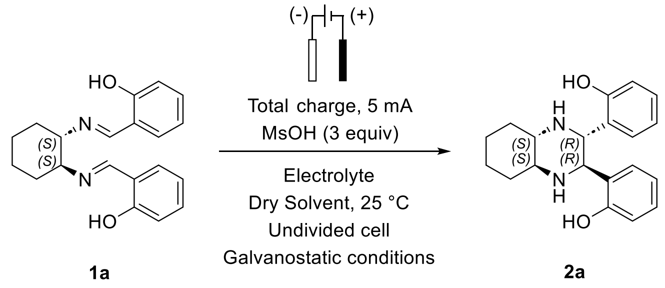

In a flame-dried, undivided cell equipped with two electrodes (distance between electrodes = 1 cm) and a stirring bar, diimine **1a** (0.5 mmol, 1 equiv) and the electrolyte were added, followed by two vacuum–nitrogen cycles. Under nitrogen atmosphere, dry solvent was added to the electrochemical cell. Methanesulfonic acid (0.1 mL, 1.5 mmol, 3 equiv) was then added. The reaction mixture was degassed by bubbling with argon for 20 minutes under vigorous stirring. The undivided cell was then connected to the Autolab power supply, as shown below (Figure S1), and stirred at 25 °C under galvanostatic conditions at 5 mA (2.5 mA/cm^2) until the desired total charge was delivered. The reaction mixture was poured into a beaker with 10 mL of distilled water, and a saturated solution of $NaHCO_3$ was added to adjust the pH to ≈7. Then, 10 mL of ethyl acetate were added, and the two phases were separated. The aqueous phase was then extracted with CH_2Cl_2 (3 × 10 mL). The combined organic layers were dried over Na_2SO_4, filtered and concentrated under vacuum. The reaction crude was purified by flash-column chromatography on silica gel (*n*-hexane/ethyl acetate 8:2) to give the desired pure product **2a**. In Table S1 the conditions tested are summarized.

Table S1. Screening of the imino-pinacol reaction conditions

Entry	Working (cathode)	Counter (anode)	Electrolyte	Electrolyte equivalents	Total charge (F/mol)	Solvent	Substrate concentration (M)	Yield **2a** (%)
1	Pt	Pt	NEt_4BF_4	2.6	2.2	MeCN	0.125	51
2	Pt	Pt	NEt_4BF_4	2.6	2.2	DMF	0.125	68
3[a]	Pt	Pt	NEt_4BF_4	2.6	2.2	DMF	0.125	-
4	Pt	Gr	NEt_4BF_4	2.6	2.2	DMF	0.125	64
5	Pt	Zn	NEt_4BF_4	2.6	2.2	DMF	0.125	64
6	Pt	Zn	NEt_4BF_4	2.6	2.5	DMF	0.125	61
7	Pt	Zn	NEt_4BF_4	2.6	2.5	DMF	0.250	64
8	SS	SS	NEt_4BF_4	2.6	2.2	DMF	0.125	47
9	GC	GC	NEt_4BF_4	2.6	2.2	DMF	0.125	85
10	GC	GC	NEt_4BF_4	1.3	2.2	DMF	0.125	50
11	GC	GC	NBu_4BF_4	2.6	2.2	DMF	0.125	72

[a] Reaction performed in the absence of MsOH

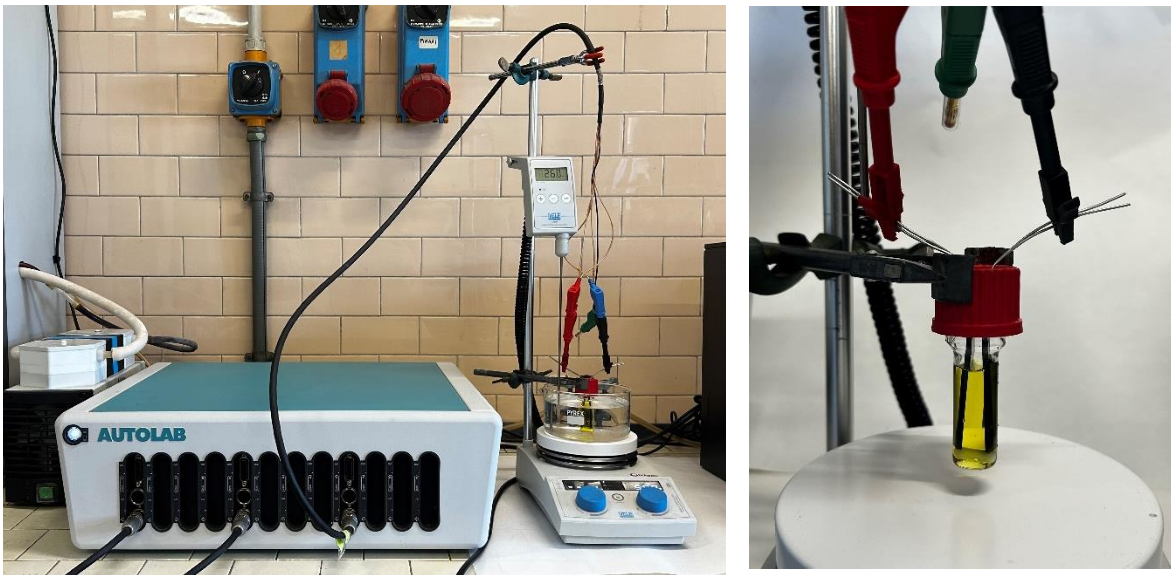

Figure S1. Setup in-batch electrochemical experiments

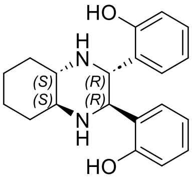

2a

Obtained as a white solid in 85% yield.

^{1}H-NMR (300 MHz, $CDCl_3$) δ 10.87 (s, 2H), 7.13 – 7.01 (m, 2H), 6.82 (d, *J* = 8.1 Hz, 2H), 6.42 (td, *J* = 7.4, 1.2 Hz, 2H), 6.10 (dd, *J* = 7.6, 1.6 Hz, 2H), 2.71 – 2.62 (m, 2H), 2.40 (s, 2H), 1.87 – 1.73 (m, 4H), 1.41 (d, *J* = 2.0 Hz, 2H).

^{13}C-NMR (75 MHz, $CDCl_3$) δ 157.08, 130.26, 129.10, 123.36, 118.73, 116.73, 63.62, 59.92, 31.72, 24.49.

HRMS (ES/QTOF): calcd m/z for $C_{20}H_{24}N_2O_2$ $[M+H]^+$, 325.1916; found, 325.1912

$[\alpha]_D^{20}$= + 4.89 (c = 1.0, $CHCl_3$) (Lit.[7] $[\alpha]_D^{20}$ = - 5.5 (c = 1.0, $CHCl_3$) for the opposite enantiomer)

The data are in agreement with those reported in the literature [7].

3.2. Reaction scope

General procedure

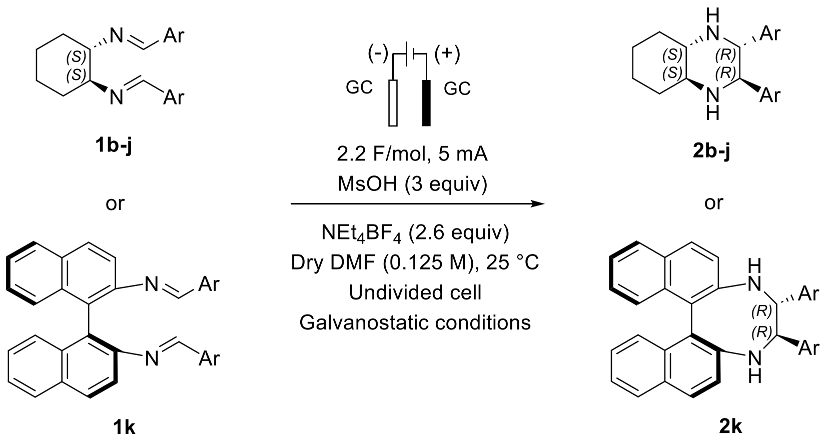

In a flame-dried, undivided cell equipped with two GC electrodes and a stirring bar, diimine **1b–k** (0.5 mmol, 1 equiv) and NEt_4BF_4 (1.3 mmol, 2.6 equiv) were added, followed by two vacuum–nitrogen cycles. Under nitrogen atmosphere, dry DMF (0.125 M) was added to the electrochemical cell. Methanesulfonic acid (0.1 mL, 1.5 mmol, 3 equiv) was then added. The reaction mixture was degassed by bubbling with argon for 20 minutes under vigorous stirring. The undivided cell was then connected to the Autolab power supply and stirred at 25 °C under galvanostatic conditions at 5 mA (2.5 mA/cm^2) until the desired total charge was delivered. The reaction mixture was poured into a beaker with 10 mL of distilled water, and a saturated solution of $NaHCO_3$ was added to adjust the pH to ≈7. Then, 10 mL of ethyl acetate were added, and the two phases were separated. The aqueous phase was then extracted with CH_2Cl_2 (3 × 10 mL). The combined organic layers were dried over Na_2SO_4, filtered and concentrated under vacuum. The reaction crude was purified by flash-column chromatography on silica gel (*n*-hexane/ethyl acetate 8:2) to give the desired pure product **2b–k**.

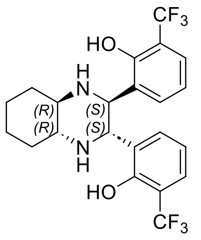

2b

Obtained as a yellow solid in 68% yield.

^{1}H-NMR (300 MHz, $CDCl_3$) δ 11.91 (s, 2H), 7.42 – 7.35 (m, 2H), 6.49 (dt, *J* = 10.4, 5.8 Hz, 2H), 6.27 – 6.17 (m, 2H), 4.26 (d, *J* = 3.9 Hz, 2H), 2.72 (d, *J* = 8.4 Hz, 2H), 2.56 (s, 2H), 1.82 (d, *J* = 11.4 Hz, 4H), 1.44 (d, *J* = 10.4 Hz, 4H).

^{13}C-NMR (75 MHz, $CDCl_3$) δ 155.53, 133.61, 126.60, 126.53, 125.69, 123.90, 117.91, 62.86, 59.52, 31.43, 24.24.

^{19}F-NMR (282 MHz, $CDCl_3$) δ -62.21.

HRMS (ES/QTOF): calcd m/z for $C_{22}H_{22}F_6N_2O_2$ [M-H]$^-$, 459.1507; found, 459.1511

$[\alpha]_D^{20}$ = - 39.90 (c = 1.0, $CHCl_3$)

Obtained as a yellow solid in 75% yield.

^{1}H-NMR (300 MHz, $CDCl_3$) δ 11.10 (s, 2H), 7.09 (d, *J* = 1.6 Hz, 2H), 6.69 (d, *J* = 7.9 Hz, 2H), 6.20 (d, *J* = 7.9 Hz, 2H), 4.22 (s, 2H), 2.72 (d, *J* = 7.7 Hz, 2H), 2.46 (bs, 2H), 1.88-1.71 (m, 4H), 1.50-1.39 (m, 4H).

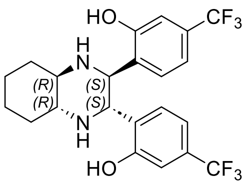

2c

^{13}C-NMR (75 MHz, $CDCl_3$) δ 158.58, 158.17, 157.24, 131.57 (q, *J* = 32.3 Hz), 130.38, 129.33, 126.30, 125.61, 122.01, 121.39, 117.43, 117.35, 115.30, 115.25, 114.08, 114.04, 113.98, 62.83, 61.52, 59.67, 59.61, 57.96, 31.45, 31.28, 24.23 (presence of different rotamers).

^{19}F-NMR (282 MHz, $CDCl_3$) δ -57.51, -58.02, -62.79, -62.86 (presence of different rotamers).

HRMS (ES/QTOF): calcd m/z for $C_{22}H_{22}F_6N_2O_2$ $[M-H]^-$, 459.1507; found, 459.1508

$[\alpha]_D^{20}$ = - 19.70 (c = 1.0, $CHCl_3$)

Obtained as a white solid in 70% yield.

^{1}H-NMR (300 MHz, $CDCl_3$) δ 11.87 (s, 2H), 7.35 (dd, *J* = 8.0, 1.6 Hz, 2H), 6.34 (t, *J* = 7.8 Hz, 2H), 6.07 (dd, *J* = 7.6, 1.6 Hz, 2H), 4.16 (s, 2H), 2.71 (s, 2H), 2.49 (s, 2H), 1.93 – 1.69 (m, 4H), 1.50 – 1.32 (m, 2H).

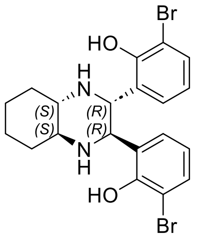

2d

^{13}C-NMR (75 MHz, $CDCl_3$) δ 153.80, 132.79, 129.52, 124.26, 119.74, 111.00, 63.11, 59.66, 31.63, 24.41.

HRMS (ES/QTOF): calcd m/z for $C_{20}H_{22}Br_2N_2O_2$ $[M+H]^+$, 483.0106; found, 483.0110

$[\alpha]_D^{20}$ = + 107.72 (c = 1.0, $CHCl_3$)

Obtained as a pale-yellow oil in 77% yield.

^{1}H-NMR (300 MHz, $CDCl_3$) δ 11.07 (bs, 2H), 6.95 (dd, *J* = 7.6, 1.7 Hz, 2H), 6.38 (t, *J* = 7.5 Hz, 2H), 6.17 – 5.95 (m, 4H), 5.16 – 4.98 (m, 4H), 4.18 (s, 2H), 3.41 (dt, *J* = 6.3, 1.6 Hz, 4H), 2.74 – 2.62 (m, 2H), 2.42 (bs, 2H), 1.85 – 1.73 (m, 4H), 1.49 – 1.37 (m, 4H).

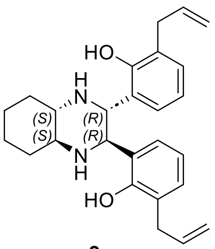

2e

^{13}C-NMR (75 MHz, $CDCl_3$) δ 154.67, 137.28, 129.41, 128.27, 127.09, 122.91, 118.30, 115.02, 63.37, 59.74, 33.92, 31.63, 24.39.

HRMS (ES/QTOF): calcd m/z for $C_{26}H_{32}N_2O_2$ $[M+H]^+$, 405.2542; found, 405.2539

$[\alpha]_D^{20}$ = + 34.66 (c = 1.0, $CHCl_3$)

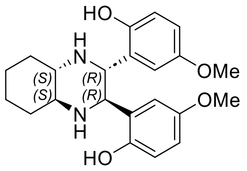

2f

Obtained as a pale-yellow solid in 71% yield.

^{1}H-NMR (300 MHz, $CDCl_3$) δ 6.39 (d, *J* = 2.2 Hz, 2H), 6.10 – 5.97 (m, 4H), 4.05 (s, 2H), 3.72 (s, 6H), 2.64 (d, *J* = 8.1 Hz, 2H), 1.78 (t, *J* = 9.8 Hz, 4H), 1.41 (d, *J* = 6.7 Hz, 4H).

^{13}C-NMR (75 MHz, $CDCl_3$) δ 160.42, 158.07, 130.92, 115.82, 104.69, 101.96, 63.22, 59.83, 55.16, 31.60, 24.35.

HRMS (ES/QTOF): calcd m/z for $C_{22}H_{28}N_2O_4$ $[M+H]^+$, 385.2127; found, 385.2124

$[\alpha]_D^{20}$ = + 19.70 (c = 1.0, $CHCl_3$)

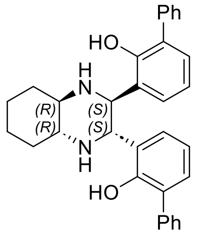

2g

Obtained as a white solid in 68% yield.

^{1}H-NMR (600 MHz, $CDCl_3$) δ 11.44 (s, 2H), 7.67 – 7.62 (m, 4H), 7.49 – 7.42 (m, 4H), 7.38 – 7.31 (m, 2H), 7.19 (dd, *J* = 7.6, 1.7 Hz, 2H), 6.54 (t, *J* = 7.5 Hz, 2H), 6.20 (dd, *J* = 7.5, 1.7 Hz, 2H), 4.32 (s, 2H), 2.70 – 2.63 (m, 2H), 2.46 (s, 2H), 1.81 – 1.71 (m, 4H), 1.37 (t, *J* = 8.3 Hz, 4H).

^{13}C-NMR (151 MHz, $CDCl_3$) δ 154.13, 138.75, 130.25, 129.69, 129.59, 128.19, 126.94, 123.74, 118.85, 63.55, 59.75, 31.65, 24.48.

HRMS (ES/QTOF): calcd m/z for $C_{32}H_{32}N_2O_2$ $[M+H]^+$, 477.2542; found, 477.2549

$[\alpha]_D^{20}$ = - 265.12 (c = 1.0, $CHCl_3$)

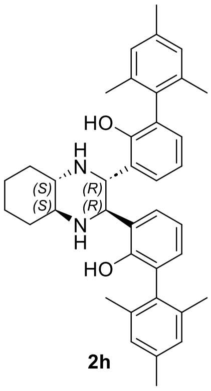

2h

Obtained as a white solid in 87% yield.

^{1}H-NMR (300 MHz, $CDCl_3$) δ 10.95 (s, 2H), 6.94 (dd, *J* = 20.2, 7.8 Hz, 6H), 6.58 – 6.48 (m, 2H), 6.20 (d, *J* = 7.5 Hz, 2H), 4.25 (s, 2H), 2.62 (d, *J* = 6.6 Hz, 2H), 2.33 (s, 6H), 2.17 (s, 6H), 2.04 (s, 6H), 1.74 (s, 4H), 1.35 – 1.29 (m, 4H), 1.23 – 1.18 (m, 2H).

^{13}C-NMR (75 MHz, MeOD) δ 153.14, 138.90, 138.39, 138.23, 133.96, 133.60, 129.65, 129.43, 129.34, 121.49, 119.45, 58.98, 28.87, 24.51, 21.18, 20.65, 20.58.

HRMS (ES/QTOF): calcd m/z for $C_{38}H_{44}N_2O_2$ $[M+H]^+$, 561.3481; found, 561.3489

$[\alpha]_D^{20}$ = + 135.16 (c = 1.0, $CHCl_3$)

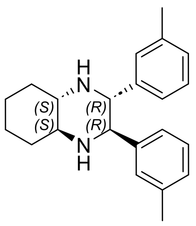

2i

Obtained as a yellow solid in 67% yield.

^{1}H-NMR (300 MHz, $CDCl_3$) δ 6.87 (dd, *J* = 8.4, 2.2 Hz, 2H), 6.72 (d, *J* = 8.2 Hz, 2H), 5.84 (d, *J* = 2.2 Hz, 2H), 4.03 (s, 2H), 2.66 (d, *J* = 7.4 Hz, 2H), 1.91 (s, 6H), 1.84 – 1.72 (m, 4H), 1.48 – 1.37 (m, 4H).

^{13}C-NMR (75 MHz, $CDCl_3$) δ 154.59, 131.37, 130.96, 130.73, 130.32, 129.25, 127.40, 122.72, 116.11, 63.73, 59.73.

$[\alpha]_D^{20}$ = + 78.30 (c = 1.0, $CHCl_3$)

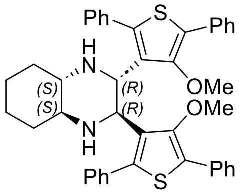

2j

Obtained as a yellow solid in 35% yield.

^{1}H-NMR (300 MHz, $CDCl_3$) δ 7.41 – 7.28 (m, 10H), 7.24 – 7.12 (m, 10H), 4.30 (s, 2H), 3.02 (s, 6H), 2.29 (d, *J* = 9.3 Hz, 4H), 1.92 (d, *J* = 12.6 Hz, 2H), 1.81 (d, *J* = 9.0 Hz, 2H), 1.38 (d, *J* = 10.2 Hz, 2H).

^{13}C-NMR (151 MHz, $CDCl_3$) δ 152.32, 137.54, 134.77, 133.07, 131.23, 129.43, 128.30, 128.24, 128.21, 127.62, 127.00, 126.80, 123.67, 61.18, 59.95, 58.33, 32.62, 29.70, 25.69.

HRMS (ES/QTOF): calcd m/z for $C_{42}H_{40}N_2O_2S_2$ $[M+H]^+$, 669.2609; found, 669.2608

$[\alpha]_D^{20}$ = + 240.01 (c = 1.0, $CHCl_3$)

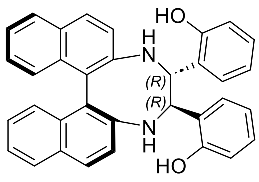

2k

Obtained as a yellow solid in 34% yield.

^{1}H-NMR (300 MHz, $CDCl_3$) δ 8.03 – 7.88 (m, 4H), 7.60 (d, *J* = 8.5 Hz, 2H), 7.49 (d, *J* = 8.3 Hz, 4H), 7.38-7.28 (s, 3H), 7.25-7.11 (m, 3H), 7.03 (d, *J* = 8.1 Hz, 2H), 6.94 – 6.75 (m, 2H), 6.71 (d, *J* = 8.1 Hz, 1H), 6.58 (t, *J* = 7.3 Hz, 1H), 5.98 (d, *J* = 7.8 Hz, 1H), 5.00 (d, *J* = 9.4 Hz, 1H), 4.67 (d, *J* = 9.3 Hz, 1H), 4.13 (d, *J* = 9.0 Hz, 1H), 3.96 (d, *J* = 9.2 Hz, 1H), 1.59 (bs, 12H) (isolated as mixture of conformers)

Rapidly degraded, unable to perform the characterization of the compound.

4. In-flow electrochemical imino-pinacol coupling

4.1. Reaction conditions optimization

General procedure

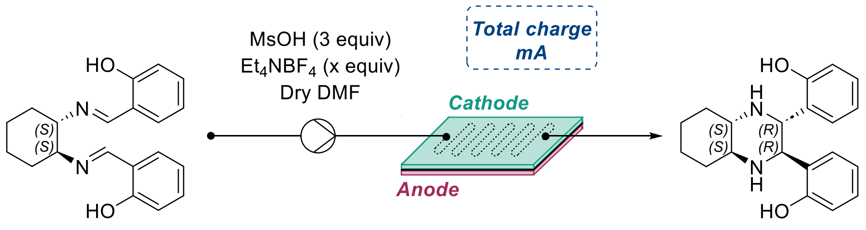

Diimine **1a** (0.1 mmol, 1 equiv) and NEt_4BF_4 were added in a flame-dried Erlenmeyer flask with a stirring bar, followed by two vacuum–nitrogen cycles. Under nitrogen atmosphere, dry DMF (0.125 M) and methanesulfonic acid (0.3 mmol, 3 equiv) were then added to the reaction mixture, which was degassed by bubbling with argon for 20 minutes under vigorous stirring.

In-flow experiments were performed using the Asia (Syrris) modular system, which consists of a syringe pump module and the FLUX electrochemistry module, as shown below (Figure S2). The reaction mixture was pumped through the syringe pump module to the electrochemical cell via a PFA tube immersed in the reaction mixture. After passing through the electrochemical cell, the reaction mixture was collected through the outlet PFA tube. A thorough investigation of the flow reaction conditions was performed by varying the electrodes used, the current intensity, the total charge, the flow rate, and the equivalents of the electrolyte used. Each experiment was performed until the desired total charge was delivered.

The best results in terms of productivity and space-time yield (STY) were obtained using two carbon filled PPS electrodes, a constant current of 80 mA, a total charge of 4.15 F/mol, a flow rate of 96 μL/min, a residence time of 2.34 minutes and 1.3 equivalents of NEt_4BF_4.

The reaction mixture was poured into a beaker with 5 mL of distilled water, and a saturated solution of $NaHCO_3$ was added to adjust the pH to ≈7. Then, 5 mL of ethyl acetate were added, and the two phases were separated. The aqueous phase was then extracted with CH_2Cl_2 (3 × 5 mL). The combined organic layers were dried over Na_2SO_4, filtered and concentrated under vacuum.

Using the optimal conditions, desired product **2a** was obtained in 56% yield (^{1}H NMR yield, calculated on the reaction crude using 1,3,5-trimethoxybenzene as internal standard, d1 = 10 s).

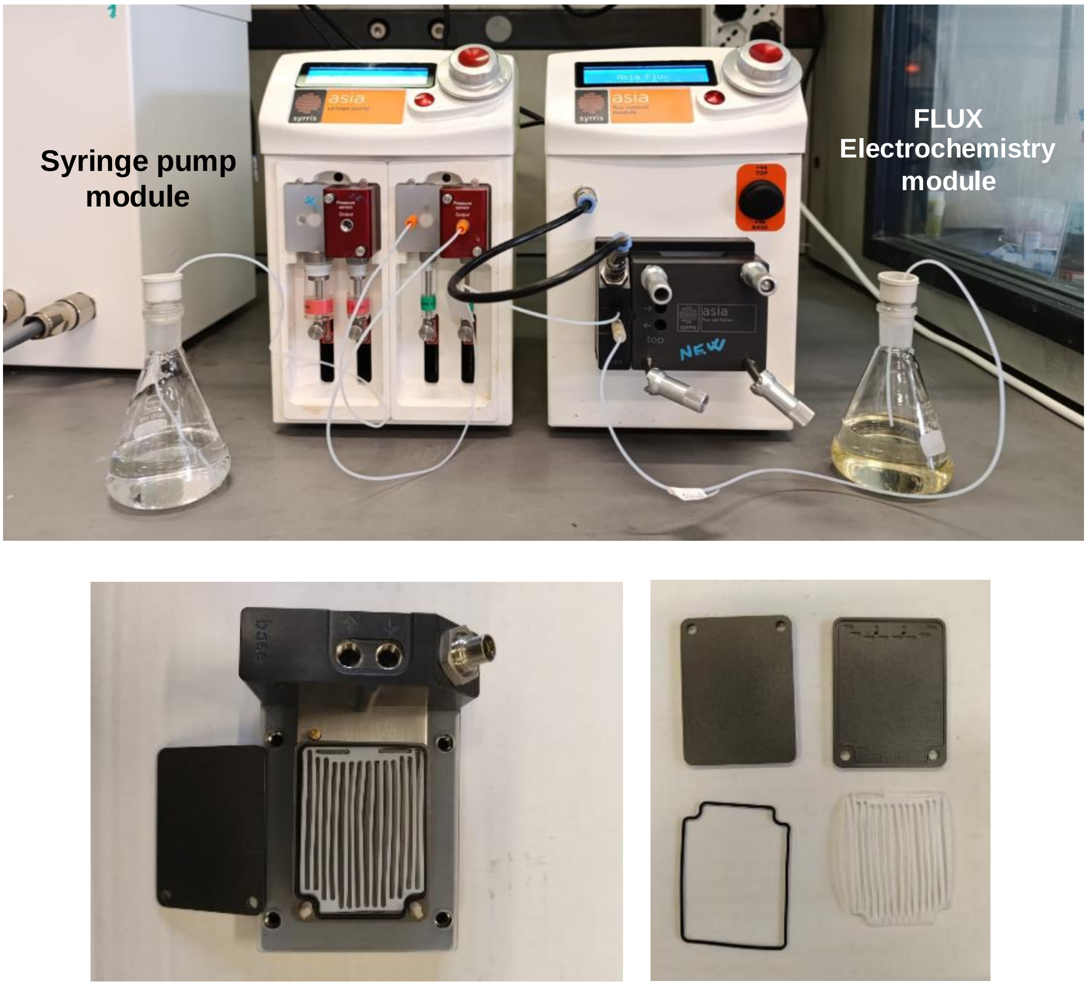

Open electrochemical cell

Figure S2. Setup in-flow electrochemical experiments

4.2. Large-scale reaction

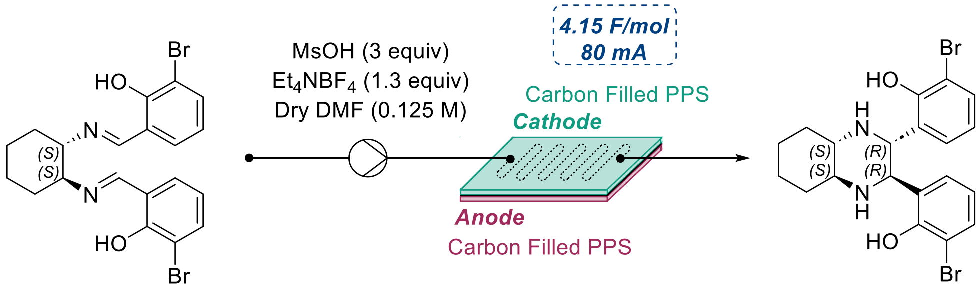

Diimine **1d** (2.5 mmol, 1 equiv) and NEt_4BF_4 (3.25 mmol, 1.3 equiv) were added in a flame-dried Erlenmeyer flask with a stirring bar, followed by two vacuum–nitrogen cycles. Under nitrogen atmosphere, dry DMF (0.125 M) and methanesulfonic acid (7.5 mmol, 3 equiv) were then added to the reaction mixture, which was degassed by bubbling with argon for 20 minutes under vigorous stirring. The reaction was performed using the Asia (Syrris) modular system with two carbon filled PPS electrodes, a constant current of 80 mA, a flow rate of 96 μL/min and a residence time of 2.34 minutes. The experiment was conducted until a total charge of 4.15 F/mol was delivered.

The reaction mixture was then poured into a beaker with 50 mL of distilled water, and a saturated solution of $NaHCO_3$ was added to adjust the pH to ≈7. Then, 30 mL of ethyl acetate were added, and the two phases were separated. The aqueous phase was then extracted with CH_2Cl_2 (3 × 20 mL). The combined organic layers were dried over Na_2SO_4, filtered and concentrated under reduced pressure to give the crude product that was purified through flash column chromatography on silica gel (eluent: from hexane/ethyl acetate 9:1 to hexane/ethyl acetate 1:1). The desired product **2d** was obtained in 52% yield as a white solid.

4.3 Productivity and space-time yield calculation

The performances of the experiments under continuous conditions were evaluated in terms of productivity and space-time yield and compared to the same values calculated for the batch reactions.

Productivity is defined as the mmol of product per hour provided by the system:

$$\text{Productivity} = \frac{\text{mmol limiting reagent} \cdot \text{yield}}{\text{time (h)}}$$

The space-time yield (STY) instead, is defined as the mmol of product per hour, per unit of volume:

$$\text{STY} = \frac{\text{mmol limiting reagent} \cdot \text{yield}}{\text{time (h)} \cdot \text{volume (mL)}}$$

Results are reported in Table S2.

Table S2. Comparison of productivity and space-time yield of batch and flow processes for the synthesis of piperazines **2a** and **2d**

Entry	Product	Method	Productivity [a] (mmol/h)	Productivity Rel. factor	Space-time yield [b] (mmol/mL*h)	Space-time yield Rel. factor
1	**2a**	Batch	0.065	1	0.016	1
2	**2a**	Flow	0.403	6.2	1.795	112.2
3	**2d**	Batch	0.036	1	0.009	1
4	**2d**	Flow	0.374	10.4	1.662	184.7

[a] *Productivity:* moles of product divided by the collection time required to collect the product obtained by the reaction of 0.5 mmol of diimines **1a** and **1d**. [b] *Space-time yield:* moles of product in the reactor, divided by residence time and reactor volume

5. Cyclic voltammetry analyses

Cyclic voltammetry analyses were carried out using Metrohm Autolab M204 potentiostat. The measurements were performed under an argon atmosphere using two glassy carbon electrodes as working and counter and Ag/AgCl as reference electrodes. All CV analyses were recorded in a 0.325 M solution of Et_4NBF_4 in dry DMF with 0.1 V/s scan rate. The solvent was degassed by bubbling through Ar for 30 minutes before scan was initiated. The CV were carried out at room temperature (25 °C).

Cyclic voltammetry of a 0.325 M solution of Et_4NBF_4 in DMF.

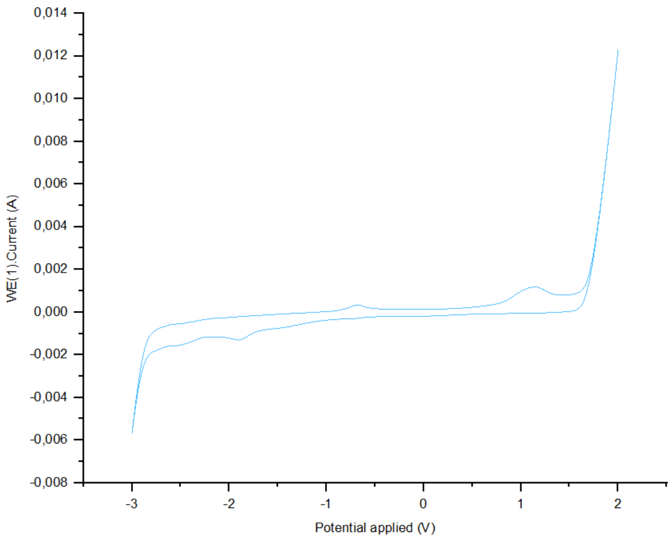

Cyclic voltammetry of diimine **1a** (10 mM) recorded in a 0.325 M solution of Et_4NBF_4 in DMF.

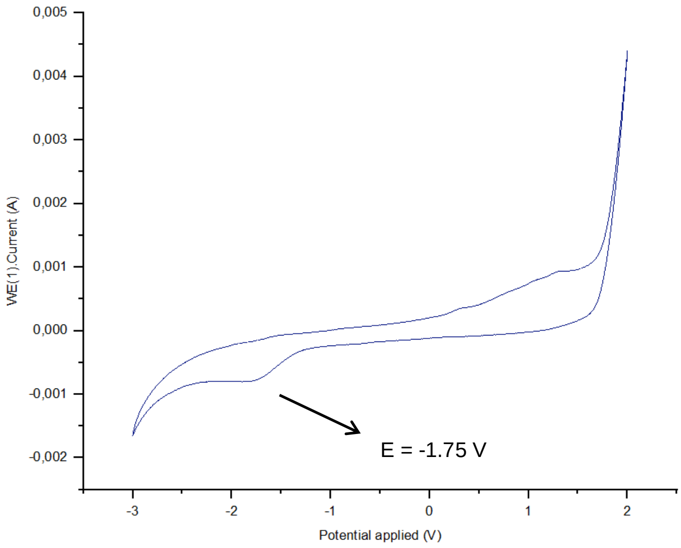

Cyclic voltammetry of diimine **1a** (10 mM) in presence of 1 equivalent of methanesulfonic acid (10 mM) recorded in a 0.325 M solution of Et_4NBF_4 in DMF.

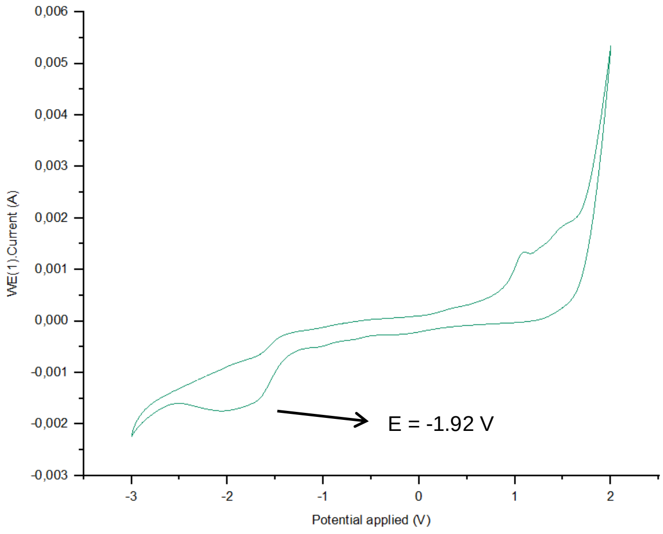

Cyclic voltammetry of diimine **1a** (10 mM) in presence of 2 equivalent of methanesulfonic acid (20 mM) recorded in a 0.325 M solution of Et_4NBF_4 in DMF.

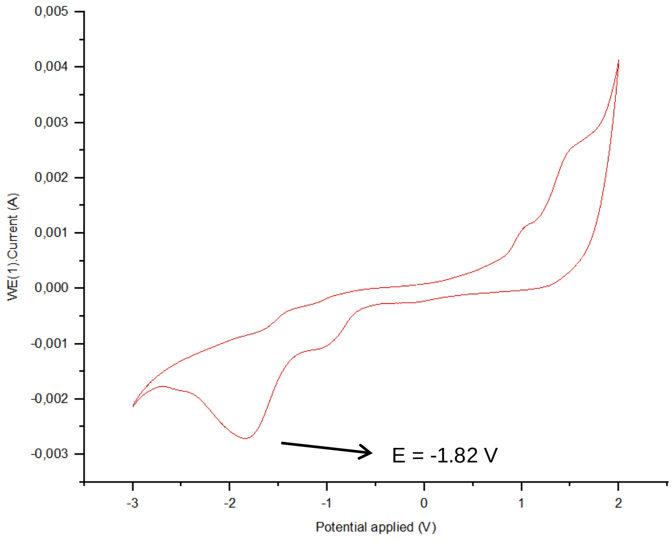

Cyclic voltammetry of diimine **1a** (10 mM) in presence of 3 equivalent of methanesulfonic acid (30 mM) recorded in a 0.325 M solution of Et_4NBF_4 in DMF.

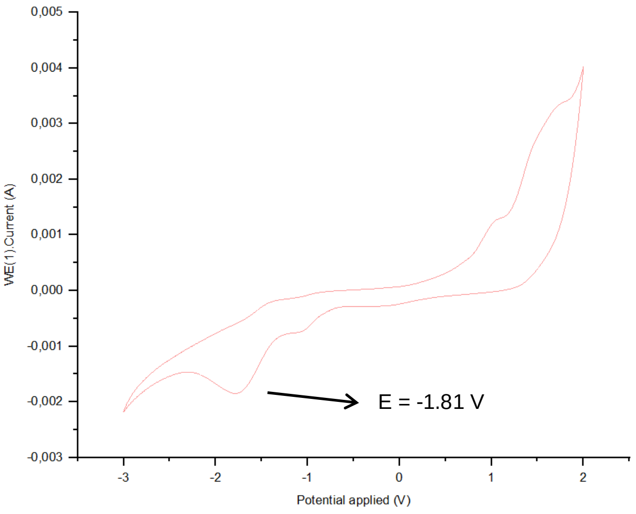

Cyclic voltammetry of a 0.325 M solution of Et_4NBF_4 in DMF (light-blue line). Cyclic voltammetry of diimine **1a** (10 mM) recorded in a 0.325 M solution of Et_4NBF_4 in DMF (dark-blue line). Cyclic voltammetry of diimine **1a** (10 mM) in presence of 1 equivalent of methanesulfonic acid (10 mM) recorded in a 0.325 M solution of Et_4NBF_4 in DMF (green line). Cyclic voltammetry of diimine **1a** (10 mM) in presence of 2 equivalent of methanesulfonic acid (20 mM) recorded in a 0.325 M solution of Et_4NBF_4 in DMF (dark-red line). Cyclic voltammetry of diimine **1a** (10 mM) in presence of 3 equivalent of methanesulfonic acid (30 mM) recorded in a 0.325 M solution of Et_4NBF_4 in DMF (light-red line).

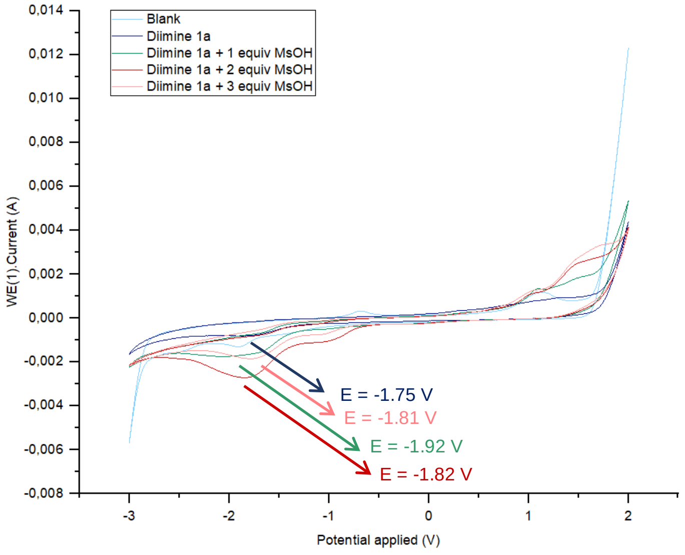

6. NMR spectra and HRMS

1H NMR compound 7

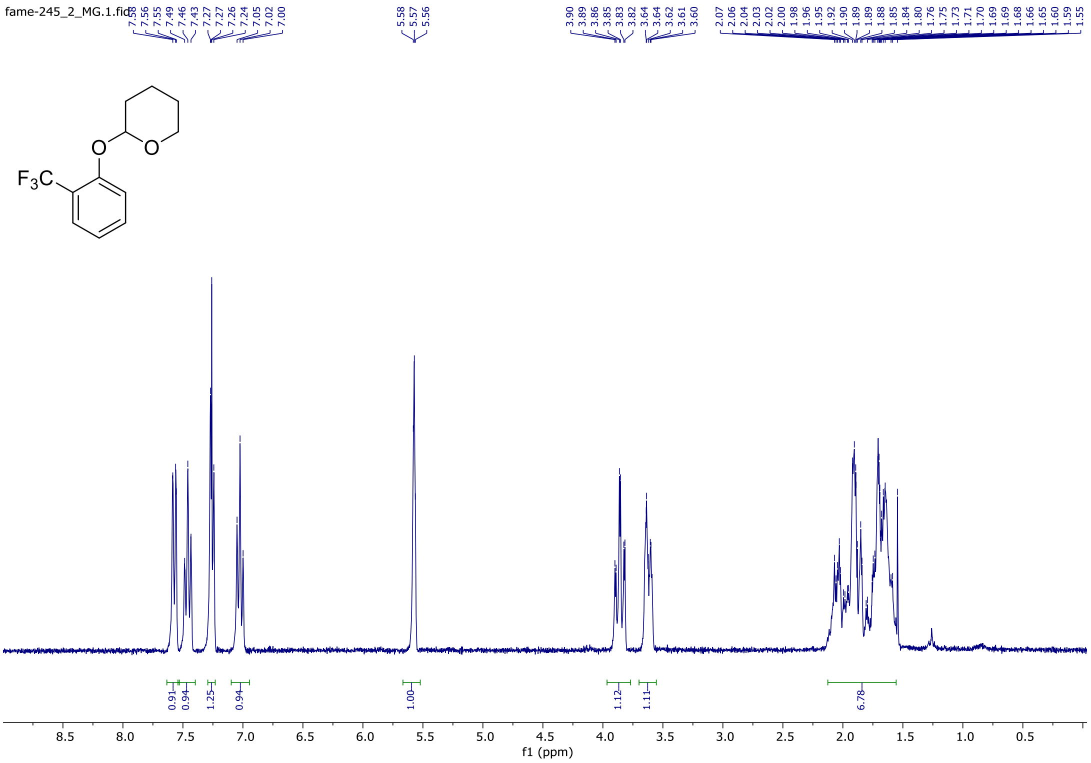

1H NMR compound 5b

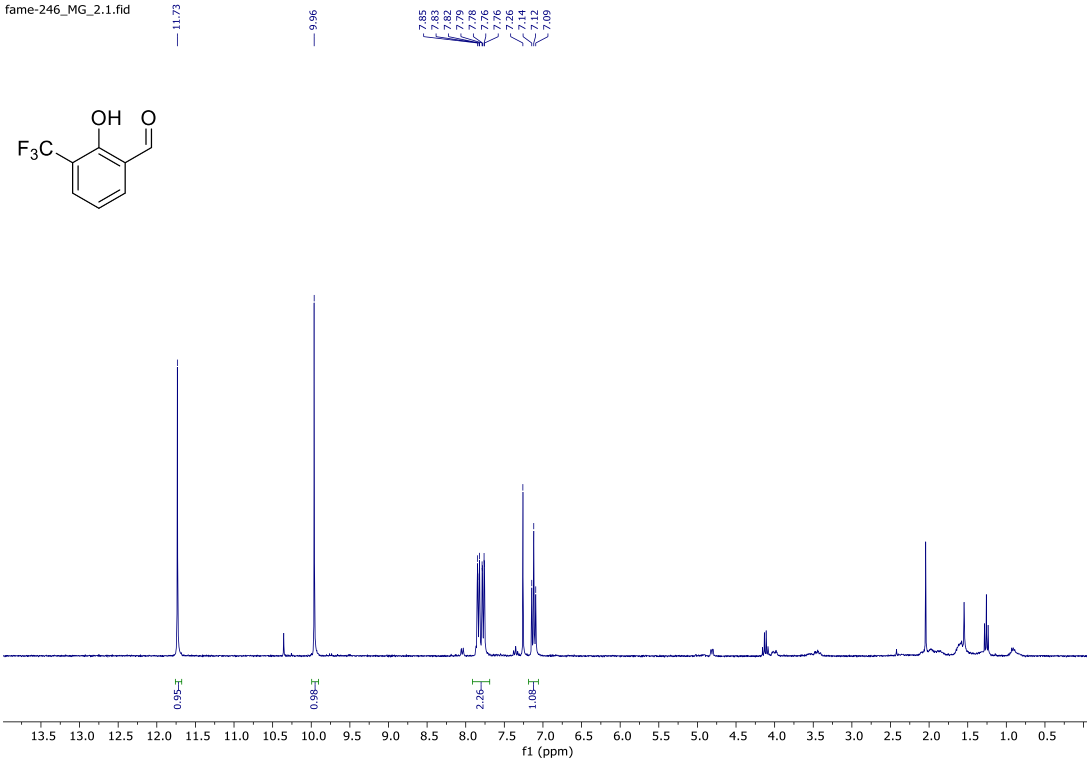

^{1}H NMR compound 5c

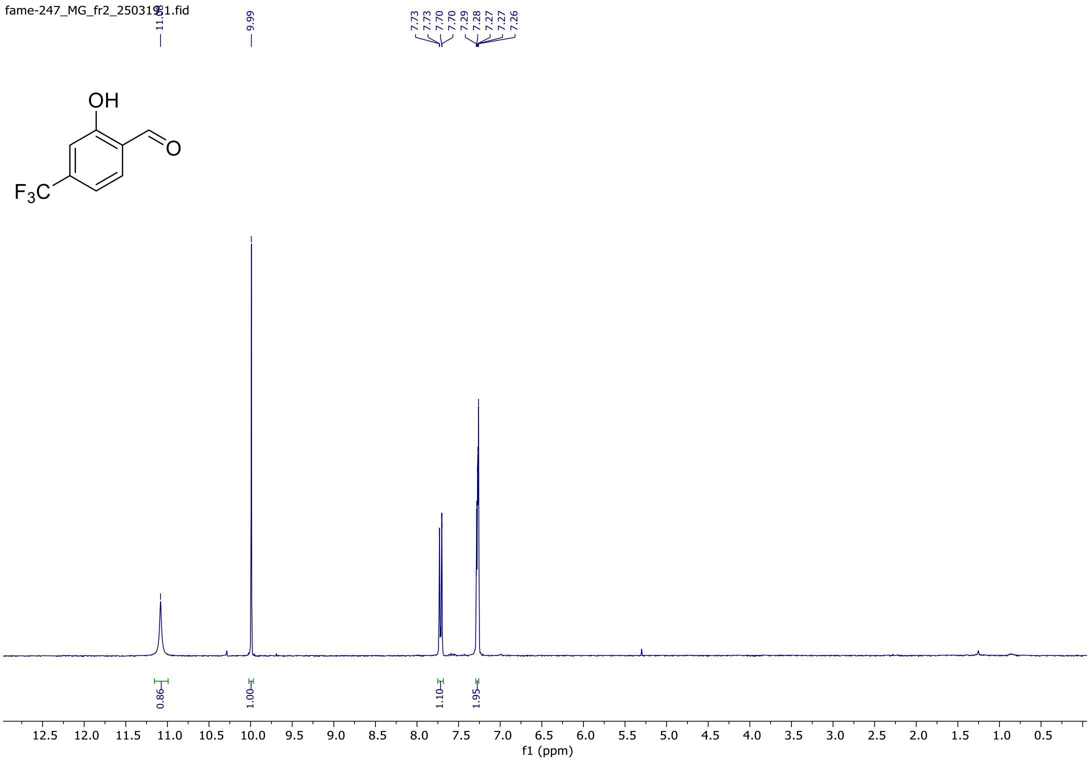

^{1}H NMR compound 5e

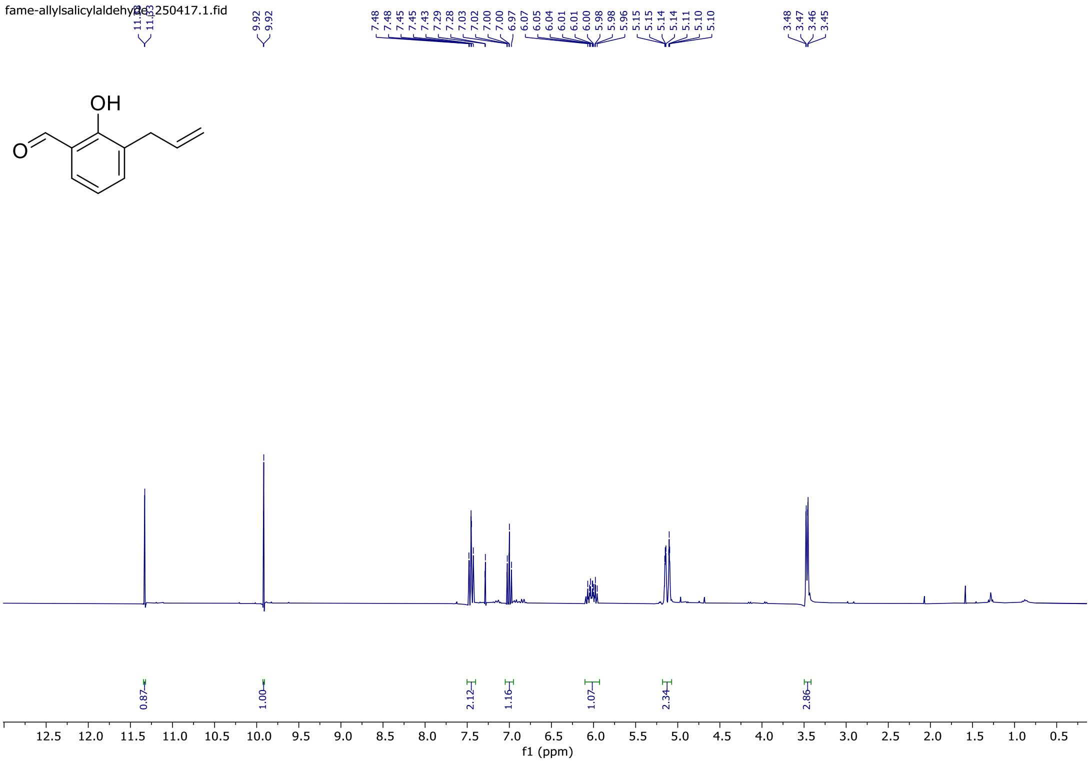

^{1}H NMR compound 5g

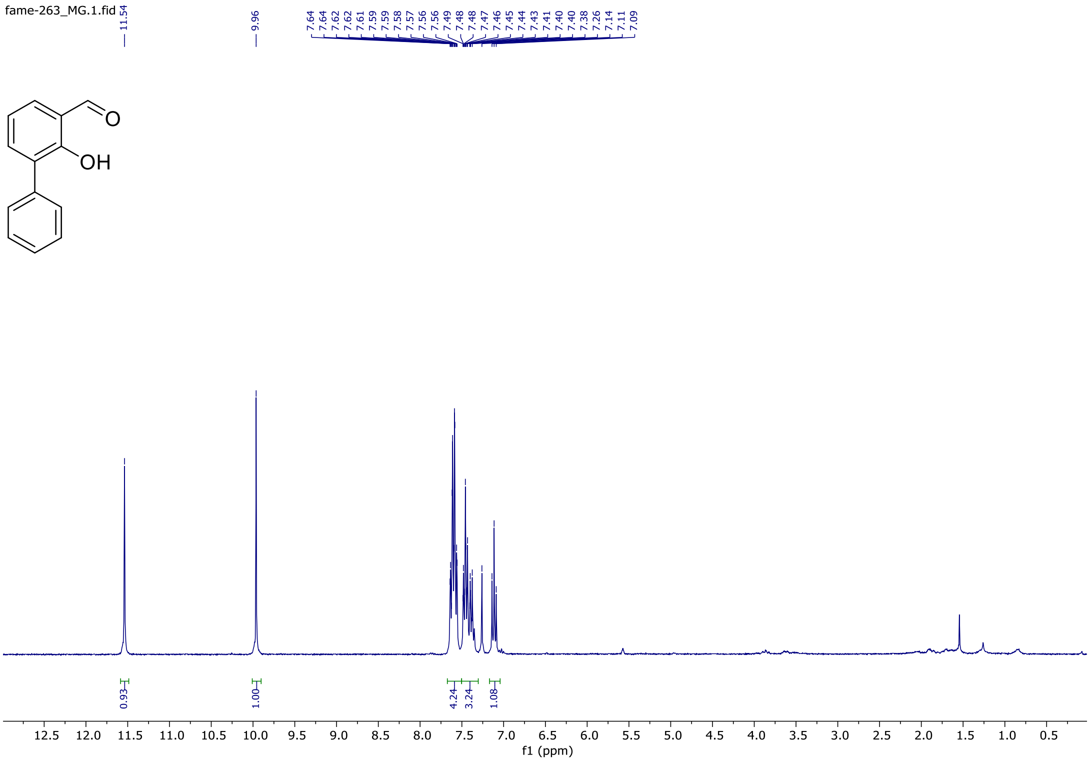

^{1}H NMR compound 5h

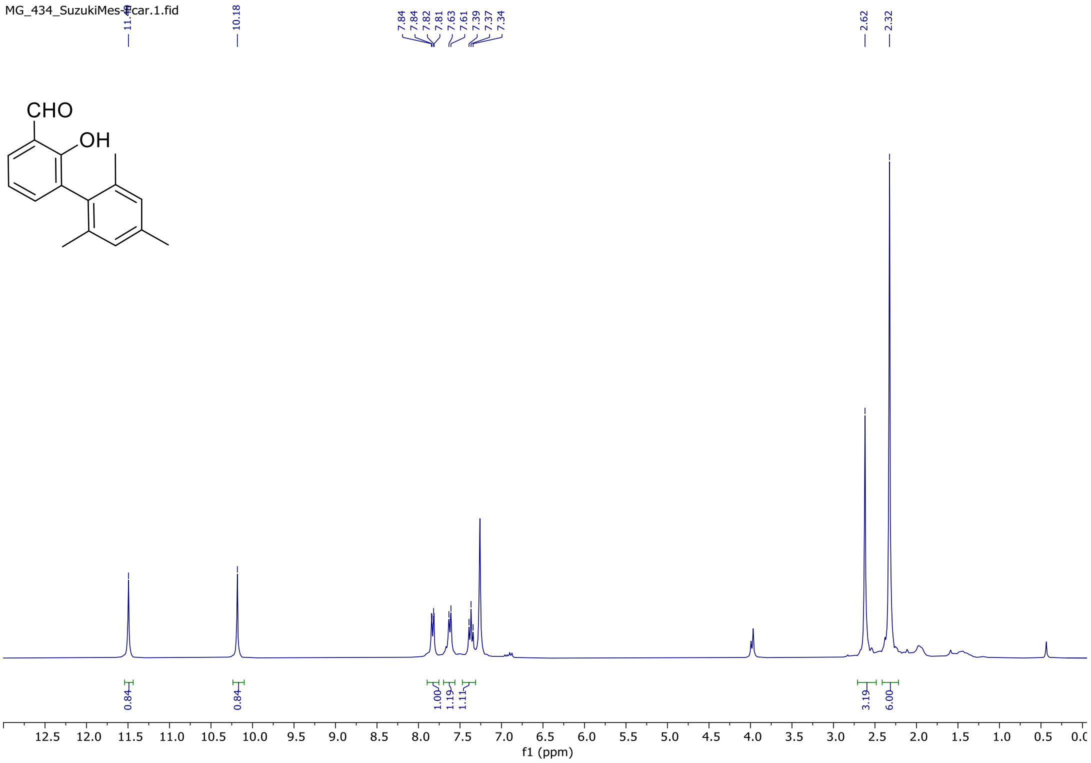

^{1}H NMR compound 11

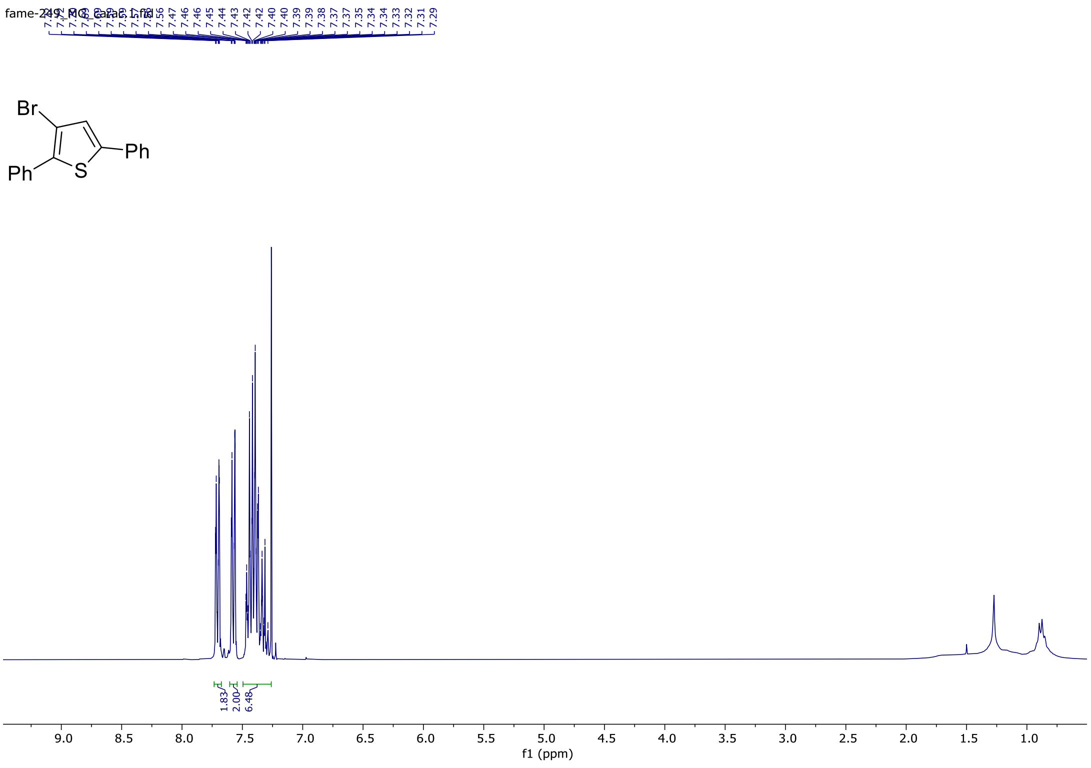

^{1}H NMR compound 12

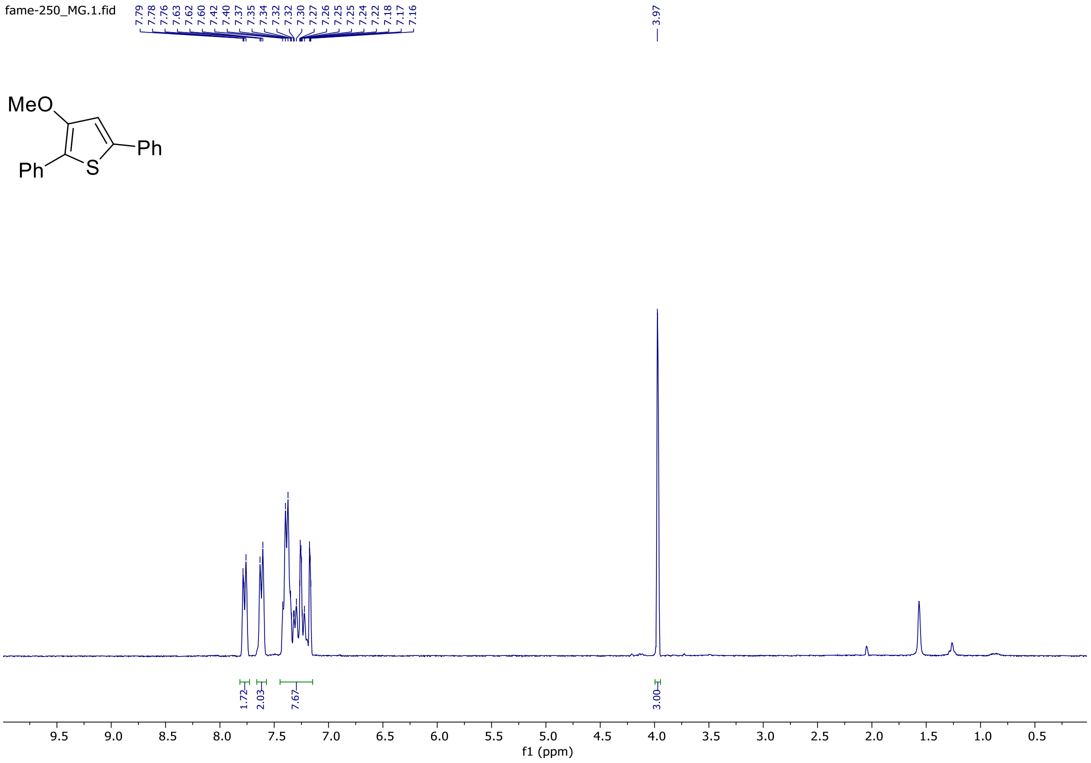

^{1}H NMR compound 5j

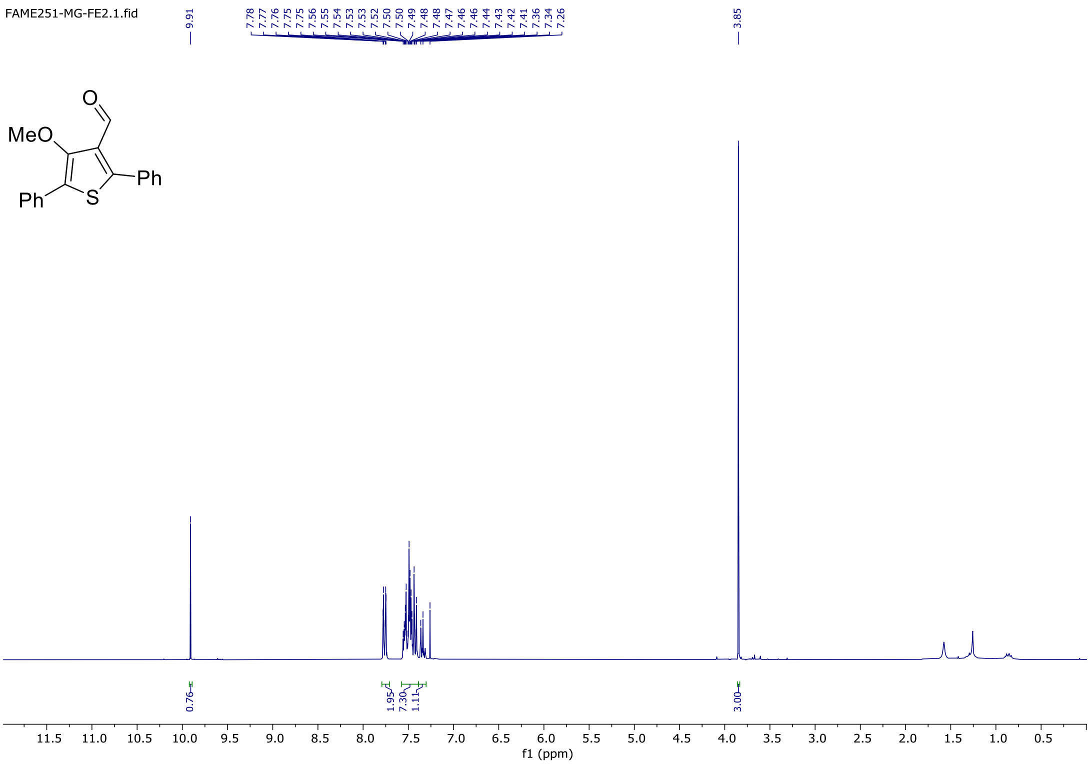

^{13}C NMR compound 5j

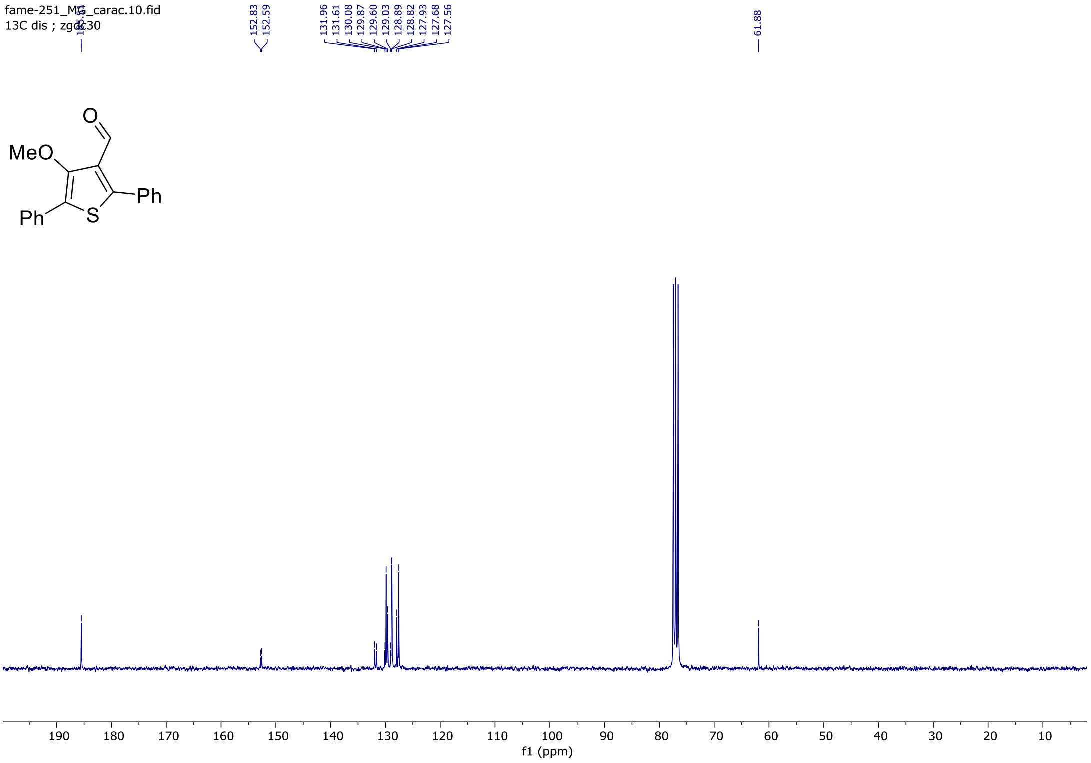

^{1}H NMR compound 2a

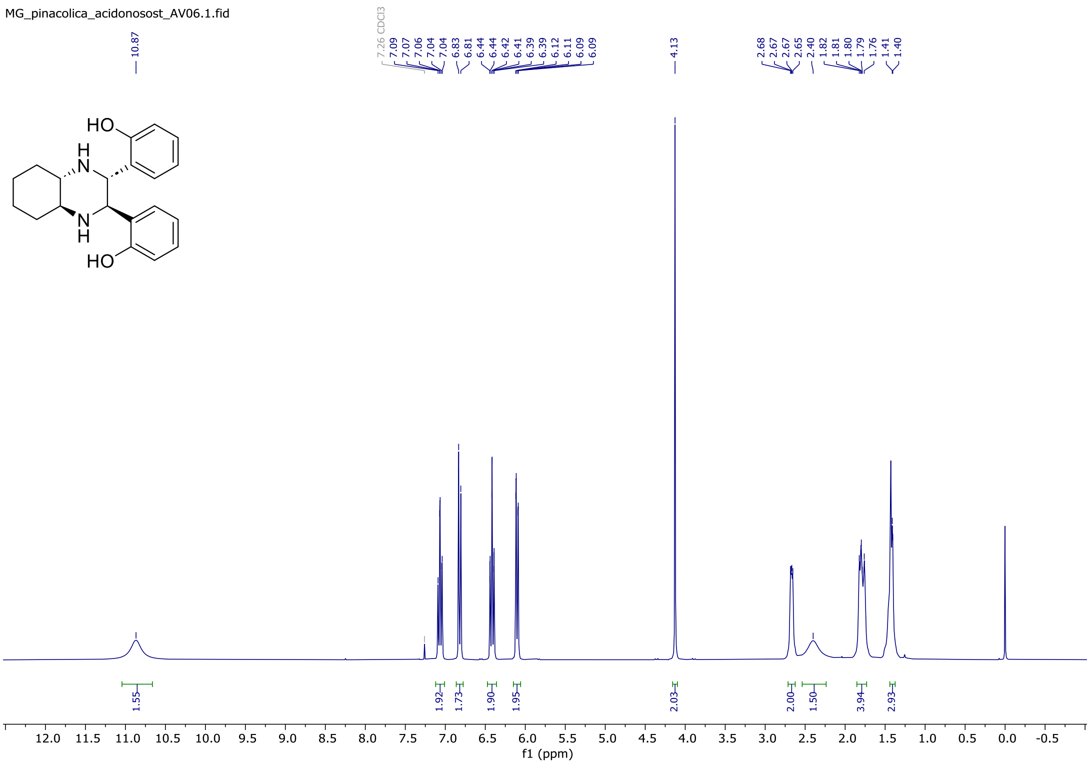

^{13}C NMR compound 2a

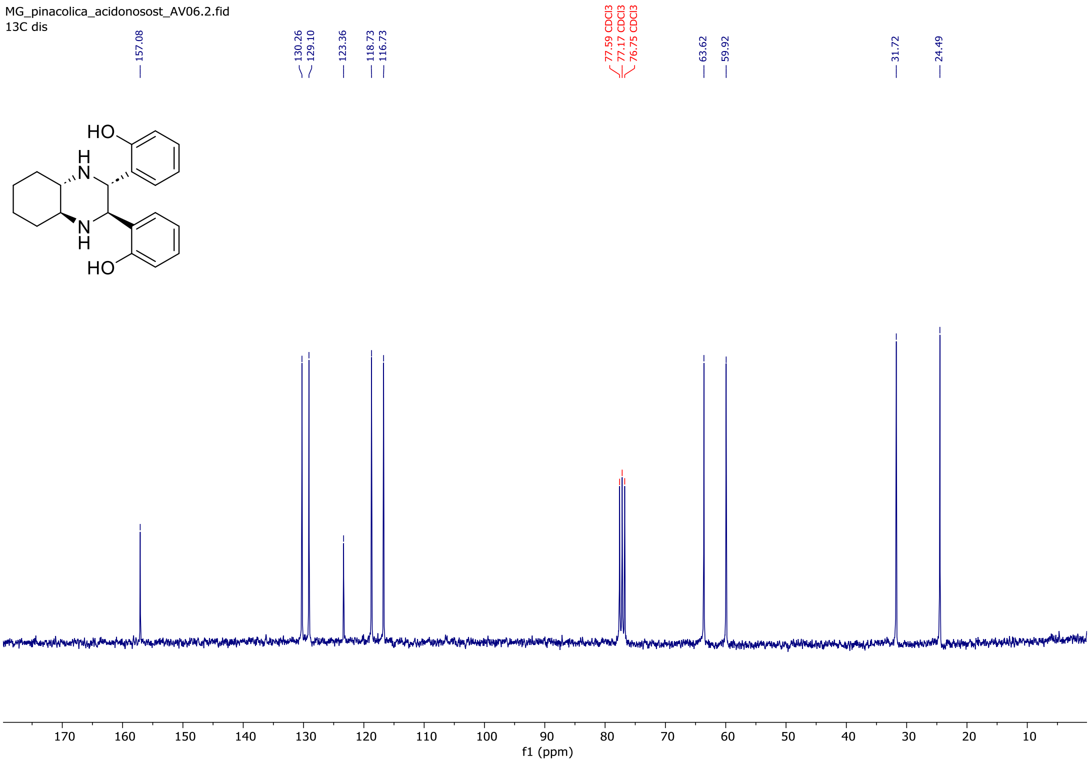

^{1}H NMR compound 2b

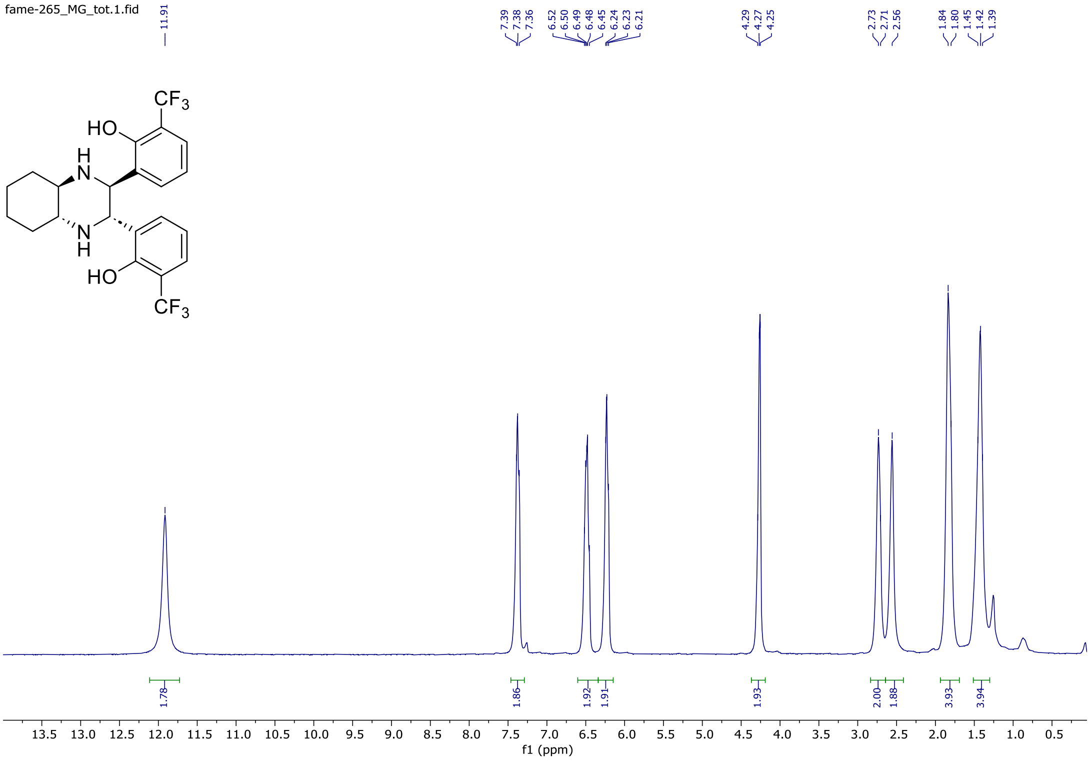

^{13}C NMR compound 2b

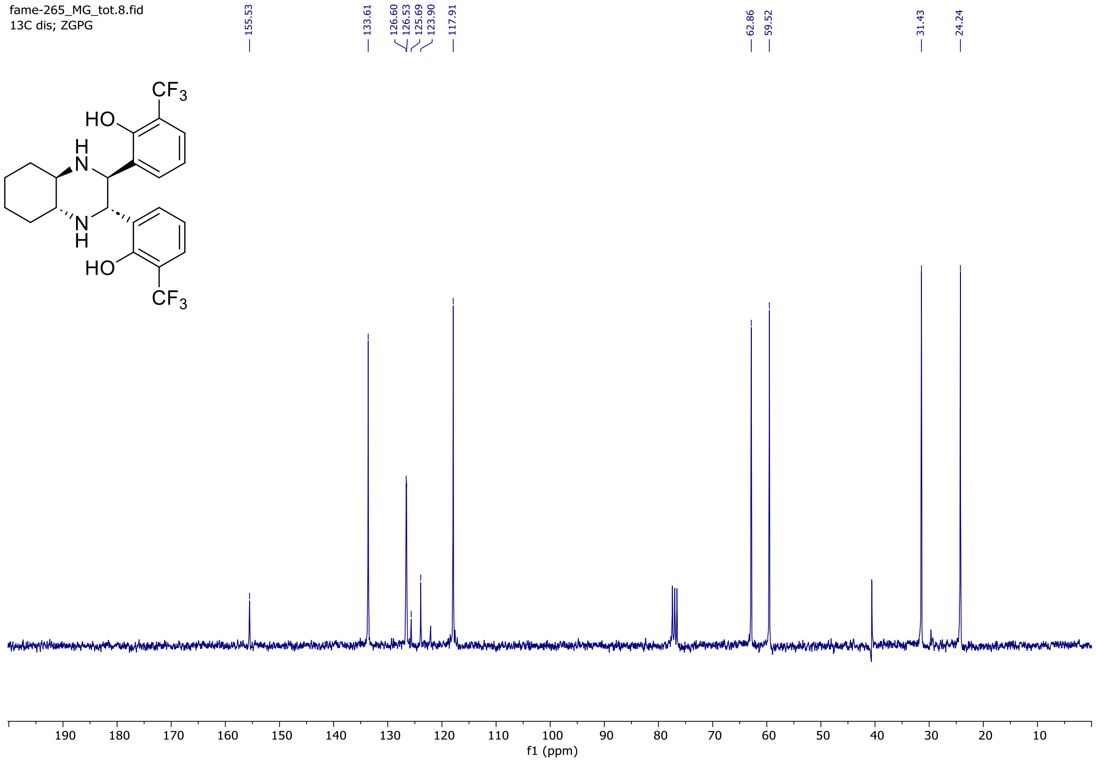

^{19}F NMR compound 2b

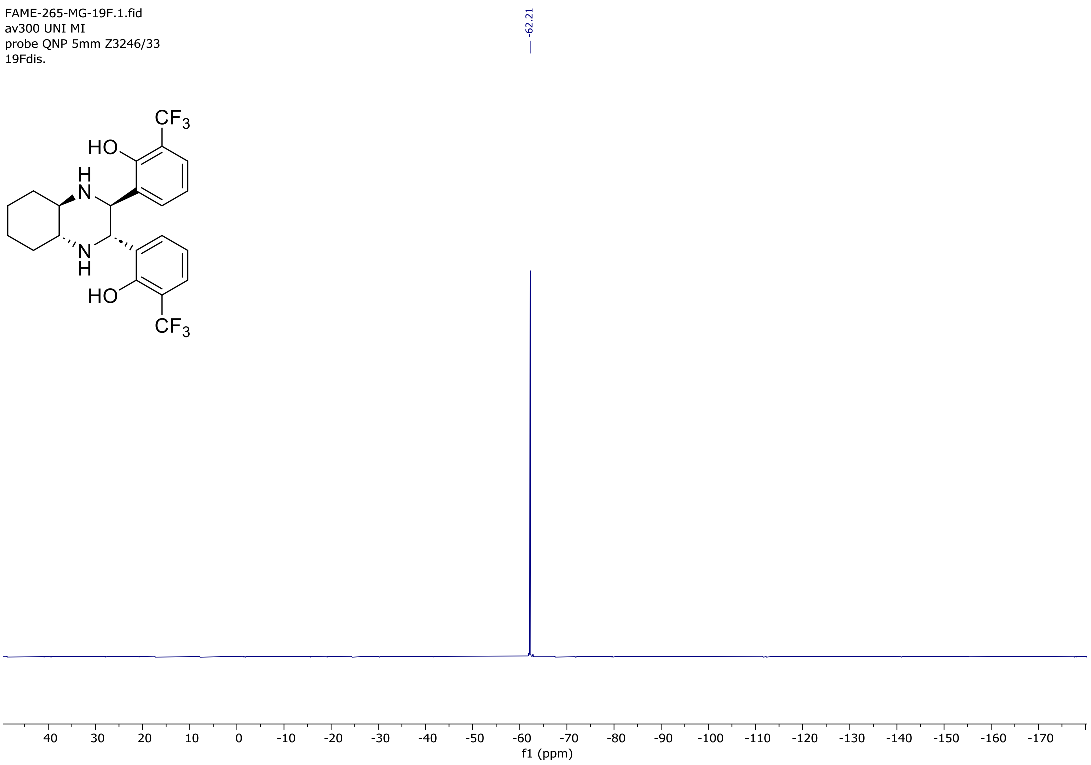

^{1}H NMR compound 2c

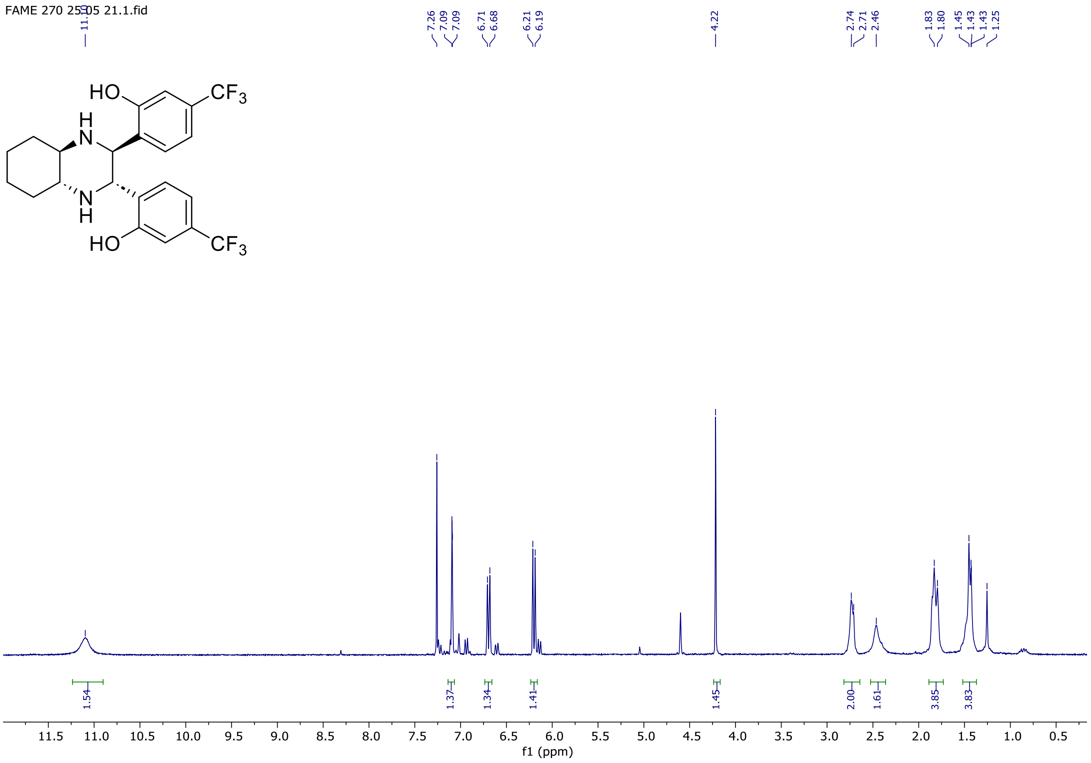

^{13}C NMR compound 2c

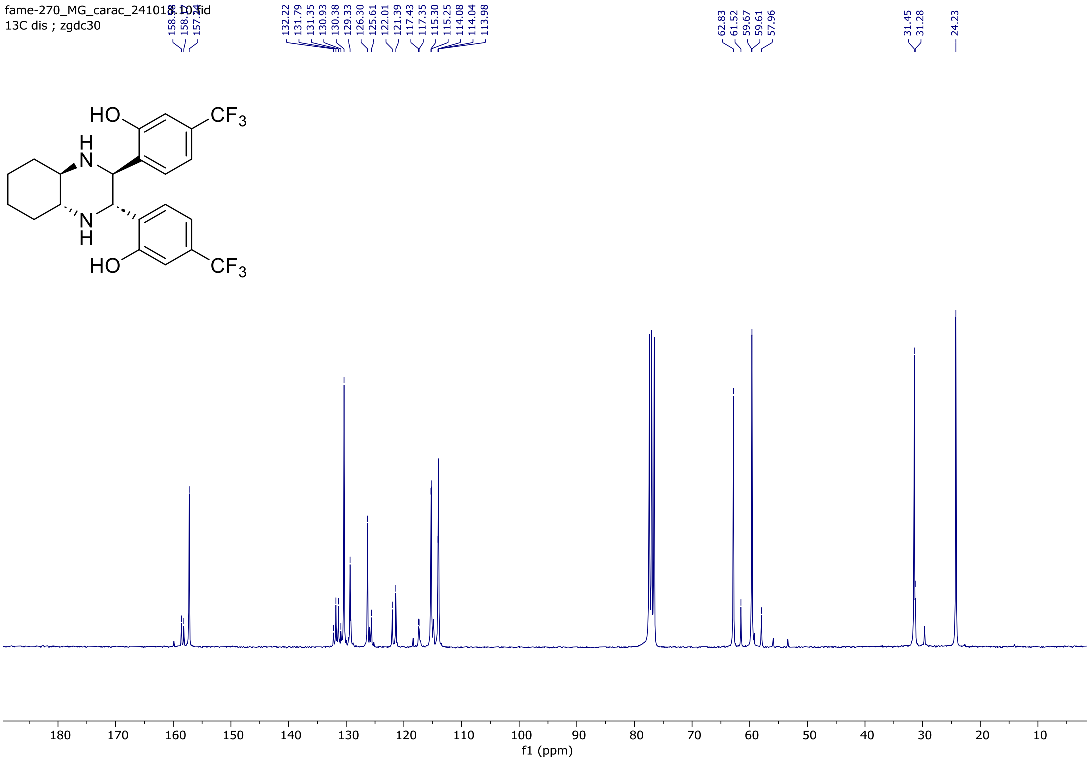

^{19}F NMR compound 2c

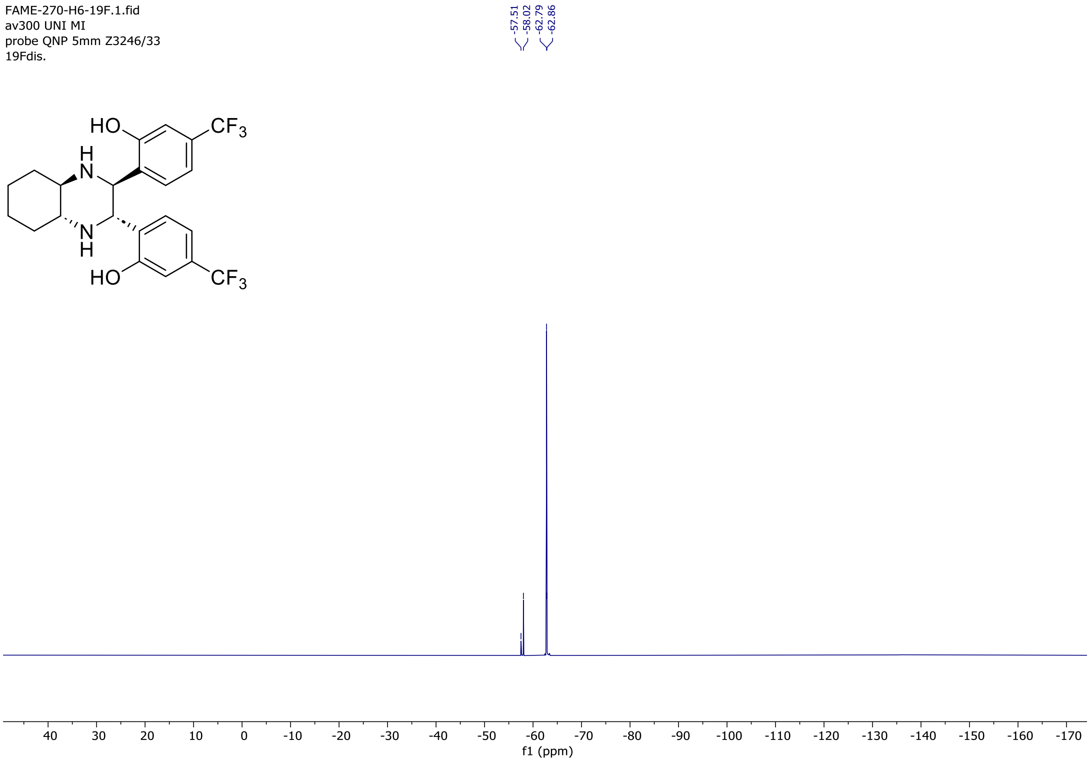

^{1}H NMR compound 2d

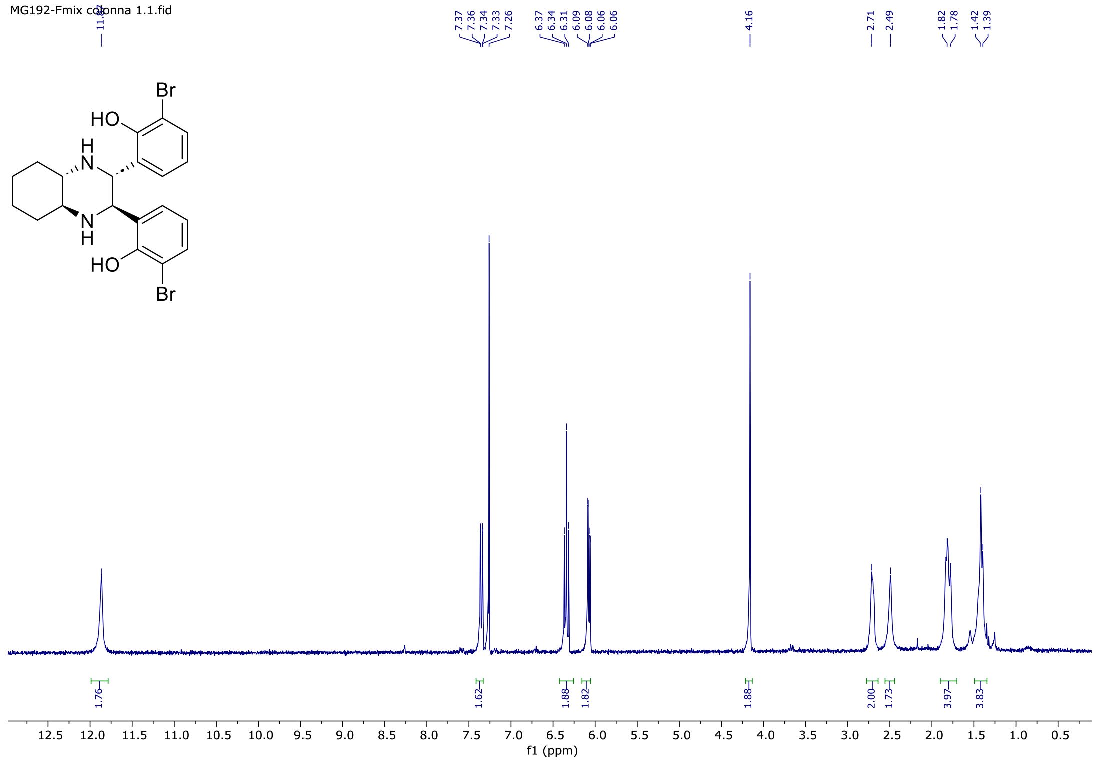

^{13}C NMR compound 2d

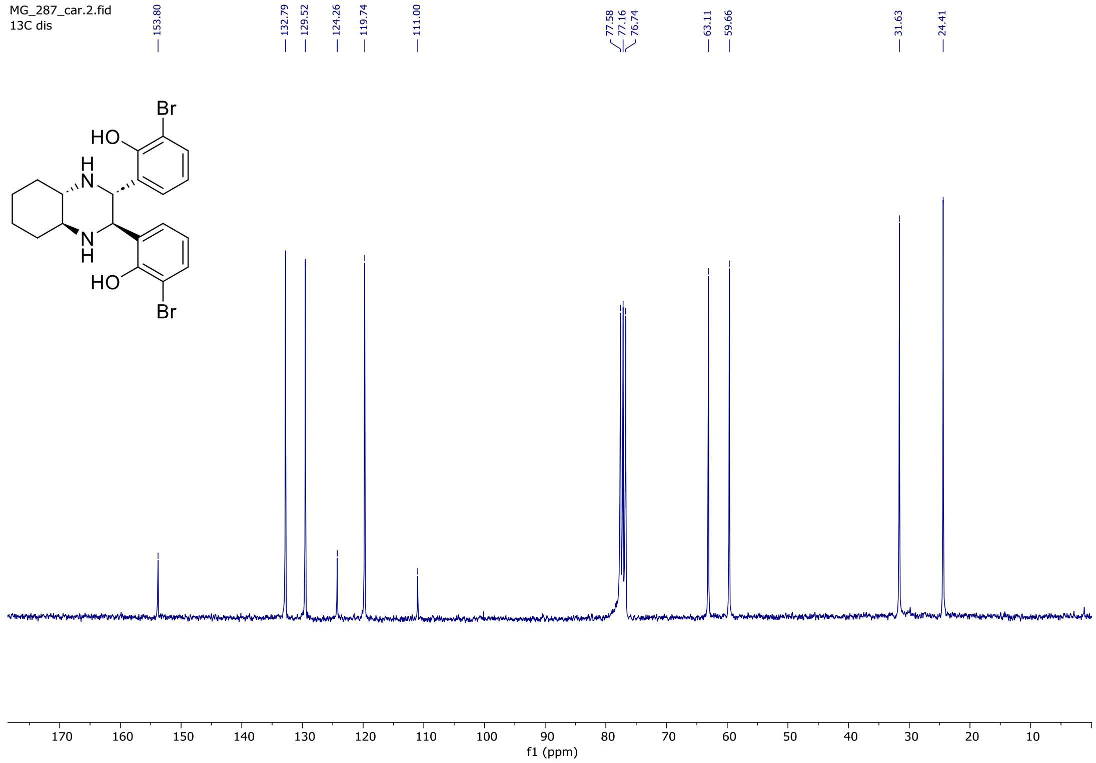

^{1}H NMR compound 2e

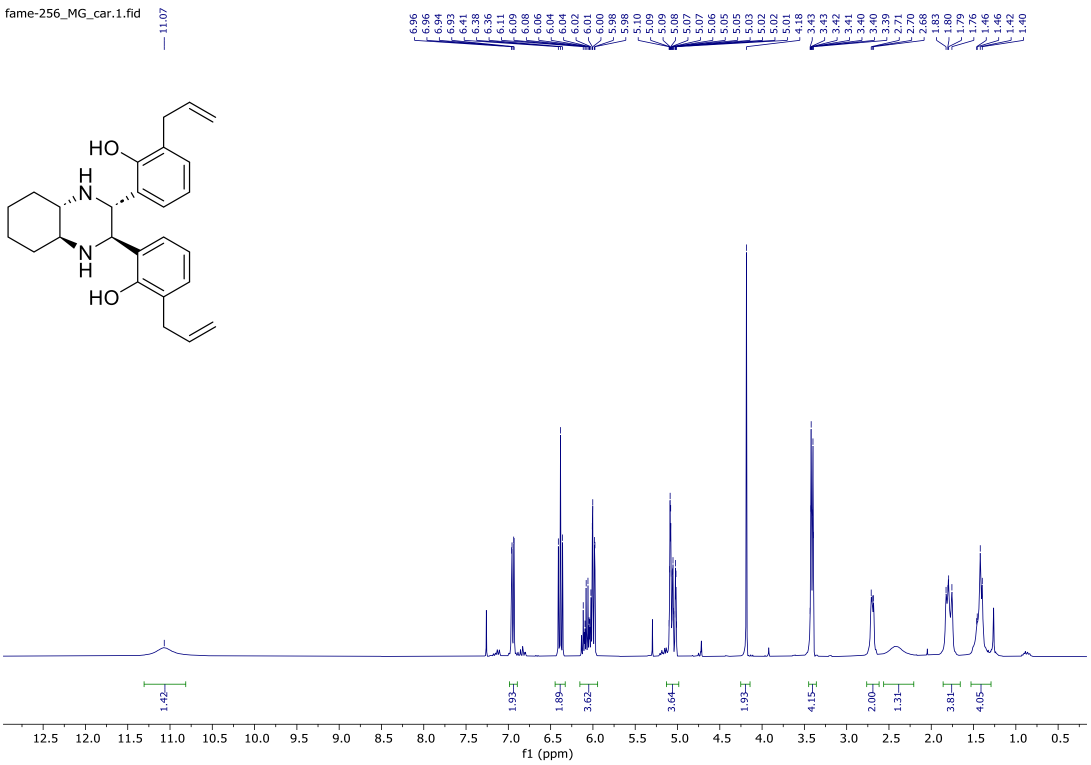

^{13}C NMR compound 2e

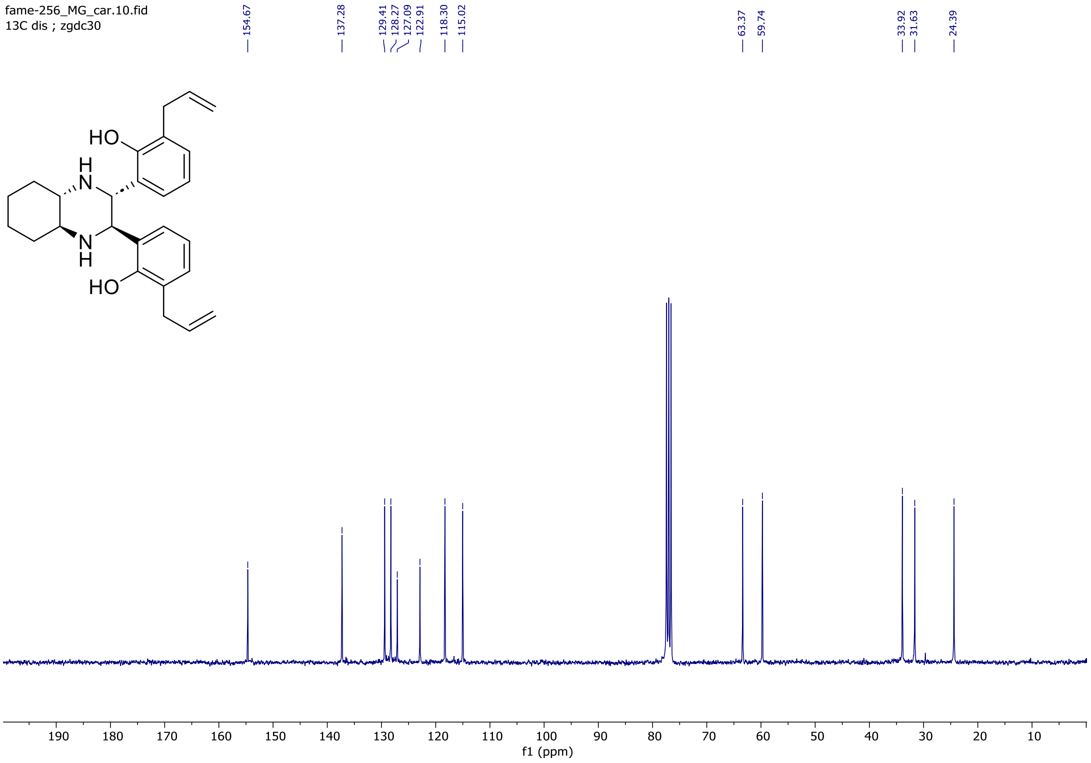

^{1}H NMR compound 2f

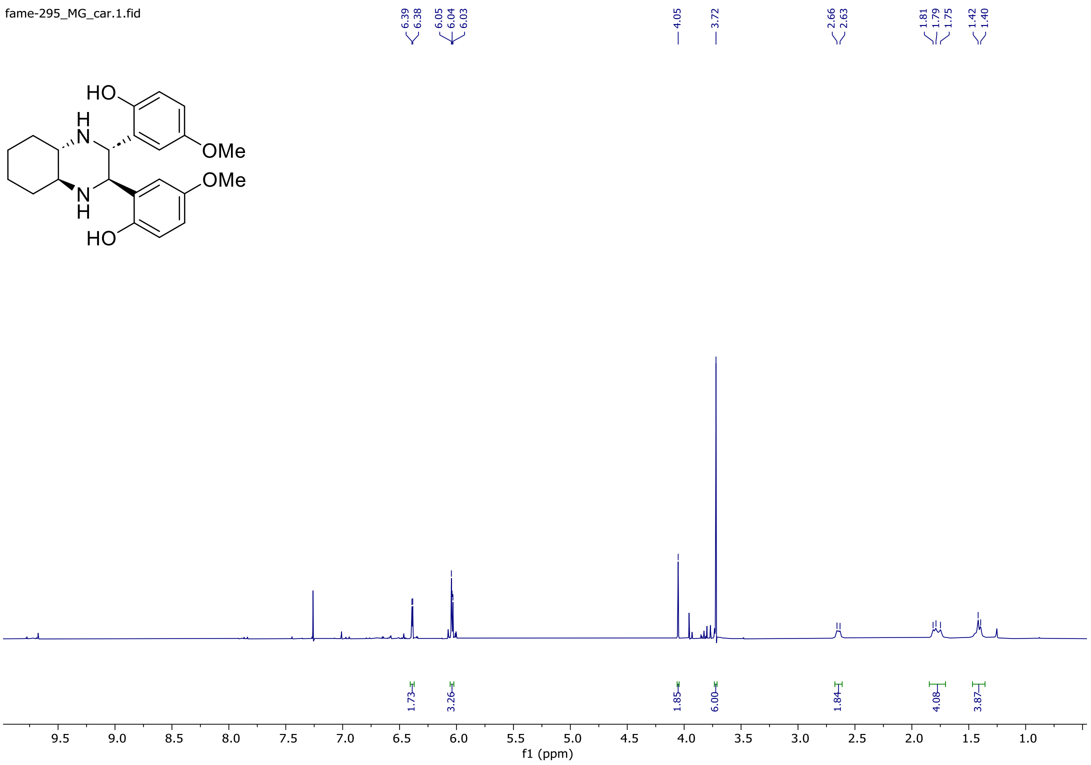

^{13}C NMR compound 2f

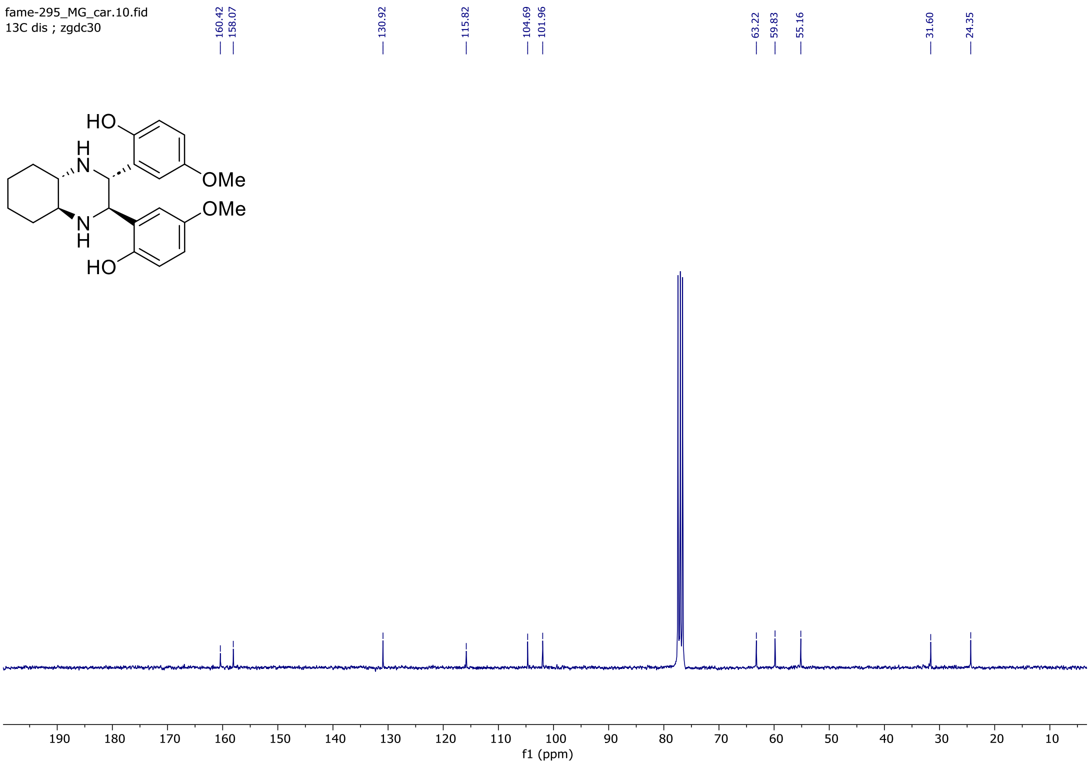

^{1}H NMR compound 2g

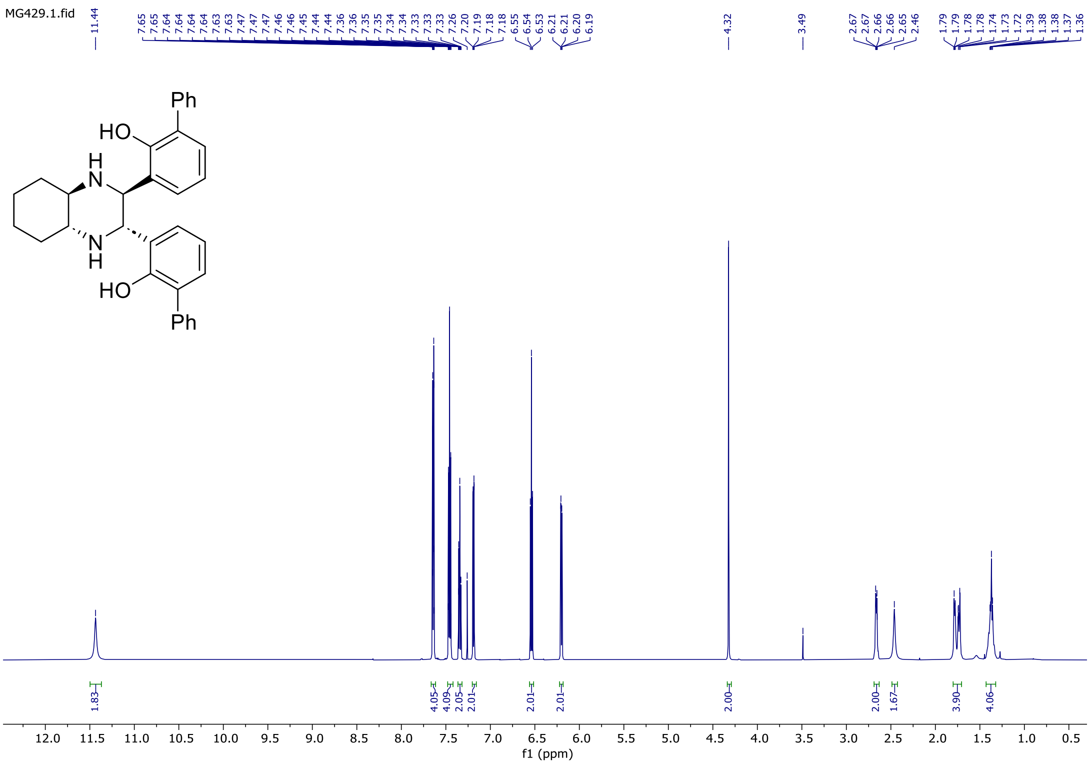

^{13}C NMR compound 2g

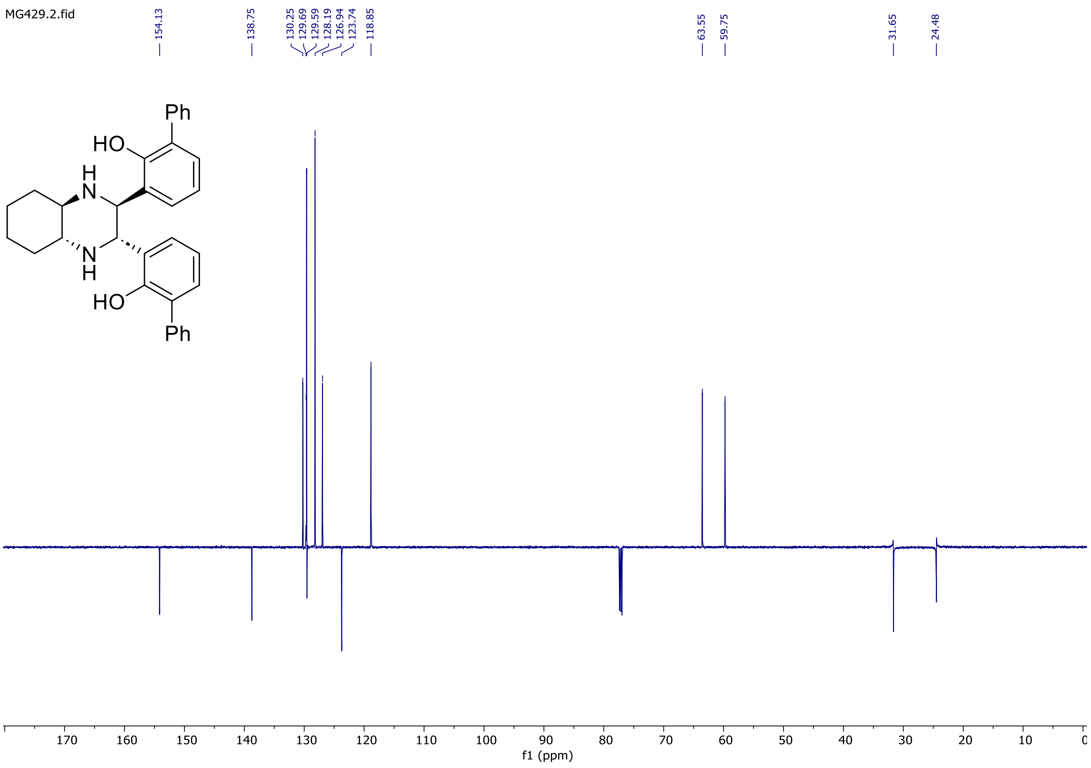

^{1}H NMR compound 2h

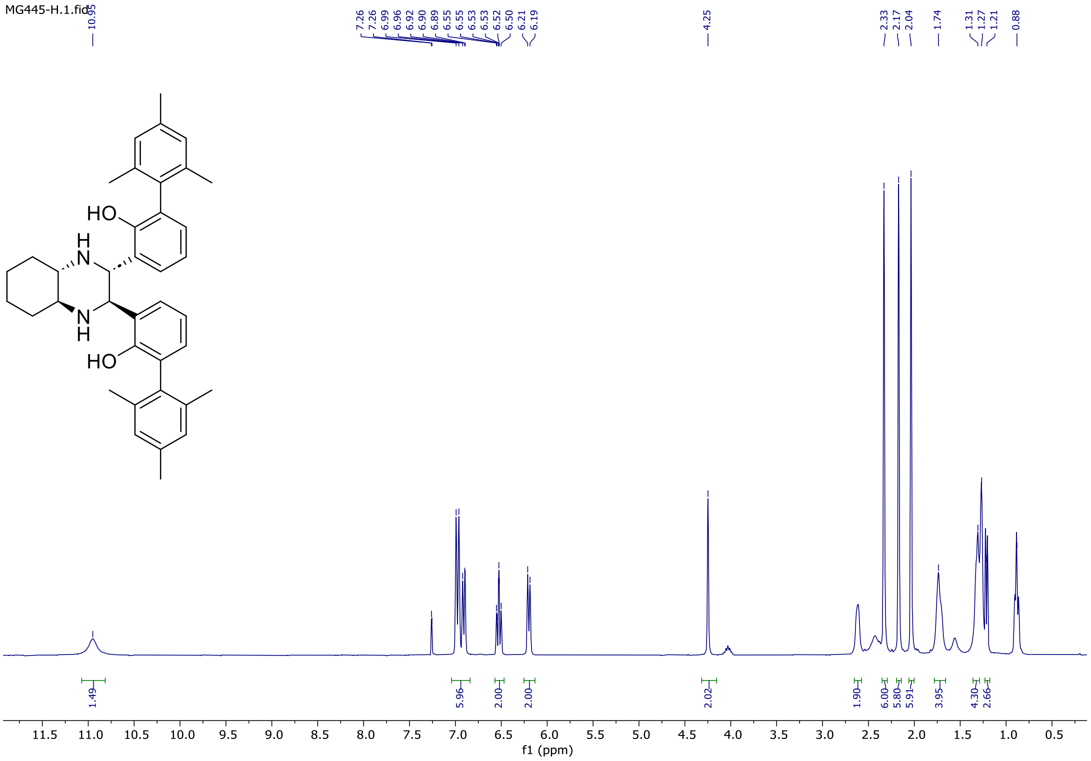

^{13}C NMR compound 2h

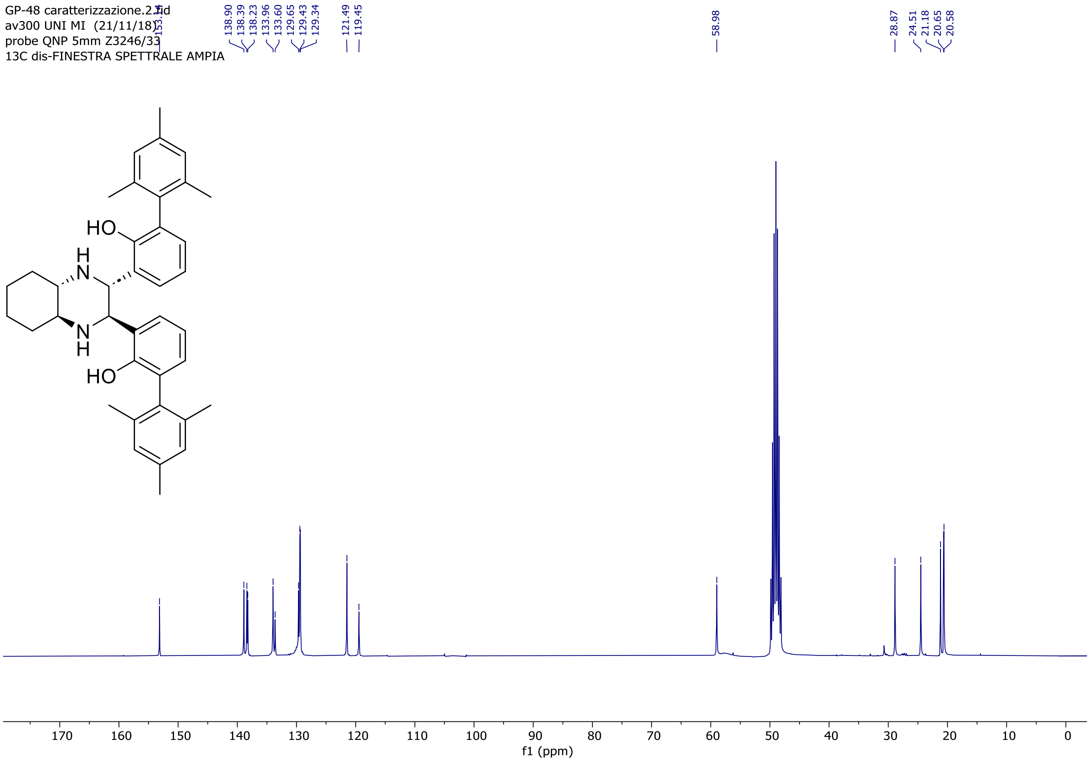

^{1}H NMR compound 2i

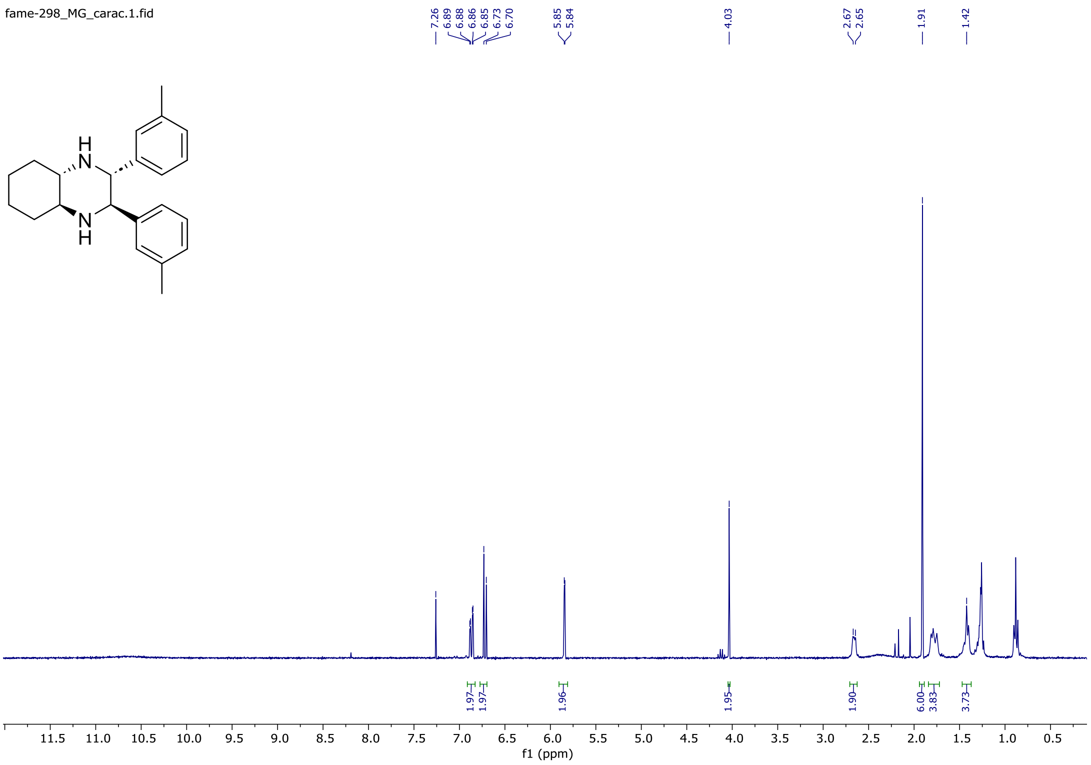

^{13}C NMR compound 2i

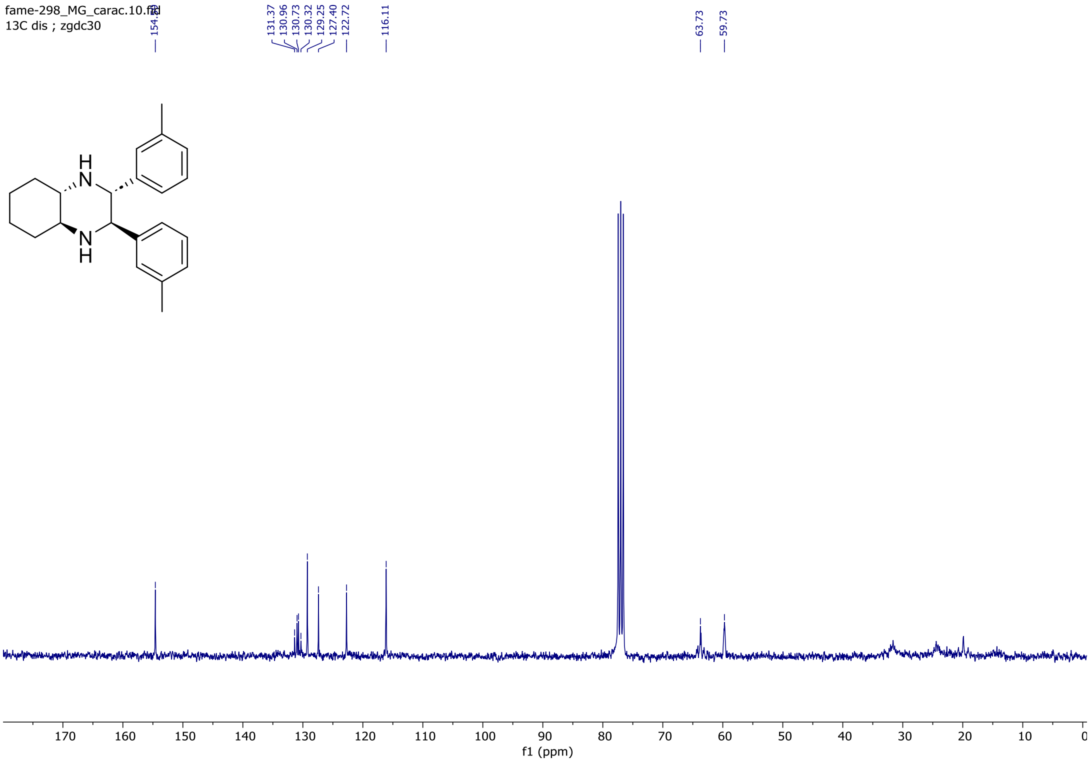

^{1}H NMR compound 2j

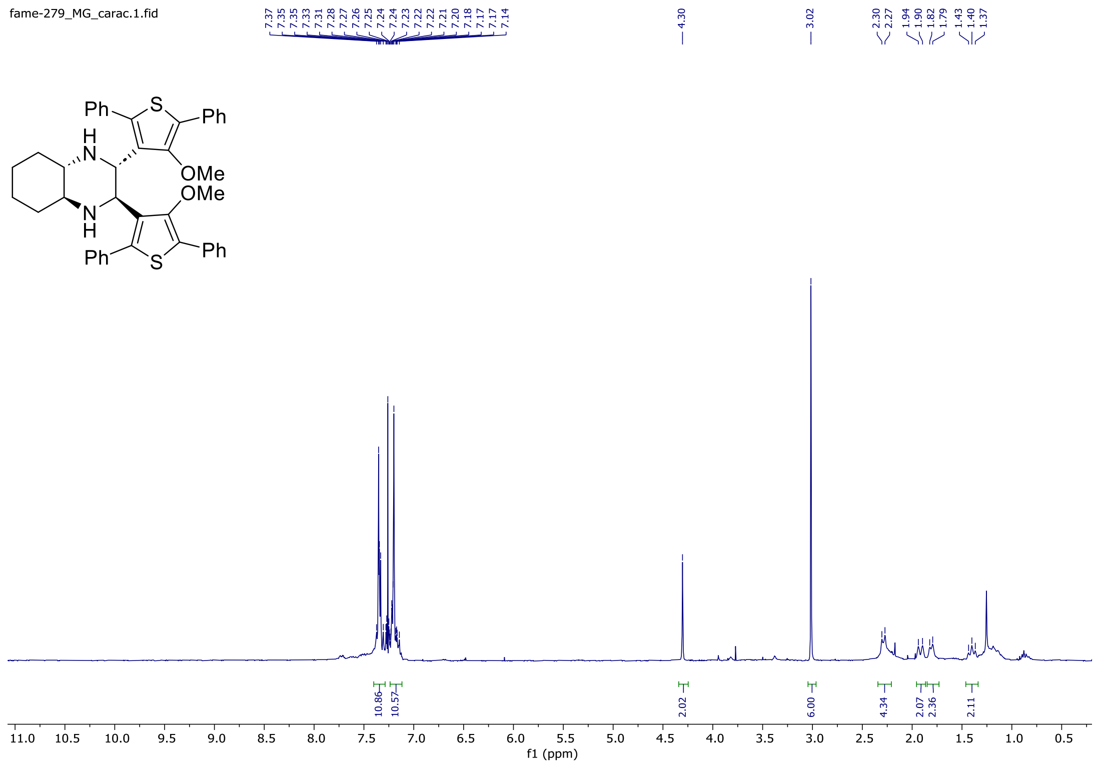

^{13}C NMR compound 2j

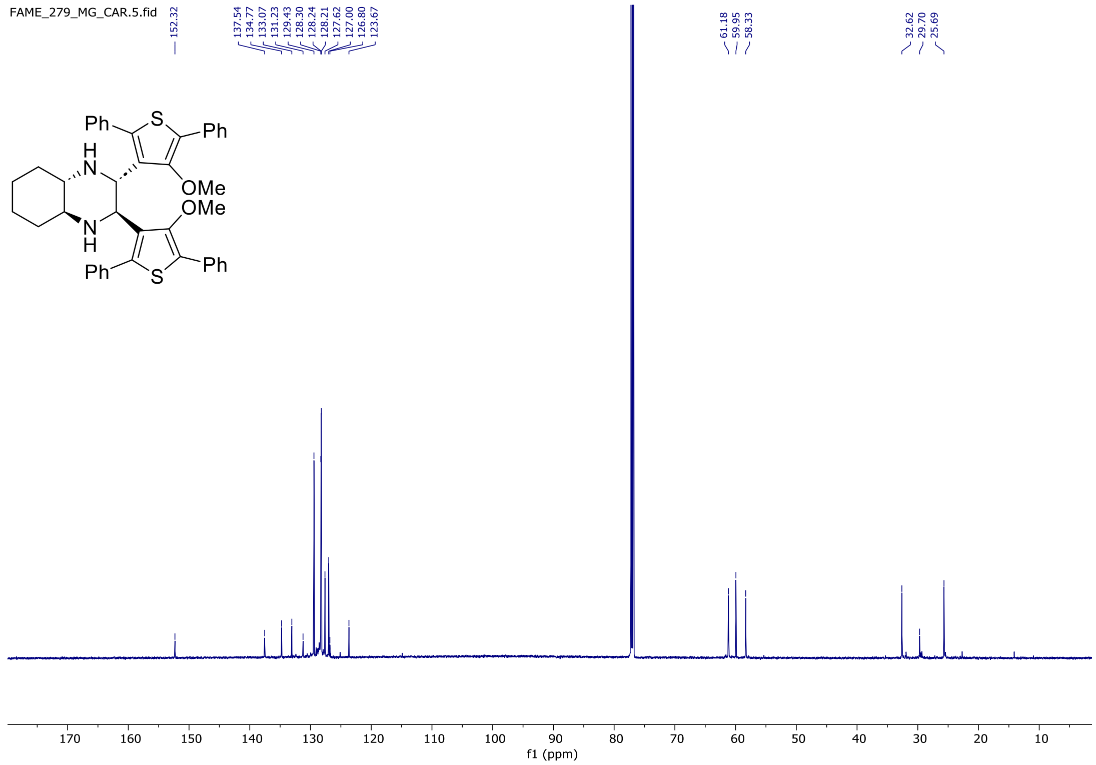

^{1}H NMR compound 2k

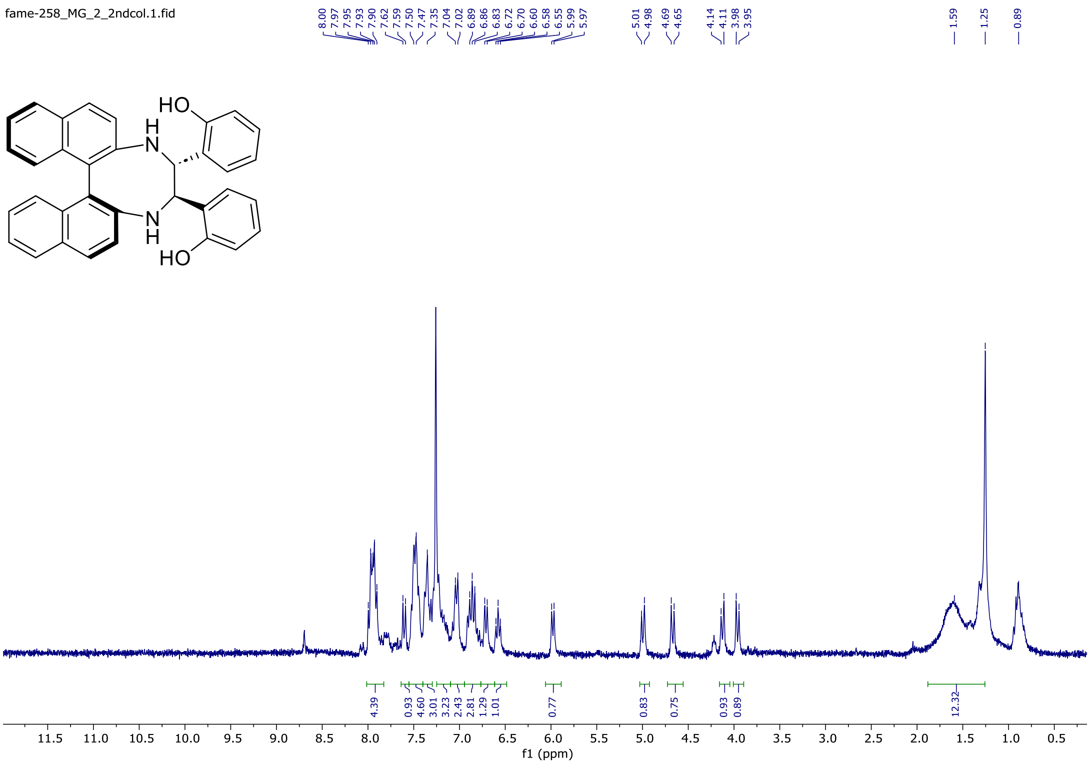

HRMS compound 2a

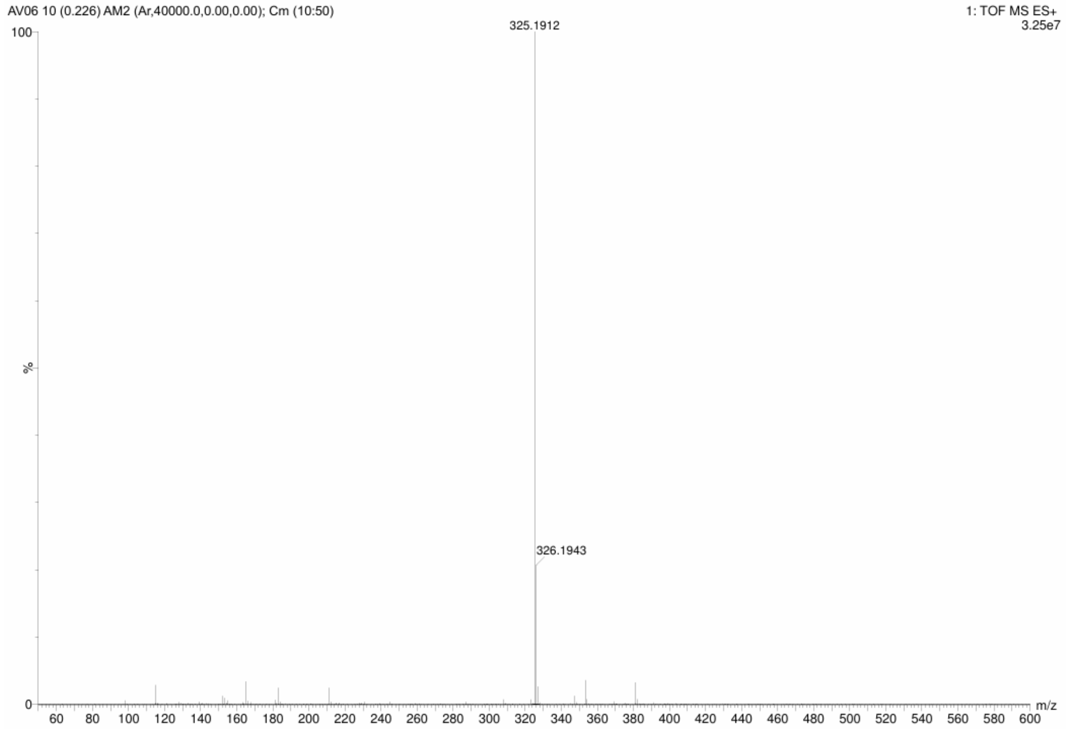

HRMS compound 2b

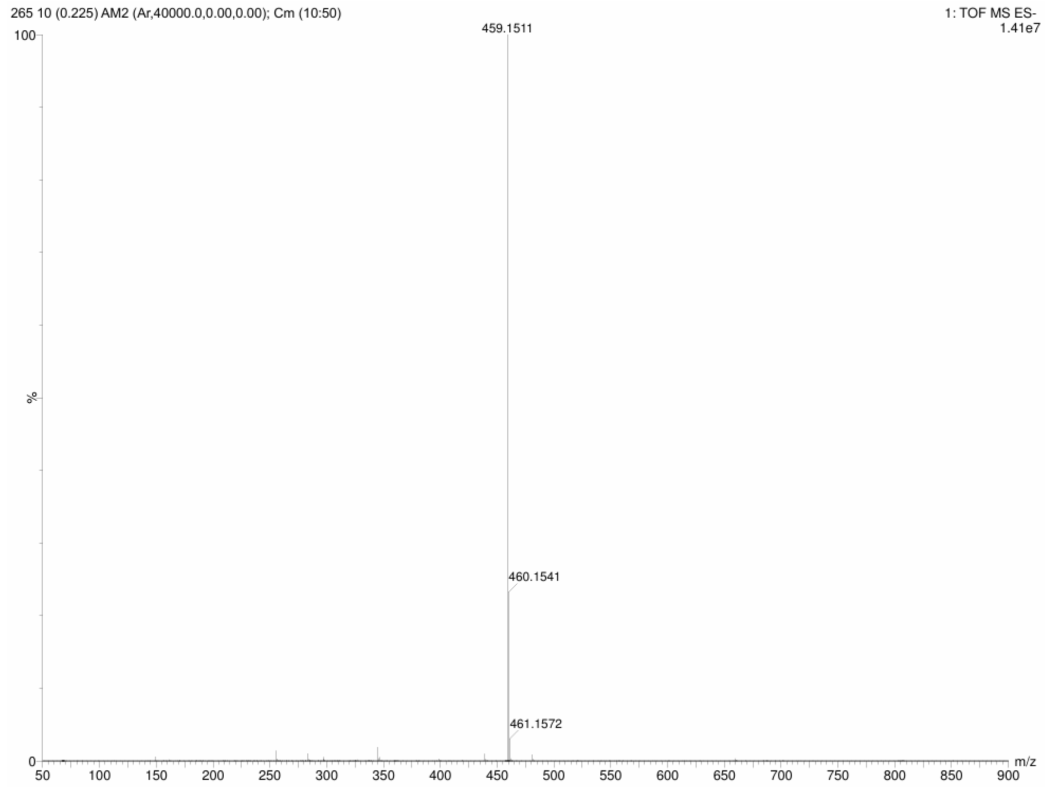

HRMS compound 2c

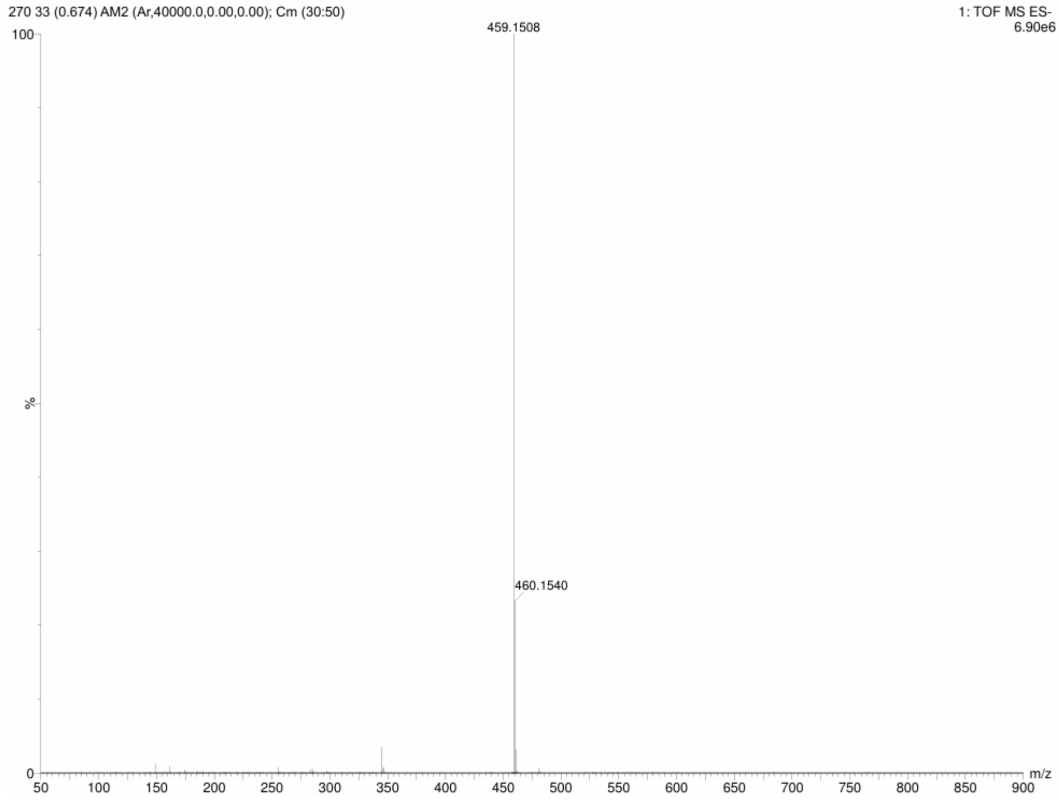

HRMS compound 2d

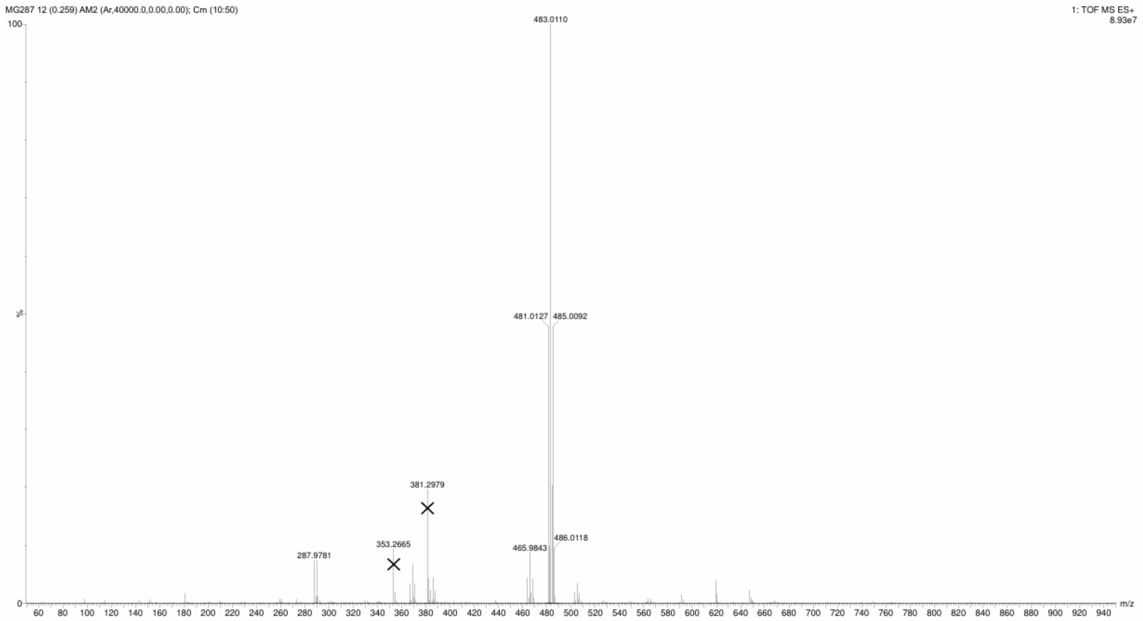

HRMS compound 2e

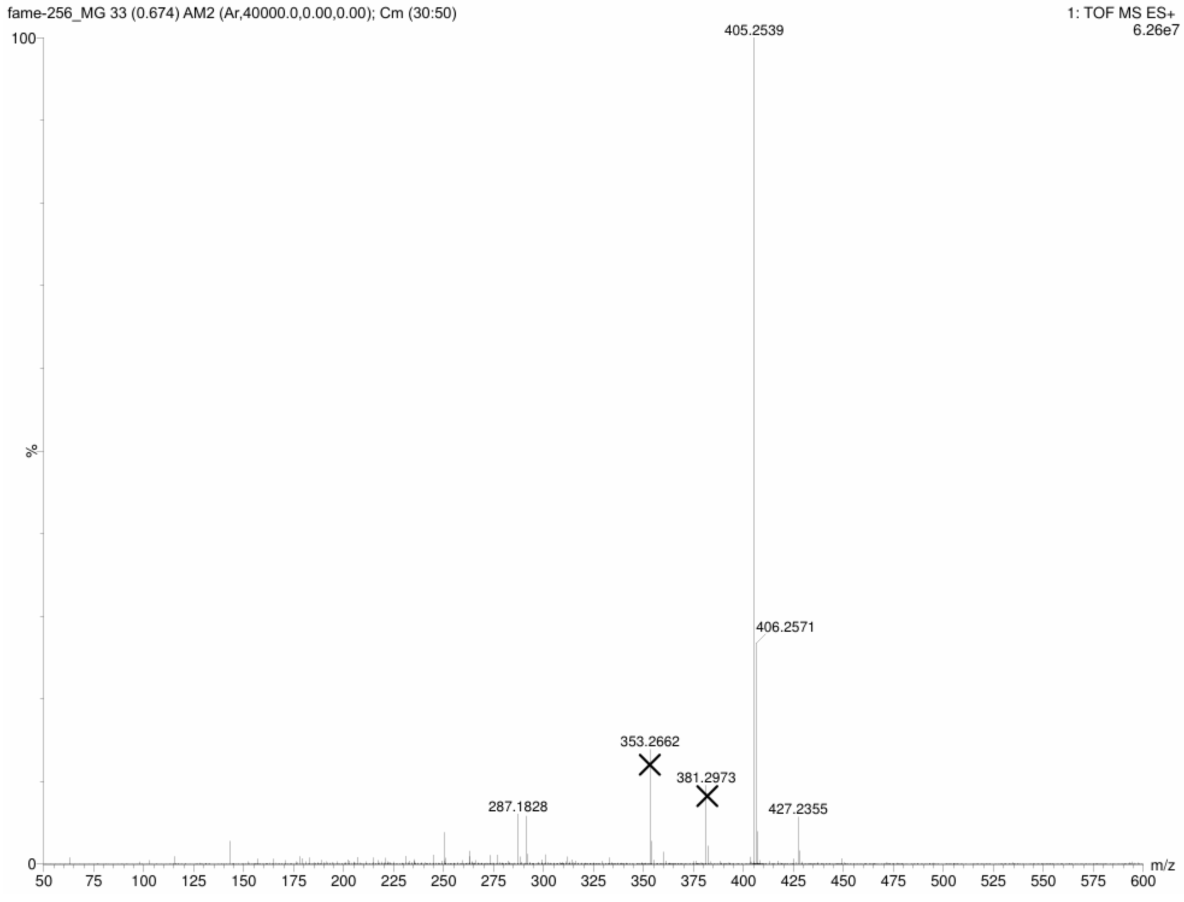

HRMS compound 2f

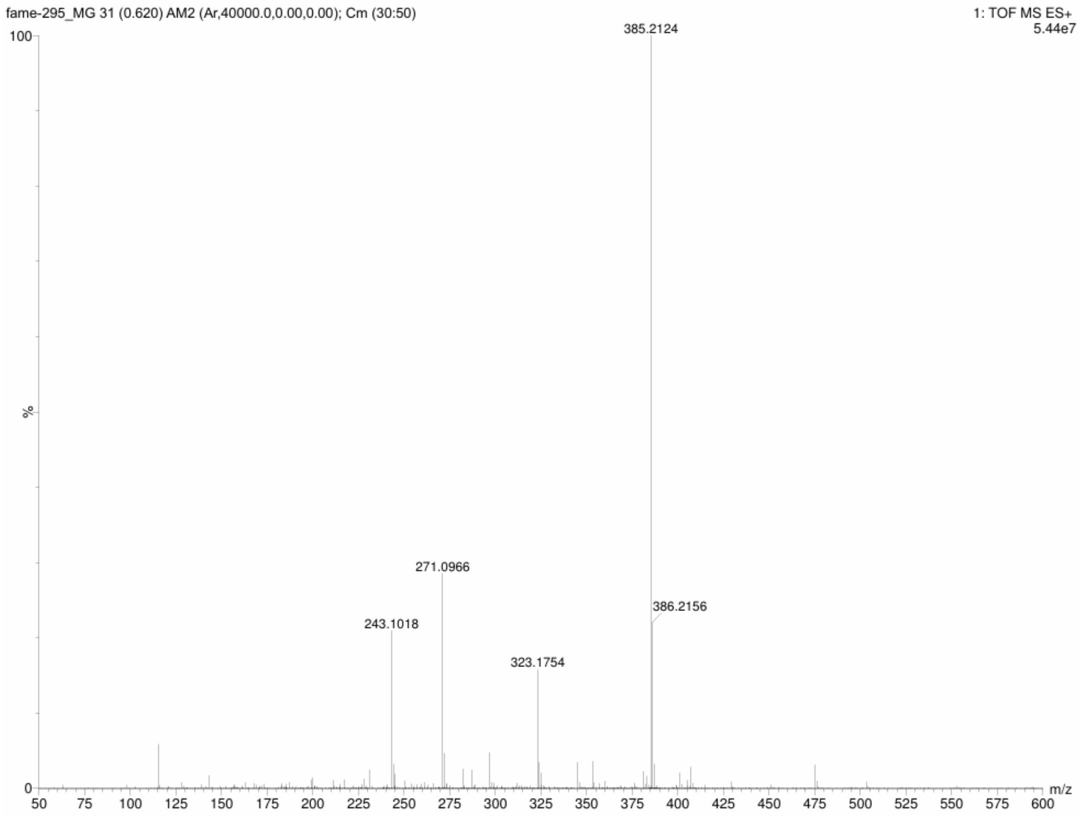

HRMS compound 2g

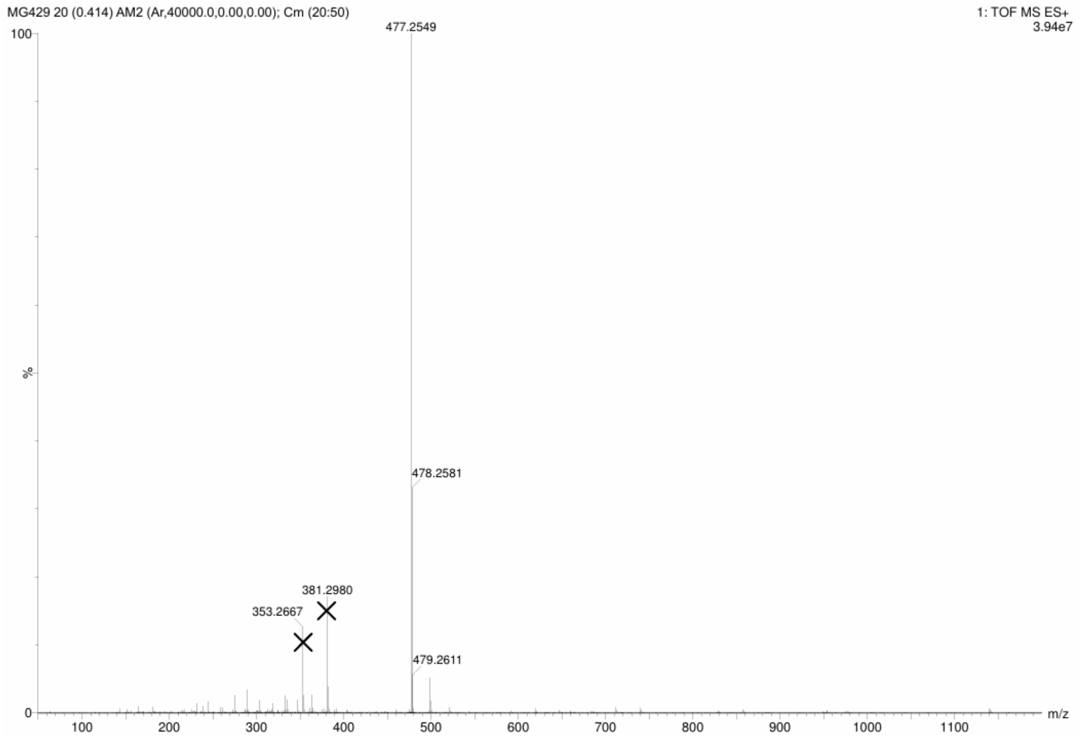

HRMS compound 2h

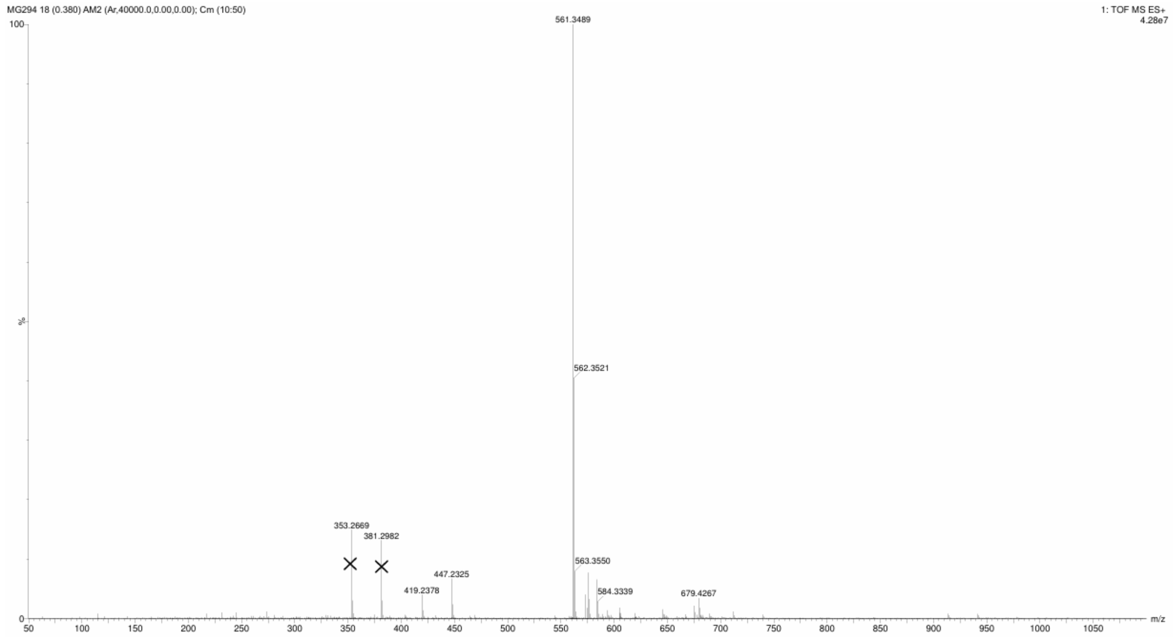

HRMS compound 2j

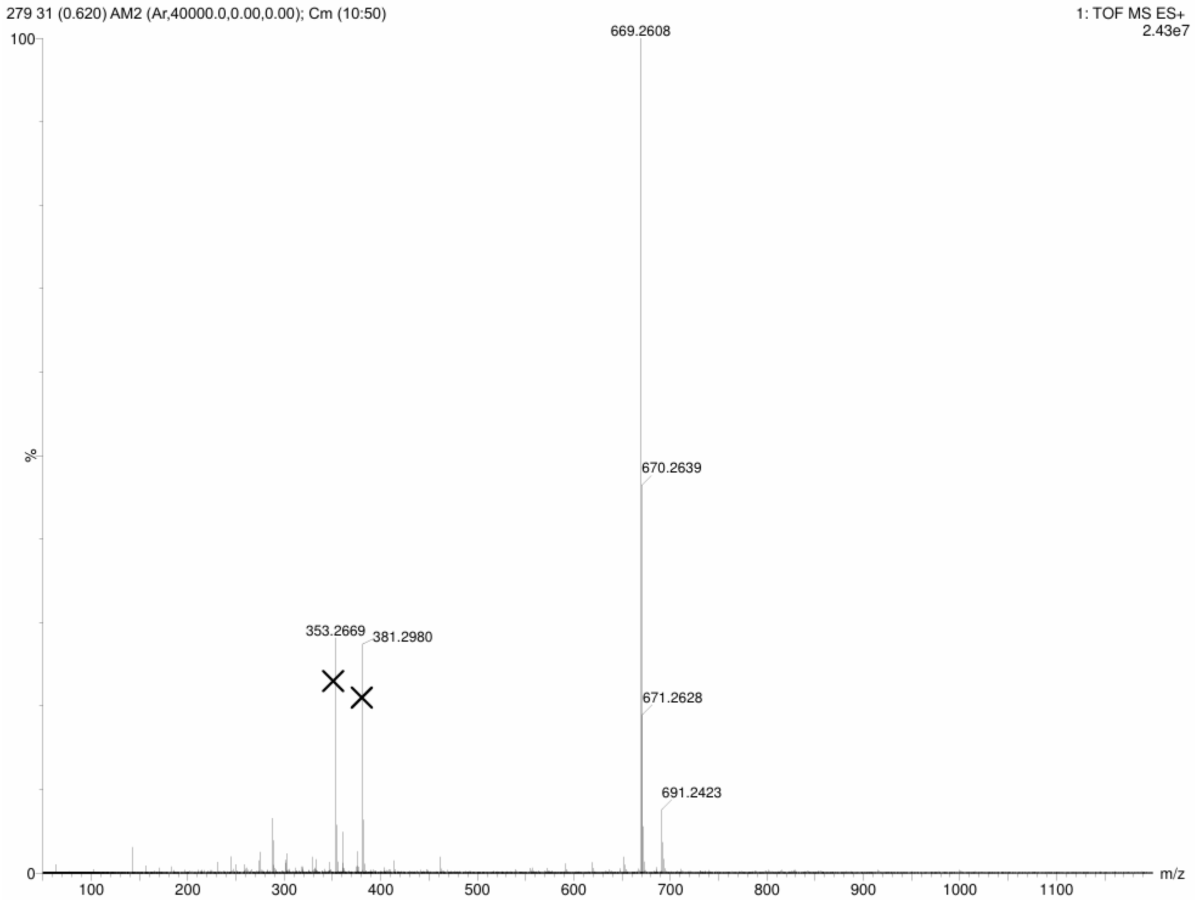

7. Crystallographic data (CCDC deposit number: 2430018)

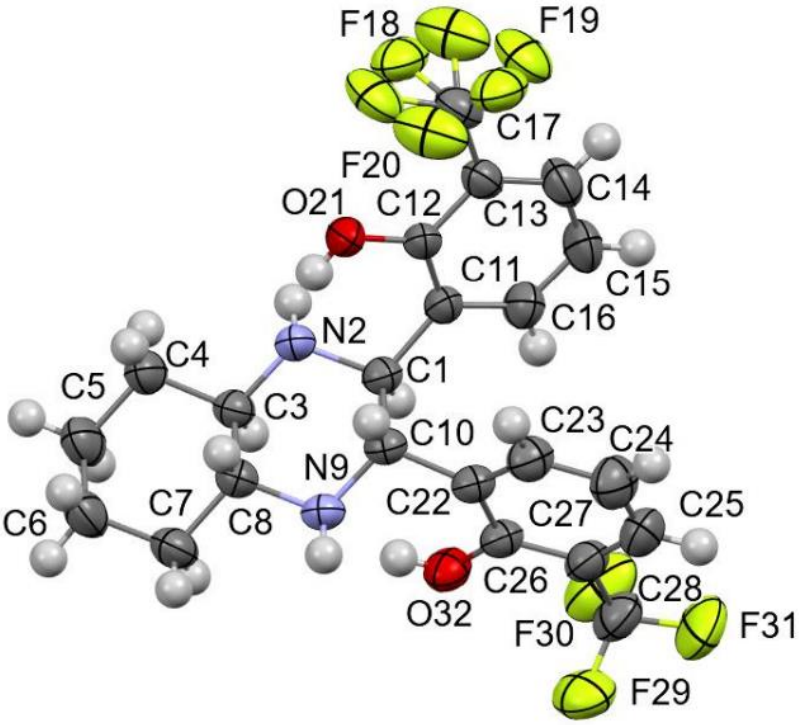

Figure S2. Asymmetric unit (a) and full molecule (b) of **2b** at rt, with the atom-numbering scheme. Thermal ellipsoids of non-H atoms are drawn at the 50 % probability level. The usual colour code was employed for atoms (grey: C; white: H; blue: N; red: O; light green: F). The trifluoromethyl group at C17 is rotationally disordered over two positions with absolute populations of ≈95% and 5%.

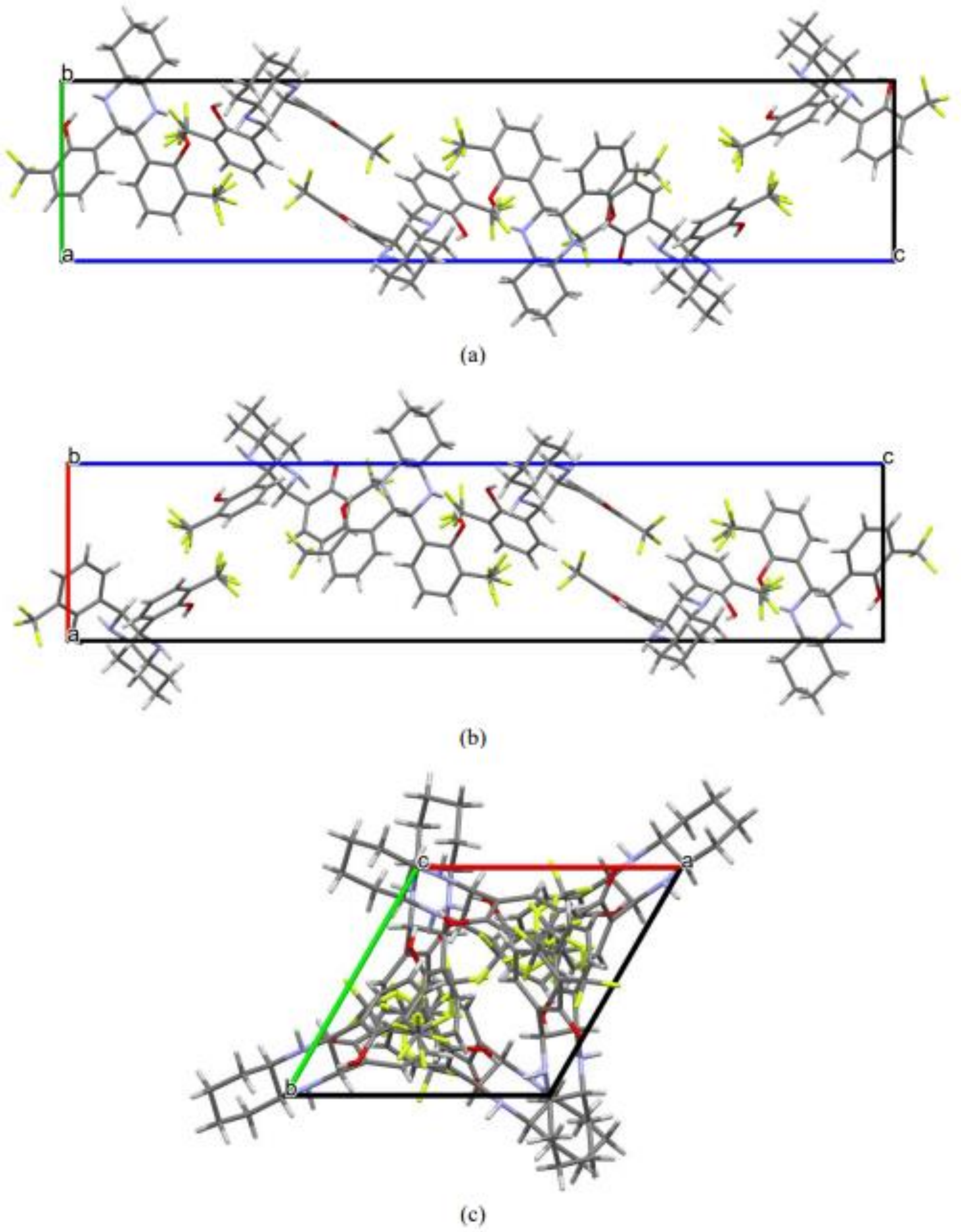

Figure S3. Crystal packing of **2b** at RT, as seen (a) along the *a* cell axis; (b) the *b* cell axis; (c) the *c* cell axis. Colour code as in Figure S2. The crystallographic reference system is also shown.

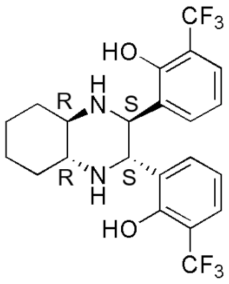

Figure S4. Absolute structure of **2b**, with the CIP descriptors highlighted.

1. The compound is chiral and crystallizes in the acentric apolar space group $P3_12$ as a pure enantiomer, with a total of six molecules in the unit cell and one molecule per asymmetric unit. Figures S2 and S4 show the absolute configuration of the chiral centers. The corresponding configurational descriptors are C1(S), C10(S), C3(R) and C8(R) (see Figure S2 for the atom numbering). The absolute stereochemistry is unequivocally assessed by anomalous dispersion effects. The corresponding Flack parameter reads −0.01(3), as computed from 1617 intensity quotients [(I+)-(I-)]/[(I+)+(I-)] according to Parsons, Flack and Wagner, Acta Cryst. B69 (2013) 249-259.

2. The puckering analysis (Cremer & Pople, J. Am. Chem. Soc., 97, (1975), 1354-1358) shows that the piperazine and cyclohexane rings adopt essentially perfect chair conformations. The corresponding configurational descriptors (puckering amplitude and polar angles) are Q = 0.569(4), θ = 3.4(4), φ = 24(7) deg for the C3-C4-C5-C6-C7-C8 sequence and Q = 0.593(3), θ = 0.0(3), φ = 16(25) deg for the N2-C1-C10-N9-C8-C3 sequence (Figure S2 for atom numbering).

3. The trifluoromethyl group at C17 is rotationally disordered over two sites at room temperature, which are populated by ≈95% (F17, F18, F19, Figure S2) and ≈5% (F17A, F18A, F19A).

4. Figures S3 and S5 show the main packing motifs of **2b**. The hydroxy groups form quasi-symmetric strong intramolecular OH···N hydrogen bonds, and act as acceptors of intermolecular NH···O hydrogen bonds from the neighbouring moieties. The outcome is a bundle of H-bonded ribbons that run mainly along the *c*-axis (Figure S5). Subsequent H-bond linkages along the chain axis are almost perpendicular one to another. Relevant H-bond geometries are detailed in Table S3. Some intermolecular NH···F contacts are also present, which are likely less significant in determining the crystal cohesive energy.

Table S3. Geometry of relevant NH⋯O and OH⋯N contacts in **2b** at room temperature

D–H⋯A	*d*D–H, Å	*d*H⋯A, Å	*d*D⋯A, Å	αDHA, deg	Symmetry operation
			Intermolecular		
N2–H2⋯O21	0.87(3)	2.41(3)	3.131(3)	142(3)	2-x,1-x+y,1/3-z
N9–H9⋯O32	0.91(4)	2.39(3)	3.280(3)	167(3)	y,x,-z
			Intramolecular[a]		
O21–H21⋯N2	0.82	1.91	2.628(3)	145	x, y, z
O31–H32⋯N9	0.82	1.93	2.661(3)	148	x, y, z

[a] The geometry of the OH groups was idealized to neutron diffraction estimates and H positions were refined adopting a riding motion restraint.

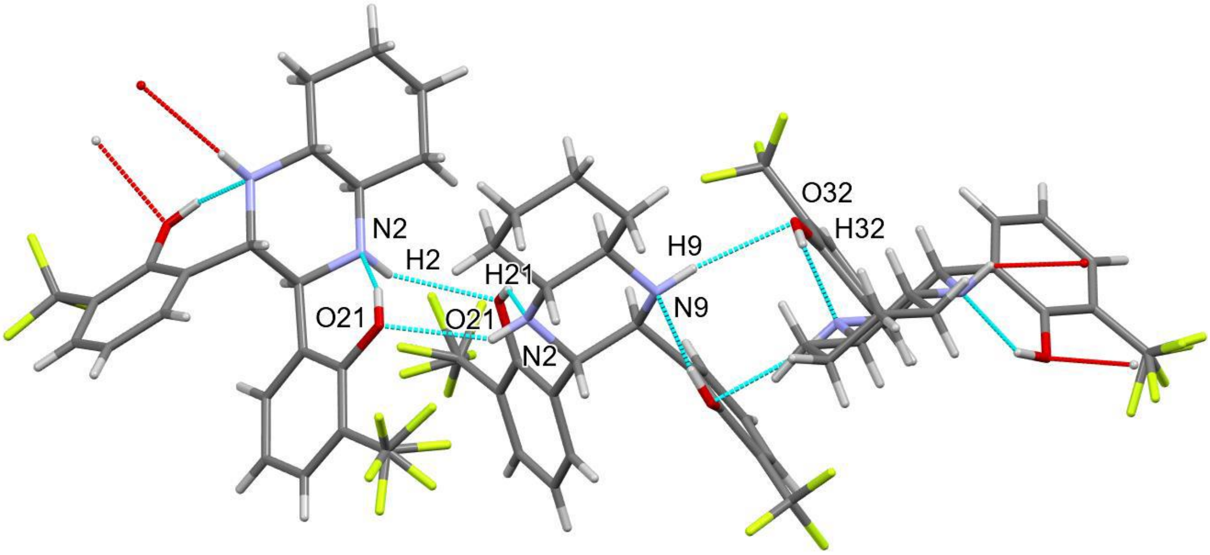

Figure S5. Main H-bonded motif (dashed blue lines) in chiral piperazine **2b**, which continues along the direction indicated by the dashed red lines. Atoms involved in H bonds are highlighted (Table S3). The motif runs mainly along the crystallographic *c* axis.

8. References

1. Senior A.; Ruffell K.; Ball L. T. *Nat. Chem.* **2023**, *15*, 386 – 394. doi:10.1038/s41557-022-01101-0
2. Sacco G.; Stammwitz S.; Belvisi L.; Pignataro L.; Dal Corso A.; Gennari, C. *Eur. J. Org. Chem*. **2021**, *2021*, 1763 – 1767. doi:10.1002/ejoc.202100160
3. Ivanova J.; Abdoli, M.; Nocentini A.; Žalubovskis R.; Supuran C. T. *Journal of Enzyme Inhibition and Medicinal Chemistry* **2023**, *38*, 225 – 238. doi:10.1080/14756366.2022.2142787
4. Stoerkler T.; Pariat T.; Laurent A.D.; Jacquemin D.; Ulrich G.; Massue J. *Eur. J. Org. Chem*., **2022**, e202200661. doi: 10.1002/ejoc.202200661
5. Gabrieli S.; Cirilli R.; Benincori T.; Pierini M.; Rizzo S.; Rossi S. *Eur. J. Org. Chem*. **2017**, *2017*, 861 – 870. doi:10.1002/ejoc.201601353
6. Yanagisawa S.; Sudo T.; Noyori R.; Itami K. *J. Am. Chem. Soc*. **2006**, *128*, 11748 – 11749. doi: 10.1021/ja064500
7. Zhao B.; Wang Z.; Ding K. *Adv. Synth. Catal.* **2006**, *348*, 1049 – 1057. doi: 10.1002/adsc.200606013
8. Colombo F.; Benaglia M.; Orlandi S.; Usuelli F.; Celentano G. *J. Org. Chem.,* **2006**, *71*, 2064 – 2070. doi: 10.1021/jo052481g
